# A revision of the Chinese Stephanidae (Hymenoptera, Stephanoidea)

**DOI:** 10.3897/zookeys.110.918

**Published:** 2011-06-21

**Authors:** Chun-dan Hong, Cornelis van Achterberg, Zai-fu Xu

**Affiliations:** 1 College of Natural Resources and Environment, South China Agricultural University, Guangzhou 510640, P. R. China; 2 Department of Terrestrial Zoology, Netherlands Centre for Biodiversity Naturalis, Postbus 9517, 2300 RA Leiden, Netherlands

**Keywords:** Revision, Stephanidae, *Foenatopus*, *Megischus*, *Parastephanellus*, *Stephanus*, *Schlettererius*, keys, new species, new synonyms, lectotype, China

## Abstract

Stephanidae Leach, 1815 (Hymenoptera: Stephanoidea) from China are revised. Five genera are reported from China: *Foenatopus* Smith, 1861; *Megischus* Brullé, 1846; *Parastephanellus* Enderlein, 1906; *Schlettererius* Ashmead, 1900; and *Stephanus* Jurine (in Panzer), 1801, and the genera are keyed. All the Chinese species are described and illustrated and new synonyms are established. Keys to species of the five genera occurring in China and adjacent regions are provided.

Six species are new to science: *Foenatopus brevimaculatus* **sp. n.**, *Foenatopus maculiferus* **sp. n.**, *Foenatopus yangi* **sp. n.**, *Parastephanellus angulatus* **sp. n.**, *Parastephanellus brevicoxalis* **sp. n.** and *Parastephanellus zhejiangensis* **sp. n.** One species, *Parastephanellus matsumotoi* van Achterberg, 2006, is newly recorded from China.

The following 9 new synonyms are proposed: *Foenatopus aratifrons* Enderlein, 1913 and *Foenatopus yunnanensis* Chao, 1964, new synonymys for *Foenatopus annulitarsus* Enderlein, 1913; *Foenatopus cerviculatus* (Chao, 1964) and *Foenatopus chaoi* Belokobylskij, 1995 for *Foenatopus chinensis* (Elliott, 1919); *Foenatopus formosanus* Enderlein, 1913 for *Foenatopus cinctus* (Matsumura, 1912); *Foenatopus simillimus* (Elliott, 1920) and *Foenatopus trilineatus* (Elliott, 1920) for *Foenatopus flavidentatus* (Enderlein, 1913); *Foenatopus trilobatus* (Elliott, 1920) for *Foenatopus ruficollis* (Enderlein, 1913); *Parastephanellus austrochinensis* Belokobylskij, 1995 for *Parastephanellus brevistigma* Enderlein, 1913. A lectotype is designated for *Diastephanus trilineatus* Elliott, 1920.

## Introduction

The family Stephanidae Leach, 1815, is a cosmopolitan family with 345 extant species (van Achterberg and Yang 2004; van Achterberg and Quicke 2006; Aguiar 2004, 2006; Aguiar and Jennings 2005; Aguiar et al. 2010; Hong et al. 2010; Hong and Xu 2011). It is considered to be the most basal group of the Hymenoptera-Apocrita and occurs mainly in subtropical and tropical forests (Vilhelmsen 1997; van Achterberg 2002), but some species occur in subtropical and moderate climate zones. The species of Stephanidae are usually medium-sized to large, and the largest species are in the genus *Megischus*, with body length reaching up to 35 mm. Stephanidae are conspicuous by the “crown” on the head, the more or less modified pronotum, the shape of hind legs (especially the more or less swollen hind femur with ventral large teeth, the hind tibia widened apically), the often present ivory streaks on the frons or temple and ivory or whitish subapical band of the ovipositor sheath in some genera (van Achterberg 2002; van Achterberg and Yang 2004).

Stephanidae are generally considered to be rare or extremely rare, and nearly 95% of all stephanid species were described from a single specimen. The systematics of the Stephanidae is imperfectly known; many types have never been studied since their descriptions and they exhibit a rich, but often continuous morphological variation. Stephanidae are not easily collected by traditional methods (Aguiar 2004). Sweeping, Malaise traps and yellow pan traps, which are all staple methods for collecting many Hymenoptera, seem ineffective for stephanids. Aguiar and Sharkov (1997) suggested that the use of blue pan traps could be an effective trapping technique for Stephanidae; however, only seven stephanids were collected in 39 blue pan traps and further evidence is needed to prove the potential of such traps in the Old World. In total, three hundred and three specimens of Stephanidae from China and adjacent regions have been studied and 21 species are recognized to occur in China, of which 6 species (or 29%) are new to science.

The actual biology of nearly all Stephanidae species is unknown or nearly unknown and only *Schlettererius cinctipes* and *Stephanus serrator* are recorded from several hosts. Stephanidae can be found around tree trunks or branches of trees dead for about one year, which are inhabited by beetle larvae and not yet infested by fungi (van Achterberg 2002). Stephanidae are solitary idiobiont ectoparasitoids of wood boring insect larvae and their hosts are mainly Cerambycidae and Buprestidae. Other recorded hosts belong to other families of Coleoptera such as Curculionidae, and to Hymenoptera, Siricidae larvae as well as solitary bees (Taylor 1967; van Achterberg 2002; Aguiar 2004).

*Megischus ptosimae* was reported as parasitoid of larvae of *Ptosima chinensis* Mars in peach trees (Chao 1964); *Schlettererius cinctipes* was once introduced from California to Tasmania to prove its potential value as a member of the parasitoid complex on *Sirex nectilio* (Taylor 1967); *Stephanus bidentatus* and *Stephanus tridentatus* were found on trunks of *Quercus* and *Toxicodendron* trees with Cerambycidae larvae and *Stephanus tridentatus* was ovipositing in Buprestidae larvae in an *Ulmus* tree (van Achterberg and Yang 2004). Obviously, Stephanidae can be of importance in biological control of coleopteran and hymenopteran pests.

Stephanids were classified with other distantly related wasp clades or families for a long time since they are somehow externally similar with some Ichneumonoidea. Leach was the first to propose a separate family for stephanids in 1815, but “Stephanidae” was first used by Haliday in 1839. The superfamily, Stephanoidea, was originally proposed by Benoit in 1949. The family reached its current limits when Rasnitsyn excluded the “Stenophasmidae” (Braconidae) from the superfamily in 1969.

Recent main classification efforts on Stephanidae on a world-wide basis are as follows: van Achterberg (2002) revised the Old World species of several genera, established three new genera and provided a key to the genera of the family; Aguiar (2004) summarized the taxonomic history and classification of the family and catalogued the genera and species.

Presently about 13 valid genera and 351 valid species (including 6 extinct species in 2 extinct genera) of Stephanidae are known worldwide (van Achterberg and Yang 2004; van Achterberg and Quicke 2006; Aguiar 2004, 2006; Aguiar and Jennings 2005, 2010; Aguiar et al. 2010; Hong et al. 2010; Hong and Xu 2011), and about half of the species occurs in the Oriental Region. However, there still exists some taxonomic confusion because of different concepts among different authors.

At present about 20 species of Stephanidae belonging to five genera are known from 6 provinces (Shaanxi, Henan, Hubei, Yunnan, Fujian and Taiwan) of China (Aguiar 2004; van Achterberg and Yang 2004; Hong et al. 2010; Hong and Xu 2011) (Table 1). However, the actual number of Stephanidae occurring in China may be considerably higher, since these wasps exist without doubt in more than the reported regions from China. Here, we describe six new species and synonymize nine species. The genera and species of Stephanidae from China, including the problematic genus *Foenatopus*, are revised, with all species illustrated and keyed.

**Table 1.** List of Species of Stephanidae known from China (before this study)

Species	Distribution
*Foenatopus acutistigmatus* Chao, 1964	Yunnan (Oriental)
*Foenatopus annulitarsus* Enderlein, 1913	Taiwan (Oriental)
*Foenatopus aratifrons* Enderlein, 1913	Taiwan (Oriental)
*Foenatopus cervivulatus* (Chao, 1964)	Yunnan (Oriental)
*Foenatopus chaoi* Belokobylskij, 1995	Yunnan (Oriental)
*Foenatopus chinensis* (Elliott, 1919)	Yunnan (Oriental)
*Foenatopus cinctus* (Matsumura, 1912)	Taiwan (Oriental)
*Foenatopus flavidentatus* (Enderlein, 1913)	Taiwan (Oriental)
*Foenatopus formosanus* Enderlein, 1913	Taiwan (Oriental)
*Foenatopus menglongensis* (Chao, 1964)	Yunnan (Oriental)
*Foenatopus ruficollis* (Enderlein, 1913)	Taiwan (Oriental)
*Foenatopus yunnanensis* Chao, 1964	Yunnan (Oriental)
*Megischus aplicatus* Hong, van Achterberg & Xu, 2010	Hubei (Palearctic)
*Megischus chaoi* van Achterberg & Yang, 2004	Fujian (Oriental)
*Megischus ptosimae* Chao, 1964	Fujian(Oriental), Shaanxi (Palearctic)
*Parastephanellus austrochinensis* Belokobylskij, 1995	Yunnan (Oriental)
*Parastephanellus brevistigma* Enderlein, 1913	Taiwan (Oriental)
*Schlettererius determinatorius* Madl, 1991	Shaanxi (Palearctic)
*Stephanus bidentatus* van Achterberg & Yang, 2004	Henan (Palearctic)
*Stephanus tridentatus* van Achterberg & Yang, 2004	Henan, Shaanxi (Palearctic)

## Material and methods

The studied Stephanidae from China are deposited in the Parasitic Hymenoptera Collection of South China Agricultural University, Guangzhou (SCAU), the Parasitic Hymenoptera Collection of Zhejiang University, Hangzhou (ZJUH), the Netherlands Center for Biodiversity Naturalis, Leiden (RMNH) and Hungarian Natural History Museum, Budapest (HNHM). These specimens originated from both Oriental and Palaearctic China: Shaanxi, Henan, Zhejiang, Hubei, Fujian, Sichuan, Yunnan, Guangxi, Guangdong, Hong Kong, Taiwan and Hainan.

The types examined in this paper are deposited in Institute of Zoology, Chinese Academy of Sciences, Beijing, China (CAZB), the Insect Museum of Chinese Academy of Forestry, Beijing, China (CAFB), the Natural History Museum, London, United Kingdom (BMNH), Senckenberg Deutsches Entomologisches Institut, Müncheberg, Germany (SDEI), The Netherlands Centre for Biodiversity Naturalis, Leiden (RMNH) and Hungarian Natural History Museum, Budapest (HNHM).

**Figure 1.**
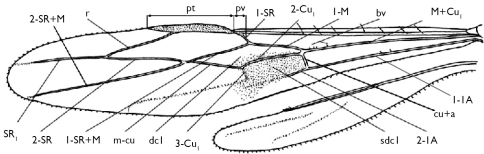
Fore and hind wings of Stephanidae. **pt**=pterostigma; **pv**=parastigmal vein; **bv**=spiny setae or bristles near apex of vein M+CU1; **dc1**=first discal cell; **sdc1**=first subdiscal cell (van Achterberg 2002).

Morphological terminology, including the wing venation system (Fig. 1), follows van Achterberg (2002). Observations and descriptions were made either under an Olympus SZ61 stereoscope, or a Shunyu SZ45-ST1 stereoscope, in combination with a 40W LED lamp. Photographic images were processed with both Image-Pro Plus and AnalySIS Extended Focal Imaging software, and figures were finished with Adobe Photoshop® 8.0.1 and ACDSee 10.0, mostly to adjust the size and background.

## Systematics

### Family Stephanidae

Leach 1815

http://species-id.net/wiki/Stephanidae

Stephanida Leach 1815: 142.
Stephanidae : Haliday 1839.

#### Diagnosis. 

Body slender and richly sculptured; head with five “coronal teeth” around front ocellus; somewhat subspherical; flagellum with 23–40 flagellomeres; clypeus small and protruding; labrum strongly protruding; frons and temple often with ivory streaks in some genera; pronotum modified; posterior pronotum partly covering mesoscutum anteriorly; hind leg highly modified, hind coxa often transversely costate or striate; basal half of hind tibia more or less compressed; inner side of hind tibia usually with a submedial impression; hind femur more or less swollen and dentate, usually with 2 or 3 large teeth and several small ones ventrally; first metasomal tergite more or less elongate and cylindrical; females possessing a triangular or reversed U/V-shaped pygidial impression; ovipositor sheath very long and often with ivory or whitish subapical band in some genera (van Achterberg 2002; Aguiar 2006).

#### Distribution.

Cosmopolitan, but mainly restricted to tropical and subtropical areas (van Achterberg 2002).

#### Notes.

The name is derived from the Greek word “Stephanos”, meaning “corona” or “crown”, in reference to the five corona-like tubercles around the anterior ocellus.

Before this study, 345 extant species in 13 genera in the family Stephanidae were known worldwide (van Achterberg and Yang 2004; van Achterberg and Quicke 2006; Aguiar 2004, 2006; Aguiar and Jennings 2005, 2010; Aguiar et al. 2010; Hong et al. 2010; Hong and Xu 2011), of which 20 species in 5 genera were known from China (Aguiar 2004; van Achterberg and Yang 2004; Hong et al. 2010; Hong and Xu 2011). In this paper, 6 new species are described and 9 species synonymized; altogether 21 species of Stephanidae are reported from 12 provinces or regions in China. A key to genera of Stephanidae from China and adjacent regions is as follows:

#### Key to genera of Stephanidae possibly occurring in China and adjacent regions

(modified after van Achterberg 2002)

**Table:** 

1	Sternite of first metasomal tergite differentiated from its tergite (Figs 363, 375), and first tergite 1.9–4.6 times as long as its apical width, not cylindrical, about as long as second tergite (Figs 362, 363, 373, 375); second tergite sessile and smooth basally (Figs 362, 375); vein cu-a of hind wing present as pigmented vein (Fig. 365); hind coxa with small subapical dorsal tooth (Figs 358, 359, 372); hind tarsus of female with five tarsomeres (Fig. 360); vein 1-M of fore wing distinctly curved (Figs 351, 365); hind tibia not distinctly narrowed and compressed basally (Figs 361, 374); posterior pronotum more or less rectangularly connected with rest of pronotum (Figs 352, 366); eighth metasomal tergite of female with apical protuberance (“pygidial process”; Figs 362, 363, 373, 375); Subfamily Schlettereriinae Belokobylskij, 1995	*Schlettererius* Ashmead, 1900
–	Sternite of first tergite not differentiated from its tergite (Figs 157, 385, 396), and first tergite 4.2–17.6 times as long as its apical width, cylindrical, distinctly longer than second tergite; second tergite more or less petiolate and sculptured basally (Figs 72, 120, 274, 321, 334, 347); hind wing without trace of vein cu-a; hind coxa without dorsal tooth; hind tarsus of female nearly always with three tarsomeres (but with five tarsomeres in the genus *Stephanus*); vein 1-M of fore wing straight or nearly so (Figs 58, 140, 240, 252, 376); hind tibia distinctly narrowed and compressed basally (Figs 91, 143, 248, 262, 383); posterior pronotum gradually merging in remainder of pronotum (Figs 59, 241, 253, 377); eighth metasomal tergite of female usually without apical protuberance (“pygidial process”) (Figs 72, 120, 261; but present in *Pseudomegischus*); Subfamily Stephaninae Leach, 1815 (including Subfamily Foenatopodinae Elliott, 1922)	2
2	First subdiscal cell of fore wing comparatively wide basally, wider than first discal cell (Figs 252, 265, 325) or vein 1-SR of fore wing not differentiated and first discal cell absent because of absence of vein 1-SR+M (Figs 58, 89, 140, 174); hind tibia usually hardly longer than hind femur; temple often with pale yellowish streak along eye (Figs 56, 75, 123, 257, 330); inner side of hind tibia with a long oblique depression (Figs 24, 96, 119, 138, 262)	3
–	First subdiscal cell of fore wing comparatively narrow basally, about as wide as first discal cell or narrower (Figs 228, 239, 240, 376, 387) and vein 1-SR of fore wing differentiated with first discal cell present because of presence of vein 1-SR+M (Figs 228, 239, 240, 376, 387); hind tibia usually distinctly longer than hind femur (Figs 251, 383, 394); temple often with pale patch ventrally and usually without a pale yellowish streak along eye (Figs 245, 381, 392); inner side of hind tibia variable, frequently without a long oblique depression (Fig. 249)	4
3	Veins 2-SR and 2-SR+M of fore wing absent (Figs 49, 58, 125, 174); vein 1-SR of fore wing not or hardly differentiated because of absence of vein 1-SR+M; sclerite present between hind coxae; neck moderately to very slender and finely striate and no distinct pronotal fold, rarely with weakly developed pronotal fold and specialised (Fig. 50, 59, 68, 116, 141); outer side of hind tibia posteriorly usually without fine oblique striae and/or ventrally with fine carina; vein 2-CU1 of fore wing nearly always reduced (Figs 49, 58, 67, 115, 125, 158), but sometimes complete (Figs 2, 12, 26, 40, 89); metapleuron slender; ovipositor sheath always with ivory subapical band (Figs 8, 62, 73, 121, 144, 153)	*Foenatopus* Smith, 1861
–	Veins 2-SR and 2-SR+M of fore wing present (Figs 252, 265, 278, 287, 325), sometimes only pigmented; vein 1-SR of fore wing distinctly differentiated because of presence of vein 1-SR+M; sclerite absent between hind coxae; neck short and comparatively robust, without pronotal fold or transverse carinae (Figs 253, 300, 326, 339); outer side of hind tibia with distinct oblique striae ventrally (but often fine or only ventrally distinctly developed and frequently with some rugulosity and/or apical half of tibia with ventral carina more or less developed), rarely without striae or ventral carina; vein 2-CU1 of fore wing completely developed (Figs 252, 265, 278, 287, 325); metapleuron robust; ovipositor sheath without ivory subapical band (Figs 264, 277, 311, 324, 337, 350)	*Parastephanellus* Enderlein, 1906
4	Hind tarsus of female with five tarsomeres; hind femur (Figs 383, 394) distinctly slender and elongate, coarsely striate, ventrally with 2–3 large teeth, rarely with 4 teeth; inner side of hind tibia only with a short narrow oblique groove below a small convexity (Figs 383, 394)	*Stephanus* Jurine (in Panzer), 1801
–	Hind tarsus of female with three tarsomeres; hind femur (Figs 238, 247) comparatively robust and less elongate, largely smooth and with some punctures, ventrally with 2 large teeth; inner side of hind tibia usually with wide submedial depression, occupying whole width of tibia or depression absent	5
5	Ovipositor sheath without ivory subapical band; pronotum with weak or strong transverse protuberance; temple with pale yellowish streak along eye; hind tibia with a ventral carina and/or with oblique striae ventro-posteriorly	*Pseudomegischus* van Achterberg, 2002
–	Ovipositor sheath with ivory subapical band (Fig. 250); pronotum without transverse protuberance; temple without pale yellowish streak along eye, at most with a ventral patch (Figs 227, 245); outer side of hind tibia without oblique striae or rugulosity and ventrally evenly rounded (Fig. 249)	*Megischus* Brullé, 1846

#### Genus Foenatopus

Smith, 1861

http://species-id.net/wiki/Foenatopus

Figs 2 [Fig F3] [Fig F4] [Fig F5] [Fig F6] [Fig F7] [Fig F8] [Fig F9] [Fig F10] [Fig F11] [Fig F12] [Fig F13] [Fig F14] [Fig F15] [Fig F16] [Fig F17] [Fig F18] [Fig F19] [Fig F20] [Fig F21] [Fig F22] [Fig F23] [Fig F24] [Fig F25] 220

Foenatopus Smith 1861: 58. Type species (by monotypy): *Stephanus indicus* Westwood, 1841.
Diastephanus Enderlein 1905: 473. Type species: *Stephanus flavomaculatus* Enderlein, 1901. Synonymized by Benoit, 1956.
Neostephanus Kieffer 1904: 1–4. Type species (by monotypy): *Neostephanus alluaudi* Kieffer, 1904. Synonymized by Narendran et al. (2001).

##### Diagnosis.

Temple often with pale yellowish streak along eye (Figs 56, 65, 75, 85, 123, 132); neck emarginate anteriorly, moderately to very slender, finely striate without distinct pronotal fold basally (Figs 3, 14, 41, 50, 59, 78, 141); posterior pronotum gradually merging into remainder of pronotum; metapleuron slender; vein 2-CU1 of fore wing nearly always reduced (Figs 49, 58, 67, 115, 125, 158), sometimes complete (Figs 2, 12, 26, 40, 89); veins 2-SR and 2-SR+M of fore wing absent (Figs 49, 58, 125, 174); vein 1-SR of fore wing not or hardly differentiated because of absence of vein 1-SR+M; hind wing without trace of vein cu-a; inner side of hind tibia with a long oblique depression; hind tibia distinctly narrowed and compressed basally (Figs 24, 44, 53, 100, 110, 119, 138, 187); hind tarsus of female with three tarsomeres; eighth metasomal tergite of female usually without apical protuberance (Figs 7, 22, 45, 72, 120, 139, 163, 216); ovipositor sheath always with ivory or yellowish subapical band (Figs 8, 62, 73, 121, 144, 153).

##### Distribution.

Afrotropical, Neotropical, Palaearctic and Oriental.

##### Notes.

Before this study, 159 species in the genus *Foenatopus* were known worldwide, of which 12 species were known from China. In this paper, 3 species of this genus are new to science and 8 species are synonymized; altogether 10 species are known from China. A key to species of the genus *Foenatopus* from China and adjacent regions follows:

##### Key to species of the genus *Foenatopus* from China and adjacent regions

**Table:** 

1	Pterostigma comparatively short and wide, apically obtuse (Figs 12, 26, 40, 158, 183, 192, 201); vertex coarsely irregularly rugose (Figs 17, 32, 46, 165, 189, 198, 208); head somewhat transverse in dorsal view; ovipositor sheath with brownish subapical band (Figs 25, 31, 164) or completely black (Figs 35, 196, 207)	2
–	Pterostigma long and narrower, more or less subparallel-sided, apically acute (Figs 2, 49, 58, 67, 77, 89, 97, 106, 115, 125, 140, 149, 174, 211); vertex transversely striate or carinate (Figs 9, 25, 64, 74, 84, 92, 102, 112, 122, 131, 146, 154, 180, 218); head more or less globose in dorsal view; ovipositor sheath with ivory or whitish subapical band (Figs 8, 62, 73, 90, 101, 111, 121, 130, 144, 153) or completely black (Figs 54, 178)	5
2	Third metasomal tergite with two large ivory patches (Fig. 163); scutellum medially distinctly convex (Fig. 161); propodeum rather matt, anterior two thirds largely and superficially granulate, posterior third foveolate-rugose (Fig. 161); [both large teeth on hind femur whitish (Fig. 162); ovipositor sheath (Fig. 164) about 0.7 times as long as body and with brownish subapical band 0.6 times as long as blackish apical part]	*Foenatopus maculiferus* sp. n.
–	Third tergite entirely dark brown or blackish (Figs 22, 45); scutellum medially flat (Figs 16, 29, 43, 186, 204); propodeum shiny and largely foveolate (Figs 16, 29, 43, 186, 204), but sparsely so in *Foenatopus menglongensis* (Fig. 173)	3
3	Propodeum sparsely and superficially foveolate (Fig. 173); hind femur more swollen (Fig. 171); ovipositor sheath about 0.7 times as long as body; posterior half of pronotum dorsally finely superficially reticulate (Figs 169, 170; cf. Figs 159, 160 of *Foenatopus maculiferus*); [hind femur with two large teeth ventrally and central large teeth whitish; vein 2-CU1 of fore wing nearly absent (Fig. 168)]	*Foenatopus menglongensis* (Chao, 1964) Notes. Ovipositor sheath of holotype of F. menglongensisbroken off but according to the original description largely dark brown and apically blackish. If ovipositor about 0.3 times of body length and brown without a subapical band or sheath brownish subapically cf. *Foenatopus burmaensis* (Narendran & Sureshan, 2003)from Burma.
–	Propodeum mostly distinctly foveolate (Figs 16, 29, 43, 186, 204); hind femur comparatively elongate (Figs 23, 30, 44, 187, 195, 206); ovipositor sheath 0.9–1.1 times as long as body; posterior half of pronotum dorsally often rugose and rugulose (Figs 14, 27, 41, 184, 193, 202)	4
4	Vein 2-CU1 of fore wing long, 0.7–1.1 times as long as vein cu-a (Figs 12, 26, 37, 40); head in lateral view more or less elliptical (Figs 18, 33, 47); frons of female irregularly vermiculate-rugose (Figs 19, 34); vein r of fore wing obtusely angled with vein SR1 because of short sublongitudinal vein 3-SR (Figs 12, 26, 40); neck medially distinctly impressed (Figs 13, 27, 39, 41); hind basitarsus of female largely ivory or whitish (Figs 23); ovipositor sheath with small subapical brownish band (Figs 25, 31) or completely black (Fig. 35)	*Foenatopus annulitarsus* Enderlein, 1913
–	Vein 2-CU1 of fore wing short or absent, up to 0.2 times as long as vein cu-a (Figs 183, 192, 201); head in lateral view globose (Figs 190, 199, 209); frons of female regularly transversely rugose (Figs 191, 200, 210); vein r of fore wing acutely angled with vein SR1 (Figs 183, 192, 201), rarely intermediate; neck medially comparatively less impressed (Figs 184, 193, 202); hind basitarsus of female brownish (Fig. 195); ovipositor sheath nearly completely brownish or blackish, without different coloured subapically band (Figs 188, 196, 207); [habitus largely orange or yellowish brown, but dark brown or black in *Foenatopus trilobatus*; sculpture on pronotum weaker, but distinct and coarser in *Foenatopus trilobatus*]	*Foenatopus ruficollis* (Enderlein, 1913)
5	Pterostigma comparatively obtuse apically (Fig. 211); border of yellowish streak of temple diffuse posteriorly (Fig. 219); body largely dull	*Foenatopus yangi* sp. n. Notes. If pronotum only finely reticulate, without distinct transverse carinae or rugae, cf.F. chinnarensis (Sureshan, 1999) from Kerala, India. The female of this species is not known with certainty. The female described by Sheela and Ghosh (2009) from Arunachal Pradesh (formerly Assam), India, is probably not conspecific because of differences in colour (e.g. has hind femur (except reddish-brown base but including teeth) black and sculpture (e.g. three carinae between posterior ocelli).
–	Pterostigma distinctly acute apically (Figs 49, 58, 67, 77, 89, 97, 106, 115, 125, 140, 149, 174); if intermediate, then border of yellowish streak of temple well defined posteriorly; body largely shiny	6
6	Vein r of fore wing strongly oblique and gradually merging into vein 3-SR+SR1 (Figs 2, 89, 97, 106); vein 2-CU1 of fore wing present, sclerotized part 0.3–1.2 times as long as vein cu-a (Figs 2, 89, 97, 106)	7
–	Vein r of fore wing moderately oblique and distinctly angled with vein 3-SR+SR1 (Figs 49, 58, 115, 125, 140, 149); vein 2-CU1 of fore wing absent or nearly so (Figs 49, 58, 115, 125, 140, 149)	8
7	Foveolae of propodeum and interspaces between foveolae smooth (Fig. 5); pronotum densely carinate (Fig. 3); frons of female largely reddish brown (Fig. 11); [vertex distinctly carinate, more coarsely near ocelli (Fig. 9); temple smooth and shiny (Fig. 10); frons transversely carinate-rugose; neck with complete transverse carinae; ovipositor sheath about 1.3 times as long as body; subapical whitish band of ovipositor sheath 1.4–1.8 times as long as apical blackish part]	*Foenatopus acutistigmatus* Chao, 1964 Notes. If ovipositor sheath is about 1.5 times as long as body, cf. F. longicauda Elliott, 1919, from Nilgiri Hills, India. If body mainly reddish brown, vertex finely transversely rugose, temple with wide pale stripe ventrally and ovipositor about as long as body, cf. F. punctatus Elliott, 1919, from Burma. If frons and vertex coarsely striate and the propodeum less closely foveolate, cf. F. longicollis (Cameron), from Sarawak, Malaysia. If ovipositor sheath evenly blackish, body dark brown, ovipositor sheath about 0.7 times body and temple angulate in dorsal view, cf. F. jodhpurensis Narendran, 2001, from western India. If pronotum completely transversely carinate, carinae of neck interrupted medially; temple smooth and shiny, with pale yellowish stripe narrowly along eye; ovipositor sheath unknown, but has ovipositor about 1.3 times as long as body cf. F. similicus Narendran, 2001, from northern India.
–	Foveolae of propodeum and interspaces between foveolae coriaceous (Figs 94, 109); pronotum less densely carinate (Figs 93, 107); frons of female with distinct yellowish stripes (Figs 88, 104, 114); [body varies from black to nearly completely brown; especially small males have no vein 2-CU1 of fore wing]	*Foenatopus cinctus* (Matsumura, 1912)
8	Middle pale stripe of frons comparatively wide dorsally (Figs 124, 133, 148, 156; both sexes, but sometimes less in male) and base of anterior tooth of corona yellowish brown; pronotum often yellowish brown or dark brown posteriorly and usually contrasting with black mesoscutum (Figs 127, 151); teeth of hind femur completely to partly pale yellowish or ivory (Figs 129, 143, 152); vein r of fore wing somewhat less oblique (Figs 125, 140, 149); [ovipositor sheath with long ivory subapical band (Figs 121, 130, 144, 153)]	*Foenatopus flavidentatus* (Enderlein, 1913)
–	Middle pale stripe of frons absent or narrow dorsally (Figs 66, 76) and base of anterior tooth of corona dark brown; pronotum black posteriorly and as dark as mesoscutum (Figs 59, 78); teeth of hind femur often completely or largely black or dark brown (Figs 61, 71); vein r of fore wing slightly more oblique (Figs 58, 67)	9
9	Pronotum robust in dorsal view and its posterior half distinctly striate or carinate (Figs 175, 176); face of female without distinct pale lateral stripes (Fig. 182); frons comparatively coarsely sculptured (Fig. 182); [ovipositor sheath without subapical ivory band (Fig. 178)]	*Foenatopus quadridens* (Elliott, 1920)
–	Pronotum slender in dorsal view and its posterior half mainly reticulate-coriaceous, at most with some short striae or carinae (Figs 50, 59, 68, 78); face of female with distinct pale lateral stripes (Figs 66, 76) or triangular patches (Fig. 57); frons comparatively finely sculptured (Figs 57, 66, 76, 86)	10
10	Ovipositor sheath completely black (Fig. 54); frons of female with triangular pale yellowish or ivory patches laterally (Fig. 57); anterior half of pronotum (“neck”) in lateral view without transverse carinae and flat medially or slightly impressed (Fig. 51); frons of male partly dark brown and with 3 ivory stripes	*Foenatopus brevimaculatus* sp. n. Notes. If propodeum sparsely foveolate and with large coriaceous interspaces (Fig. 7 in Elliott 1920), pronotum anteriorly with transverse striae, scutellum coarsely punctate laterally, head reddish and mesosoma black, cf. *Foenatopus sulcatus* (Elliott, 1920) from Laos [type not found in BMNH, probably lost?].
–	Ovipositor sheath with ivory subapical band (Figs 62, 73); frons of female with elongate pale yellowish or ivory patches laterally (Figs 66, 76); anterior half of pronotum (“neck”) in lateral view with transverse carinae (Figs 60, 69, 79) and depressed medially (Figs 59, 68, 78); frons of male completely ivory (Fig. 86)	*Foenatopus chinensis* (Elliott, 1919)

##### Foenatopus acutistigmatus

Chao, 1964

http://species-id.net/wiki/Foenatopus_acutistigmatus

Figs 2–11

Foenatopus acutistigmatus Chao 1964: 381–383, 389; Belokobylskij 1995: 19; Aguiar 2004: 18.

###### Type material.

Holotype, ♀ (CAZB), “CHINA: Yunnan, Xishuangbanna, Meng’a, 1050–1080 m., Chinese Academy of Sciences (C.A.S.)”, “11.v.1958, Shu-yong Wang”, “HOLOTYPE”, “*Foenatopus acutistigmatus* Chao, Holotype”. Paratypes (2 ♀; CAZB): 1 ♀, id., but “20.v.1958”, “PARATYPE”, “*Foenatopus acutistigmatus* Chao, Paratype”; 1 ♀, id., but “11.x.1958, Zhi-zi Chen”, “PARATYPE”, “*Foenatopus acutistigmatus* Chao, Paratype”.

###### Other material.

1 ♀ (SCAU): CHINA: Guangdong, Mt. Nanling, 16.vi.2009, Wang Zi-chen, No. 200800190.

###### Diagnosis.

Vertex distinctly carinate, more coarsely near ocelli (Fig. 9); frons largely reddish brown, transversely carinate-rugose (Fig. 11); temple completely yellowish, smooth and shiny (Fig. 10), in dorsal view distinctly angulate (Fig. 9); pronotum with comparatively long parallel-sided anterior part, neck dorsally with complete transverse carinae (Fig. 3), laterally distinctly carinate or striate (Fig. 4); propodeum densely foveolate, foveolae and interspaces in between smooth (Fig. 5); pterostigma long and its apex distinctly acute; vein 2-CU1 of fore wing well developed, 0.7–0.9 times as long as vein cu-a (Fig. 2); hind femur ventrally with 2 large teeth, basally with 3–4 tubercles (Fig. 6); ovipositor sheath about 1.3 times as long as body, subapical whitish band 1.4–1.8 times as long as apical blackish part.

###### Description.

Redescribed after a female from Guangdong (Mt. Nanling), length of body 22.8 mm, of fore wing 11.6 mm, and of ovipositor sheath 28.9 mm.

*Head.* Flagellum with 38 flagellomeres; first flagellomere 3.9 times as long as wide, and 0.8 times as long as second flagellomere; anterior coronal teeth acute and moderately large, both posterior ones arcuate and smaller; frons coarsely transversely carinate-rugose, posteriorly both ends of carinae curved backwards to coronal area (Fig. 11); vertex with 4 strong and relatively short transverse carinae between posterior ocelli, followed by transversely carinate flattened area, carinae antero-medially coarse and strong, more or less rugose, posteriorly much finer and straight, reaching to part of gena (Fig. 9); temple smooth and shiny, angulate in dorsal view (Fig. 10).

*Mesosoma.* Neck (Figs 3, 4) moderately elongate, dorsally with complete transverse carinae, laterally distinctly carinate or striate, anteriorly distinctly deeply emarginate, medio-dorsally slightly concave; middle pronotum at lower level than posterior pronotum, superficially striate and punctate dorsally, laterally somewhat microreticulate; posterior pronotum anteriorly striate, posteriorly smooth and convex laterally; propleuron coriaceous and setose; prosternum densely striate, posteriorly with irregular small foveolae; mesoscutum anterior third striate, posteriorly coarsely foveolate-rugose; axillae largely coarsely rugose, medio-dorsally separated a large, deep fovea; scutellum largely smooth and shiny medially, and with a few foveolae laterally (Fig. 5); mesopleuron robust, dorsal flat part smooth, antero-ventrally rugose and with pubescence, remainder of ventral part largely striate and with some punctures; metapleuron ventrally crenulate, convex part of metapleuron and propodeum (Fig. 5) densely foveolate.

*Wings.* Fore wing (Fig. 2): wing membrane hyaline; vein 2-CU1 distinctly developed and 0.9 times as long as vein cu-a; vein cu-a distinctly curved; pterostigma moderately elongate and apically acute, 1.6 times as long as vein r and 12 times longer than its maximum width; vein r ends third length of pterostigma behind level of apex of pterostigma; vein SR1 subparallel to costal margin, vein SR1 and vein r obtuse-angled.

*Legs.* Hind coxa slender, shiny, anterior part rugose, remainder annular, finely transversely spaced striate; hind femur (Fig. 6) moderately slender, densely finely striate, apically somewhat coriaceous, ventrally with 2 large acute teeth and some denticles in between, teeth antero-ventrally distinct and triangular; basal narrow part of hind tibia coriaceous, 1.2 times as long as widened part, and with ventral carina, outer side of widened part of hind tibia coriaceous, inner side basally distinctly depressed, apically with densely bristly setose area; hind basitarsus slender, parallel-sided, its ventral length 6.8 times as long as its width.

*Metasoma.* First tergite very slender, cylindrical, densely finely transversely striate (Fig. 7), 17.6 times as long as its width and 2.1 times as second tergite; remainder slender, smooth and with a few sparse, short setae; pygidial area (Fig. 7) triangular, shallowly impressed laterally, somewhat granulate, and narrowly lamelliform posteriorly; subapical whitish band of ovipositor sheath 1.44 times as long as apical blackish part; length of ovipositor sheath 1.3 times as long as body.

*Colour.* Black, except parts as follows: frons largely reddish; temple with a wide yellowish streak; tibiae and tarsi of legs brown; wing membrane hyaline; subapical part of ovipositor sheath whitish.

*Male.* Very similar to female in structure and colour, but differs in size; body length up to 13 mm according to Chao (1964).

*Variation.* Female: length of body 18–23 mm, of fore wing 10–12 mm, and of ovipositor 23–29 mm; vein 2-CU1 0.7–0.9 times as long as vein cu-a, pterostigma 1.6–1.9 times as long as vein r and 12–16 times longer than its maximum width; first tergite 17.6–19.7 times as long as its maximum width and 2.1–4.2 times as second tergite; subapical whitish band of ovipositor sheath 1.4–1.8 times as long as apical blackish part.

###### Distribution.

Oriental China (Yunnan, Guangdong).

##### Foenatopus annulitarsus

Enderlein, 1913

http://species-id.net/wiki/Foenatopus_annulitarsus

Figs 12 [Fig F4] [Fig F5] [Fig F6] 48

Foenatopus annulitarsus Enderlein 1913: 206, 211; Belokobylskij, 1995: 21; Huflejt, 1996: 97; Aguiar, 2004: 19.
Foenatopus aratifrons Enderlein 1913: 207, 211; Belokobylskij 1995: 21; Aguiar 2004: 19. syn. n.
Foenatopus annulitarsis [sic!]: Elliott 1922: 782, 787; Chao 1964: 381, 387.
Foenatopus yunnanensis Chao 1964: 381–382, 388–389, Figs I (3–4), II (7–8), III (4), IV (8), V (5, 11–12); Belokobylskij 1995: 19; Aguiar 2004: 39.syn. n.

###### Type material.

Lectotype of *Foenatopus annulitarsus*, ♀ (SDEI): “[CHINA], Formosa, Taihorin, H. Sauter, 1911”, “7. VIII.”, “*Foenatopus annulitarsus* TypeEnderlein. ♀ Dr. Enderlein det. 1913”, “Lectotypus *Foenatopus annulitarsus* Enderlein, des. Belokobylskij 92”, “OSUC 0021932”. Paralectotype, 1 ♀ (SDEI): “Formosa, Hoozan, H. Sauter, 1910”, “7. IX.”, “*Foenatopus annulitarsus* TypeEnderlein. ♀ Dr. Enderlein det. 1913”, “Paralectotypus *Foenatopus annulitarsus* Enderlein des. Belokobylskij 92”, “Eberswalde coll. DEI”, “OSUC 0021933”.

Holotype of *Foenatopus aratifrons*,♂ (SDEI): “[China], Formosa, Kankau (Koshun), H. Sauter, v. 1912”, “*Foenatopus aratifrons* Type Enderl. ♂ Dr. Enderlein det. 1913”, “ Holotypus” “*aratifrons* Enderl. 1913 HT *aratifrons*”, “Coll. DIE Eberswalde”, “OSUC 0021934”.

Holotype of *Foenatopus yunnanensis*, ♀, “CHINA: Yunnan, Xishuangbanna, Mengzhe, 1200 m., C.A.S.”, “19.vii.1958, Yong-shu Wang”, “HOLOTYPE”, “*Foenatopus yunnanensis* Chao, Holotype”. Paratypes, 2 ♀ (CAZB): 1 ♀, “CHINA: Yunnan, Xishuangbanna, Meng’a, 1050–1080 m. C.A.S.”, “19.vii.1958, Fu-ji Pu”; 1 ♀, id., but “CHINA: Yunnan, Xishuangbanna, Mengzhe, 1200 m. C.A.S.”, “30.viii.1958”, “PARATYPE”, “*Foenatopus yunnanensis* Chao, Paratype”.

###### Other material.

2 ♀ + 1 ♂ (SCAU): 1 ♀, CHINA: Hainan, Mt. Diaoluoshan, 16.vii.2006, Li-qiong Weng, No. 200800171; 1 ♀, CHINA: Hainan, 3.ii.1981, Li-zhong Hua, No. 870229; 1 ♂, CHINA: Guangxi, Longzhou, Nonggang, 18.v.1982, Jun-hua He, No. 821489; 1 ♀ (RMNH): “N. Vietnam: Hoa Binh Pa Co Hang Kia N. R., 1041 m, N20°44'29" E04°55'44",11–23. x. 2009, Mal. tr. 23, RMNH 09 C. v. Achterberg & R. de Vries”; 4 ♀ (HNHM): “Formosa, Sauter”, “Kosempo, ix.1909, id., i.1910”, “Fuhosho, viii.1909, id., ix.1909”.

###### Diagnosis.

Head transverse in dorsal view (Figs 17, 32, 46) and more or less elliptical in lateral view (Figs 18, 33, 47); frons of female irregularly vermiculate-rugose; temple with some striae from vertex; neck short, with several pairs of carinae and medially depressed; pronotum robust and with strongly developed sculptures (Figs 14, 15, 27, 28, 38, 39, 41, 42); propodeum distinctly foveolate and with rugulae in between (Figs 16, 29, 43); pterostigma moderately short and its apex comparatively wide (Figs 12, 26, 37, 40); vein 2-CU1 of fore wing distinctly developed, 0.7–1.1 times as long as vein cu-a (Figs 12, 26, 37, 40); vein r of fore wing obtusely angled with vein SR1 because of short sublongitudinal vein 3-SR; hind femur (Figs 23, 30, 44) with 3 large teeth ventrally and the teeth sometime with whitish part; hind basitarsus of female largely ivory or whitish (Figs 23); length of ovipositor sheath 0.7–0.9 times as long as body length; ovipositor sheath with small brownish subapical band (Figs 25, 31) or completely black (Fig. 35).

###### Description.

Redescribed after a female from Hainan (Mt. Diaoluoshan), length of body 13.1 mm, of fore wing 7.0 mm, and of ovipositor sheath 9.1 mm.

*Head.* Flagellum with 30 flagellomeres; first flagellomere very short, twice its maximum width, almost equal to pedicel and half as long as second flagellomere; frons (Fig. 34) strongly reticulate-rugose, rugae extending to coronal area; three anterior coronal teeth acute, both posterior ones arcuate; vertex (Fig. 32) with four strong, curved spaced carinae followed by coarsely transversely rugose and slightly convex area; temple (Fig. 33) with weak rugae from vertex along orbit, ventrally smooth, temple roundly narrowed behind eyes; head transverse in dorsal view.

*Mesosoma.*Neck (Figs 27, 28) rather robust, anteriorly deeply emarginate, dorso-medially impressed, laterally with four pairs of oblique carinae, carinae interrupted dorsally, neck postero-dorsally at lower level than middle pronotum; middle pronotum coarsely transversely striate, postero-medially with a shallow foveola; posterior pronotum largely rugose, posteriorly somewhat striate, laterally convex part weakly reticulate-rugose; mesoscutum largely strongly carinate-rugose, anterior 0.2 transversely striate; notauli and middle groove distinct on anterior 0.2; axillae strongly foveolate and with striate interspaces; scutellum (Fig. 29) laterally and marginally foveolate and with striate interspaces, medially largely smooth and somewhat longitudinal striate; mesopleuron largely striate, anteriorly covered with short whitish setosity, ventrally sparsely shallowly foveolate; convex part of metapleuron strongly reticulate-foveolate, foveolae large and deeper than those on mesopleuron; propodeum (Fig. 29) mostly with median-sized, circular foveolae and with striate interspaces, inside of foveolae polished, some foveolae posteriorly coalescent and resulting two large foveae.

*Wings.* Fore wing (Fig. 26): wing hyaline; vein 2-CU1 0.9 times as long as vein cu-a; pterostigma obtuse apically and comparatively wide and short, 1.4 times as long as vein r and 8.3 times as its maximum width; vein r ends 0.24 times length of pterostigma behind level of apex of pterostigma; vein SR1 about 1.3 times as long as vein r; vein SR1 and vein r obtuse-angled, vein SR1 elongate towards vein margin and ending near before reaching vein margin.

*Legs.* Anterior 0.6 of hind coxa coarsely rugose and somewhat reticulate, posterior part transversely annularly striate; hind femur (Fig. 30) densely transversely strigate, with 3 large ventral teeth, basal tooth obtuse-triangular; hind tibia 1.2 times as long as hind femur, largely irregularly obliquely strigate, apically densely bristly setose; basal narrow part of hind tibia 1.5 times as widened part, inner side of widened part basally steeply depressed; basitarsus parallel-sided, its ventral length 4.7 times as long as its width, ventrally densely setose.

*Metasoma.* First tergite largely transversely striate, basal 0.2 rugose and apical 0.05 smooth, first tergite 10.2 times as long as its maximum width, 2.8 times as long as second tergite and 0.8 times as long as rest of metasoma; second tergite basal 0.2 rugose, medially largely microreticulate and apical 0.2 aciculate; rest of tergites transversely aciculate, somewhat smooth; pygidial area setose, laterally shallowly impressed, medially distinctly convex and granulate, pygidial impression widely reversed V-shaped; length of ovipositor sheath 0.7 times as long as body length, length of subapical brownish band 0.4 times length of dark apex (Fig. 31).

*Colour.* Largely black, except parts as follows: head dark brown with some reddish tint; frons ivory from mandibles to narrowly above antennae; scape, pedicel, posterior coronal teeth and carinae between posterior ocelli on vertex, two spots behind posterior ocelli reddish brown; basal rugose part dorsally red brown but laterally with two yellow spots; malar space, basal part of mid tibia, basal half of mid basitarsus, ventral large teeth of hind femur and hind basitarsus whitish; subapex of ovipositor sheath pale brown.

*Male.* Body length 16 mm; almost the same as female, but different in body colour (Chao, 1964). A specimen from Guangxi (No. 821489) with body length 11.5 mm, frons completely yellowish (Fig. 36), carinae on the neck complete and transverse (Figs 38, 39), and vein 2-CU1 of fore wing 0.3 times as long as vein cu-a (Fig. 37).

*Variation.* Female: length of body 6.5–15 mm, of fore wing 3.6–7.7 mm, and of ovipositor sheath 7.2–14 mm; vein 2-CU1 0.9–1.1 times as long as vein cu-a; pterostigma 1.4–1.8 times as long as vein r and 8.3–9.5 times as long as its maximum width; first tergite 9.0–12.8 times as long as its maximum width, 2.1–2.8 times as second tergite and 0.7–0.9 times as rest of tergites; length of ovipositor sheath 0.7–0.9 times as long as body length; length of subapical brownish or yellowish band of ovipositor sheath 0.4–0.8 times length of dark apex. One specimen from Hainan (No. 870229) has ovipositor sheath totally dark brown and without pale brownish part; carinae on the neck complete and not interrupted dorsally; mixed coloured, coronal area, pronotum, propodeum, first tergite largely red brown. Male: body length 11.5–16 mm; frons completely yellowish (Figs 36, 48); Neck with transverse and complete carinate (Fig. 39) or obliquely carinate (Fig. 41); vein 2-CU1 of fore wing 0.3–0.9 times as long as vein cu-a.

###### Distribution.

China (Yunnan, Guangxi, Taiwan, Hainan); Vietnam.

###### Notes.

The type series of *Foenatopus yunnanensis* has been examined and proved to be in the variation of *Foenatopus annulitarsus*. Vietnam is a new record for this species.

##### Foenatopus brevimaculatus sp. n.

urn:lsid:zoobank.org:act:55C7F0B1-F0B1-47E1-859A-80BA4FD02AB4

http://species-id.net/wiki/Foenatopus_brevimaculatus

Figs 49–57

###### Type material.

Holotype (SCAU), ♀, CHINA: Hainan, Mt. Yinggeling, 2008.v.1–2, Jing-xian Liu, No. 200800051. Paratypes, 2 ♂ (SCAU), CHINA: Hainan, Mt. Yinggeling, 16.xi.2008, Zai-fu Xu, No. 200800189; CHINA: Guangdong, Mt. Guanyinshan, 15–16.ix.2007, Zai-fu Xu, No. 200800179; 1 ♂ (RMNH), id., but No. 200800180.

###### Diagnosis.

Vertex finely transversely striate (Fig. 55); frons of female with 3 triangular ivory streaks and not reaching level of anterior coronal tooth (Fig. 57); temple smooth and shiny, with a short ivory streak along eye (Fig. 56); neck in lateral view without transverse carinae and flat medially or slightly impressed (Figs 50, 51); pronotum mostly coriaceous; propodeum finely foveolate; pterostigma long and narrow, subparallel-sided, apically acute (Fig. 49); vein 2-CU1 of fore wing absent (Fig. 49); both large teeth on hind femur largely dark brown or blackish (Fig. 53); ovipositor sheath completely black (Fig. 54).

###### Description.

Holotype, female, length of body 12.5 mm, of fore wing 6.7 mm, and of ovipositor sheath 11.8 mm.

*Head.* Antenna with flagellomeres partly missing; frons with spaced striae and microreticulate (Fig. 57); three anterior coronal teeth large and acute, two posterior ones short and wider; coronal area with some longitudinal carinae; vertex flat and finely transversely striate (Fig. 55); temple smooth and shiny, narrowly rounded behind eye (Fig. 56).

*Mesosoma.* Pronotum (Figs 50, 51) slender and mostly coriaceous; neck anteriorly deeply emarginated and flat medial-dorsally; posterior pronotum narrowly smooth; anterior half of mesoscutum transversely striate, posterior half with shallow foveolae; scutellum (Fig. 52) smooth and aciculate, laterally with some small punctures; mesopleuron robust, microreticulate and sparsely punctured, anterior third densely setose; metapleuron and propodeum (Fig. 52) finely foveolate and rugulose in between.

*Wings.* Fore wing (Fig. 7): wing hyaline, vein 2-CU1 absent; pterostigma elongate and subparallel-sided, acute apically, 15 times as long as its maximum width and 2.7 times as vein r; vein r and vein SR1 obtuse-angled, vein r ends 0.2 times length of pterostigma behind level of apex of pterostigma; vein SR1 subparallel to costal margin.

*Legs.* Hind coxa transversely striate, subapex dilated; hind femur (Fig. 53) rugulose or microreticulate, with 2 large ventral teeth and with a comparatively smaller obtuse basal tooth; hind tibia coriaceous, 1.1 times as long as hind femur; basal narrow part of hind tibia 1.1 times as long as widened part, inner side of widened part basally distinctly depressed, followed by convex and setose area, apically densely setose.

*Metasoma.* First tergite transversely striate, 15.3 times as long as its maximum width, 2.5 times as second tergite and 0.9 times as remainder of tergites; second tergite basal 0.2 rugose, remaining tergites largely smooth or weakly aciculate; pygidial area distinctly differentiated, pygidial impression reverse V-shaped; ovipositor sheath completely black (Fig. 54) and 0.9 times as long as body length.

*Colour.*Largely black; frons with 3 short ivory streaks not reaching level of anterior coronal tooth (Fig. 57); hind legs largely dark brown.

*Male.* Similar to female, but differ in: comparatively smaller in size, body length 7–9.5 mm; ivory or yellowish streaks longer and reach level of anterior coronal tooth.

###### Distribution.

Oriental China (Guangdong, Hainan).

###### Etymology.

The species was named *brevimaculatus* because of the short ivory streaks on the frons.

##### Foenatopus chinensis

(Elliott, 1919)

http://species-id.net/wiki/Foenatopus_chinensis

Figs 58 [Fig F10] 86

Diastephanus chinensis Elliott 1919: 73; Elliott 1922: 801, 814; Chao 1964: 383, 388.
Foenatopus chinensis Aguiar 2004: 22.
Diastephanus cerviculatus Chao 1964: 383–384, 388, Figs I (7), III (2–3), IV (1, 3, 11). syn. n.*Foenatopus cerviculatus*Belokobylskij 1995: 21; Aguiar 2004: 22.*Diastephanus flavifrons*Chao 1964: 383–385, 389, Figs I (8); syn. n.
Foenatopus flavifrons Belokobylskij 1995: 21.
Foenatopus chaoi Belokobylskij 1995: 21(new name for *Foenatopus flavifrons* (Chao, 1964), not Elliott, 1917); Aguiar 2004: 22. syn. n.

###### Type material.

Holotype of *Foenatopus chinensis* (BMNH): “Type”, “B.M. Type Hym., 3.a.45”, “B.M. Type *Diastephanus chinensis* Elliott 1919)”, “*Diastephanus chinensis* E.A. Elliott, Type, 5.3.19 [= 5.iii.1919]”, “[?CHINA: Yunnan], Haute Mekong, Tong King, 13.iv.1918, R.V. de Salvaza”, “Indo China, R.V. de Salvaza, 1919–25”.

Holotype of *Foenatopus cerviculatus*, ♀ (CAZB), “CHINA: Yunnan, Xishuangbanna, Yunjinghong, 650 m. C.A.S.”, “8.x.1957, Ling-chao Zang”, “HOLOTYPE”, “*Diastephanus cerviculatus* Chao, Holotype”. Paratypes (2 ♀; CAZB): 1 ♀, “CHINA: Yunnan, Xishuangbanna, Meng’a, 1050–1080 m. C.A.S.”, “13.v.1958, Fu-ji Pu”, “PARATYPE”, “*Diastephanus cerviculatus* Chao, paratype”; 1 ♀, “CHINA: Yunnan, Xishuangbanna, Xiaomengyang, 850 m. C.A.S.”, “28.viii.1958, Yi-ran Zhang”, “PARATYPE”, “*Diastephanus cerviculatus* Chao, paratype”.

Holotype of *Foenatopus chaoi*, ♂ (CAZB): “CHINA: Yunnan, Xishuangbanna, Menghun, 1750 m. C.A.S.”, “1.vi.1958, Zheng Le-yi”, “HOLOTYPE”, “*Diastephanus flavifrons* Chao, Holotype”. Paratypes (2 ♀ (?), CAZB): 1 ♀ , id., but Damenglong, 650 m, 18.vi.1958; 1 ♀, id., but Mengzhe, 670m, 6.ix.1958, Wang Yong-shu, “PARATYPE”, “*Diastephanus flavifrons* Chao, Paratype”.

###### Other material.

3 ♀ + 6 ♂ (SCAU): 1 ♀ + 1 ♂, CHINA: Guangdong, Guangzhou, Shaojiwo, 3.v.2009, Ming-yi Tian, No. 200800185; No. 200800186; 2 ♀, CHINA: Guangdong, Mt. Nankunshan, 1.vi.2009, Zai-fu Xu, No. 200800187; No. 200800188; 1♂, CHINA: Guangdong, Mt. Nankun, x.2009, Chun-dan Hong, No. 200800191; 5 ♂, CHINA: Guangdong, Zhaoqing, Xiwanggu, 14–15.iv.2007, Zai-fu Xu, No. 200800173; No. 200800174; No. 200800175; No. 200800176; CHINA: Guangdong, Mt. Nankunshan, x.2009, Chun-dan Hong, No. 200800191; 3 ♀ (ZJUC): CHINA: Guangxi, Nanning, 1982.v.11, Jun-hua He, No. 821224; id., but No. 8212248; CHINA: Guangxi, Nanning, 1982.v.11, Jun-hua He, No. 821228.

###### Diagnosis.

Vertex finely transversely striate (Figs 64, 74, 84); frons of female with elongate pale yellowish or ivory patches laterally (Figs 66, 76), of male completely ivory (Fig. 86); temple smooth and shiny, with yellowish streak along eye (Figs 65, 75, 85); pronotum long and slender, neck medially distinctly impressed and with several carinae (Figs 59, 68, 78); propodeum distinctly foveolate (Figs 70, 80); vein 2-CU1 of fore wing nearly absent (Figs 58, 67, 77); pterostigma of female distinctly long and apically acute, its length 20–21 times as long as maximum width (Figs 58, 67); hind femur with two large ventral teeth, both large teeth on hind femur mostly dark brown or blackish (Figs 61, 71, 82); pygidial impression of female deep and reverse V-shaped (Fig. 72); ovipositor sheath 0.7–1.0 times as long as body length; ovipositor sheath with subapical whitish band (Figs 62, 73).

###### Description.

Redescribed after a female from Guangdong (Guangzhou), length of body 15.7 mm, of fore wing 8.4 mm, and of ovipositor sheath 10.8 mm.

*Head.* Flagellum with 30 flagellomeres; length of first flagellomere 4.2 times its maximum width and 0.7 times as second flagellomere; frons finely transversely striate (Fig. 76); coronal area with oval-elongate, regular carinae encircling central ocellus and coronal teeth; three anterior coronal teeth large and acute, both posterior ones small and ear-like; vertex with three short transverse carinae between posterior ocelli, followed by finely transversely striate flattened area, striae widely posteriorly reaching to occipital carina (Fig. 74); temple with striae from vertex, but smooth ventrally (Fig. 75); temple narrowed behind eye, genal angle indistinct in dorsal view.

*Mesosoma.* Neck (Figs 68, 69) distinctly elongate, anteriorly deeply emarginate, neck at same level with middle pronotum, pronotal fold absent; neck and middle pronotum transversely spaced carinate dorsally, somewhat smooth posteriorly; posterior pronotum distinctly differentiated and steeply elevated dorsally, largely strigate and punctured, with sparse, short setosity, narrowly smooth posteriorly; prosternum transversely strigate and with some setae; mesoscutum anterior half transversely strigate, posterior half with several medium-sized foveolae and rugose; notauli distinct and straight, median groove absent; axillae rugose, with several foveolae medially, and separated basally by a large deep fovea; scutellum largely smooth, sparsely with several punctures, each bearing a short seta (Fig. 70); mesopleuron largely strigate and with small punctures, anteriorly densely whitish setose; convex part of metapleuron reticulate-foveolate, ventrally with spaced carinae and with both anterior and ventral depressions large and deep; propodeum strongly and densely reticulate-foveolate, the foveolae rather large and irregularly shaped, smooth inside, posteriorly with four smooth and large foveolae (Fig. 70).

*Wings.* Fore wing (Fig. 67): wing hyaline, vein 2-Cu1 weakly developed, 0.2 times as long as vein cu-a; pterostigma elongate and acute apically, 2.9 times as long as vein r and 21.2 times long as its maximum width; vein r and vein SR1 obtuse-angled, vein r ends 0.3 times length of pterostigma behind level of apex of pterostigma; vein SR1 subparallel to costal margin, and disappearing 0.5 times its length before reaching wing margin.

*Legs.* Hind coxa finely transversely spaced striate, basally somewhat rugose, outer side of subapex dilated (Fig. 81); hind femur swollen, finely densely strigate and sparsely with very short, soft setae, ventrally with two large acute teeth and with two obtuse basal tubercles (Fig. 71); hind tibia 1.2 times as long as hind femur; basal narrow part of hind tibia obliquely strigate and 1.5 times as long as widened part, outer side of widened part coriaceous and microreticulate, inner side of widened part basally distinctly depressed, followed by convex and setose area, apically densely setose; basitarsus parallel-sided, rather slender, its ventral length 7.2 times as long as its width, ventrally densely setose.

*Metasoma.* First tergite cylindrical, finely transversely strigate, basally somewhat rugose (Fig. 72), first tergite 11.1 times as long as its maximum width, 2.5 times as second tergite and 0.9 times as remainder of tergites; second tergite basal 0.2 rugose, rest of tergites aciculate; pygidial area narrowly impressed and setose, pygidial impression somewhat reverse V-shaped (Fig. 72); length of ovipositor sheath 0.7 times as long as body length, length of subapical whitish band 1.9 times length of dark apex (Fig. 73).

*Colour.*Largely black; frons with three longitudinal ivory streaks, one centrally and the other two along the inner orbits; temple with an ivory streak along eye; antenna brown; fore and middle legs with brown parts; subapical ovipositor sheath whitish.

*Male.*Very similar to female, but differs as follows: smaller; frons entirely vivid bright yellow, distinctly contrasting with colour of vertex (Fig. 86); a third basal tooth present and quite acute (Fig. 82).

*Variation.* Female: length of body 9–17 mm, of fore wing 5.5–12 mm, and of ovipositor 6.8–18 mm; frons with 2–3 yellowish streaks along eyes (Figs 62, 76); vein 2-CU1 of fore wing nearly absent (Figs 58, 67, 77); pterostigma 2.8–2.9 times longer than vein r and 13–21 times longer than wide (Figs 58, 67, 77); length of ovipositor sheath 0.7–1.0 times as long as body length; length of subapical whitish band1.0–2.5times as long as apical blackish part (Figs 62, 73). Male: length of body 7–13 mm, and of fore wing 5.7–6.9 mm.

###### Distribution.

Oriental China (Yunnan, Guangxi, Guangdong, Hong Kong); Vietnam.

###### Notes.

The holotype of *Foenatopus chinensis* has two yellowish streaks along the eyes on the frons (Fig. 66), as in two paratypes of *Foenatopus cerviculatus* (Chao 1964). The type series of *Foenatopus chaoi* is very similar to *Foenatopus cerviculatus* but differs by having the frons entirely yellow below the anterior coronal tooth. This is considered to be a characteristic of the males. Considering the colour of the frons they are males and not females as suggested by Chao (1964).

##### Foenatopus cinctus

(Matsumura, 1912)

http://species-id.net/wiki/Foenatopus_cinctus

Figs 87 [Fig F13] 114

Stephanus cinctus Matsumura 1912: 163; Matsumura 1930: 54.
Megischus cinctus : Belokobylskij 1995: 22.
Foenatopus cinctus : van Achterberg 2002: 168; Aguiar 2004: 23.
Foenatopus formosanus Enderlein 1913: 207, 212; Elliott 1922: 782, 784, 786; Chao 1964: 381, 387; Belokobylskij 1995: 19; Huflejt 1996: 97–98; Aguiar 2004: 26. syn. n.

###### Type material.

Lectotype of *Foenatopus cinctus*, ♀,JAPAN (HUMC): the Hokkaido University Museum, Hokkaido University Sapporo, “Lectotype, ♀, viii.1905, Okinawa, 27, *Stephanus cinctus* n. sp.”.

Lectotype of *Foenatopus formosanus*, ♀ (SDEI): “[CHINA:], Formosa, Kankau (Koshun), H. Sauter, 1912”, “*Foenatopus formosanus* Type Enderl. ♀ Dr. Enderlein det. 1913”, “22. IV. ”, “Lectotypus *Foenatopus formosanus* Enderlein, des. Belokobylskij, “92”, “LT1, 12PLT *formosanus*”, “OSUC 0021632”. Paralectotype, 8 ♀+7 ♂+1 damaged specimen (SDEI): “Kosempo Formosa H. Sauter”, “Syntypus, “Paralectotypus”, “OSUC 0021825”; “Kankau (Koshun) Formosa H. Sauter, 1912”, 7. IV.”, “*Foenatopus formosanus* Type Enderl. ♀ Dr. Enderlein det. 1913”, “Paralectotypus *Foenatopus formosanus* End. des. Belokobylskij 92”; “Hoozan Formosa H. Sauter, 1910”, 7. IX.”, “*Foenatopus formosanus* Type Enderl. ♀ Dr. Enderlein det. 1913”, “Paralectotypus *Foenatopus formosanus* End. des. Belokobylskij 92”, “OSUC 0021633”; id., “OSUC 0021826”; “Kosempo Formosa H. Sauter”, “*Foenatopus formosanus* Type Enderl. ♀ Dr. Enderlein det. 1913”, “Paralectotypus *Foenatopus formosanus* End. des. Belokobylskij 92”, “OSUC 0021830”; id., but “VI. 1912”, “OSUC 0021634”; “OSUC 0021827”; “Tainan Formosa H. Sauter 1911”, “22. VII., “*Foenatopus formosanus* Type Enderl. ♀ Dr. Enderlein det. 1913”, “Paralectotypus *Foenatopus formosanus* End. des. Belokobylskij 92”; “Taihorin Formosa H. Sauter, 1911”, “7. XI. ”, “*Foenatopus formosanus* Type Enderl. ♂ Dr. Enderlein det. 1913”, “Lectotypus *Foenatopus formosanus* Enderlein des. Belokobylskij 92”, “Coll. DEI Eberswalde”; id., but “v.[19]10”, “OSUC 0021829”; “Kankau (Koshun) Formosa H. Sauter VI. 1912”, “*Foenatopus formosanus* Type Enderl. ♀ Dr. Enderlein det. 1913”, “Paralectotypus *Foenatopus formosanus* End. des. Belokobylskij 92”, “OSUC 0021828”; id., but “♂ ”, “OSUC 0021634”; “Tainan Formosa H. Sauter 1911”, “22. VII. ”, “*Foenatopus formosanus* Enderlein Type Enderl. ♂ Dr. Enderlein det. 1913”, “Paralectotypus *Foenatopus formosanus* End. des. Belokobylskij 92”; “Anping Formosa H. Sauter, 1911”, “22. VII.”, “*Foenatopus formosanus* Type Enderl. ♂ Dr. Enderlein det. 1913”, “Paralectotypus *Foenatopus formosanus* End. des. Belokobylskij 92”, “OSUC 0021833”; “Fuhosho Formosa H. Sauter iv.[19]10”; “*Foenatopus formosanus* Type Enderl. ♂ Dr. Enderlein det. 1913”, “Paralectotypus *Foenatopus formosanus* End. des. Belokobylskij 92”, “OSUC 0021831”.

###### Other material.

1 ♀ (ZJUH): CHINA: Guangxi Longzhou Nonggang, 1982.v.20, He Jun-hua, No. 821599; 107 ♀ + 42 ♂ (HNHM): All with “Formosa, Sauter”; 69 ♀ + 31 ♂, “Kosempo, ix.1909 or x.1909”, 1 ♀, id., but “vii.1909”; 1 ♀, id., but “15–23.vi.1908”; 4 ♀ + 1 ♂, id., but “i.1910”; 1♀, id., “1–5.v.1908”; 3 ♀, “Fuhosho, iii.1909”; 1 ♀, “Mt. Hoozan, xii.1909”; 2 ♀, “Alikang, vi.1909”; 5 ♀, id., but “viii.1909”, 5 ♀, id., but “ix.1909”; 3 ♀, id., but “x.1909”; 1 ♀, “Taihorinsho, xi.1909”, 6 ♀ + 6 ♂, id., but “ix.1909”, 1 ♀, id., but “viii.1909”, 1 ♀ + 4 ♂, id., but “x.1909”, 2 ♀ + 1 ♂, id., but “xi.1909”; 1 ♀, id., but “iv.1910”.

###### Diagnosis.

Vertex transversely carinate or striate, centrally and area near eyes more or less coarsely rugose (Figs 87, 92, 102, 112); frons with 3 yellowish streaks (Figs 104, 114); temple completely yellowish, smooth and shiny (Figs 88, 103, 113); pronotum moderately robust, transversely carinate or striate in both dorsal and lateral view (Figs 93, 98, 99, 107, 108); propodeum distinctly foveolate, foveolae and interspaces in between coriaceous (Figs 94, 109); pterostigma long and apically acute (Figs 89, 97, 106); vein 2-CU1 of fore wing distinctly developed, 0.5–1.2 times as long as vein cu-a (Figs 89, 97, 106); hind femur with 2 large ventral teeth (Figs 91, 100, 110); ovipositor sheath 1.2–1.4 times as long as body; yellow subapical band of ovipositor sheath about 1.2–1.8 times as long as apical dark part (Figs 90, 101, 111).

###### Description.

Redescribed after a female from Guangxi, length of body 13.8 mm, of fore wing 9.5 mm, and of ovipositor sheath 20 mm.

*Head.* Flagellum with 34 flagellomeres; frons (Fig. 114) and vertex (Fig. 112) transversely striate; coronal teeth distinctly acute and followed by three distinct carinae between posterior ocelli; temple smooth and shiny (Fig. 113).

*Mesosoma.* Pronotum (Figs 107, 108) moderately robust and largely coriaceous, neck with 7–8 distinct spaced carinae; mesoscutum (Fig. 109) irregularly foveolate-rugose; scutellum smooth and shiny, laterally sparsely with some punctures (Fig. 109); mesopleuron largely coriaceous and with some small punctures; propodeum (Fig. 109) and convex part of metapleuron densely foveolate and coriaceous.

*Wings.* Fore wing (Fig. 106): wing hyaline, vein 2-Cu1 well developed, 0.4 times as long as vein cu-a; pterostigma elongate and acute apically and 12 times as long as its maximum width; vein r and vein SR1 obtuse-angled, vein r ends third of length of pterostigma behind level of apex of pterostigma; vein SR1 subparallel to costal margin.

*Legs.* Hind coxa transversely striate, basal third rugose; hind femur microreticulate, ventrally with two large acute teeth and with two smaller tubercles basally (Fig. 110); hind tibia coriaceous, 1.2 times as long as hind femur, basal narrow part of hind tibia 1.1 times as long as widened part, inner side of widened part basally distinctly depressed, followed by convex and setose area, apically densely setose.

*Metasoma.* First tergite finely transversely striate, 15 times as long as its maximum width, 2.1 times as second tergite and 0.8 times as remainder of tergites; second tergite basal 0.2 weakly rugose, remainder largely smooth and aciculate; pygidial impression somewhat reverse V-shaped; ovipositor sheath 1.4 times as long as body length, with subapical ivory band 1.5 times length of dark apex (Fig. 111).

*Colour.*Largely dark brown or blackish; frons brownish and with three yellowish streaks; vertex dark brown and reddish brown; temple completely yellowish; ovipositor sheath with subapical ivory band.

*Male.*Similar to female, but smaller.

*Variation.* Female: length of body 9.7–24 mm, of fore wing 6.5–12.6 mm, and of ovipositor 16–29 mm; vein 2-CU1 of fore wing 0.5–1.2 times as long as vein cu-a; length of ovipositor sheath 1.2–1.4 times as long as body length; length of subapical whitish band1.2–1.8times as long as apical blackish part. Male: length of body 8.7–21 mm, and of fore wing 4.8–10 mm.

###### Distribution.

China (Guangxi, Taiwan); Japan.

##### Foenatopus flavidentatus

(Enderlein, 1913)

http://species-id.net/wiki/Foenatopus_flavidentatus

Figs 115 [Fig F16] [Fig F17] [Fig F18] 157

Diastephanus flavidentatus Enderlein 1913: 204; Elliott 1922: 801, 809; Huflejt 1996: 97.
Foenatopus flavidentatus Baltazar 1966: 20; Belokobylskij 1995: 21; Aguiar 2004: 25.
Diastephanus simillimus Elliott 1920: 82, Elliott 1922: 802, 823. syn. n.*Foenatopus simillimus*Aguiar 2004: 36.*Diastephanus trilineatus*Elliott 1920: 81; Elliott 1922: 801, 813. syn. n.*Foenatopus trilineatus*Aguiar 2004: 38.

###### Type material.

Lectotype of *Foenatopus flavidentatus*, ♀ (SDEI): “[CHINA], Formosa, Kankau (Koshun), H. Sauter, vi. 1912”, “*Diastephanus flavidentatus* TypeEnderlein. ♀ Dr. Enderlein det. 1913”, “Lectotypus *Diastephanus flavidentatus* des. Belokobylskij 92”, “Coll. DEI Eberswalde”, “OSUC 0021645”. Paralectotype, 1 ♀ (SDEI): “Formosa, Taihorin, H. Sauter, 1911”, “7. VII.”, “*Diastephanus flavidentatus* TypeEnderlein. ♀ Dr. Enderlein det. 1913”, “Paralectotypus *Diastephanus flavidentatus* Enderlein des. Belokobylskij 92”, “Coll. DEI Eberswalde”, “OSUC 0021646”.

Holotype of *Foenatopus simillimus* (BMNH):“Type”, “B.M. Type Hym., 3.a.19”, “B.M. Type *Diastephanus simillimus* (Elliott 1920)”, “*Diastephanus similis* [sic!] E.A. Elliott, 17.xii.1919”, “[Vietnam], Tonkin: Hoabinh, Aug. 1918, R.V. de Salvaza”, “Indo China, R.V. de Salvaza, 1919–25”.

Lectotype of *Foenatopus trilineatus* here designated, ♀ (BMNH):“Type”, “B.M. Type Hym., 3.a.3”, “*Diastephanus trilineatus* E.A. Elliott, 17.xii.1919”, “[Vietnam], Tonkin: Hoabinh, Aug.1918, R.V. de Salvaza”, “Indo China, R.V. de Salvaza, 1919–25”.

###### Other material.

8 ♀ + 2 ♂ (HNHM): 1 ♀, “Taihorinsho, x.1909”; 1 ♀, id., but “xi.1909”, 3 ♀, id., but “ix.1909”; 1 ♂, id., but “viii.1909”; 1 ♀ + 1 ♂, “Kosempo, x.1909”; 1 ♀, id., but “ii.1908”; 1 ♀, “Teraso, v.1909” ; 1 ♀ (RMNH): 1 ♀, CHINA: Guangdong, Mt. Nankunshan, 20.viii.2005, Zai-fu Xu, No. 200800169; 4 ♀ (SCAU): 1 ♀, CHINA: Hong Kong, Kowloon, 10.x.2006, No. 200800172; 1 ♀, CHINA: Guangdong, Guangzhou, Tianlu Lake, 24.vi.2003, Jing-xian Liu, No. 200800164; 1 ♀, CHINA: Guangdong, Mt. Nankunshan, 20.viii.2005, Zai-fu Xu, No. 200800168; 1 ♀, CHINA: Guangdong, Liuxihe, 12.vi.2008, Zai-fu Xu, No. 200800181.

###### Diagnosis.

Vertex transversely striate (Figs 131, 146, 154); middle pale stripe of frons comparatively wide dorsally (Figs 124, 133, 148, 156; both sexes, but sometimes less in male) and base of anterior tooth of corona yellowish brown; pronotum often yellowish brown or dark brown posteriorly and usually contrasting with black mesoscutum (Figs 127, 151); pterostigma long and narrow, more or less subparallel-sided, apically acute (Figs 125, 140, 149); vein 2-CU1 of fore wing absent or weakly developed; vein r of fore wing somewhat less oblique (Figs 125, 140, 149); hind femur with 2 large teeth ventrally, teeth completely to partly pale yellowish or ivory (Figs 129, 143, 152); pygidial impression of female deep and reverse V-shaped; ovipositor sheath with ivory or pale yellowish subapical band (Figs 130, 144, 153).

###### Description.

Redescribed after the lectotype from Taiwan, female, length of body 15.6 mm, of fore wing 7.3 mm, and of ovipositor sheath 14.2 mm.

*Head.* Antenna with flagellomeres partly missing; frons finely transversely striate (Fig. 133); coronal teeth distinctly carinate and acute apically; vertex (Fig. 131) with three short transverse carinae between posterior ocelli, followed by finely transversely striate area; temple with striae from vertex, but ventrally smooth; temple smooth and shiny, narrowly rounded behind eye (Fig. 132).

*Mesosoma.* Neck (Figs 126, 127) comparatively robust and with several weak spaced carinae; pronotum largely coriaceous and somewhat microreticulate, posteriorly narrowly smooth; mesoscutum coriaceous and posteriorly largely irregularly foveolate-rugose; scutellum smooth and shiny, weakly rugulose, laterally sparsely with medium-sized foveolae (Fig. 128); mesopleuron largely coriaceous and with some small punctures, anterior fourth densely whitish setose; convex part of metapleuron densely foveolate, ventrally with spaced carinae; propodeum shallowly reticulate-foveolate and with coriaceous interspaces (Fig. 128).

*Wings.* Fore wing (Fig. 125): hyaline, vein 2-Cu1 indistinctly developed, 0.1 times as long as vein cu-a; pterostigma elongate and acute apically, twice as long as vein r and 14.8 times long as its maximum width; vein r and vein SR1 obtuse-angled, vein r ends 0.2 times length of pterostigma behind level of apex of pterostigma; vein SR1 subparallel to costal margin.

*Legs.* Hind coxa finely transversely spaced striate, basally rugose; hind femur swollen and finely microreticulate, ventrally with two large acute teeth and with one acute smaller denticle basally (Fig. 129); hind tibia coriaceous, 1.2 times as long as hind femur, basal narrow part of hind tibia 1.4 times as long as widened part, inner side of widened part basally distinctly depressed, followed by convex and setose area, apically densely setose; basitarsus parallel-sided and ventrally densely setose.

*Metasoma.* First tergite finely densely aciculate, first tergite 10.8 times as long as its maximum width, 2.3 times as second tergite and 0.8 times as remainder of tergites; second tergite basally weakly rugose, remainder largely smooth and shiny; pygidial area medially slightly convex and smooth; pygidial impression deep and reverse V-shaped; length of ovipositor sheath 0.9 times as long as body length, length of subapical yellowish band 2.6 times length of dark apex (Fig. 130).

*Colour.*Largely brownish or blackish; frons with three longitudinal yellowish streaks; vertex and pronotum reddish brown; scutellum and propodeum blackish; hind femur chestnut brown and with two large ventral teeth yellowish; ovipositor sheath with subapical yellowish band.

*Variation.* Female: length of body 11.0–15.8 mm, and of fore wing 6.0–8.2 mm.

###### Distribution.

Oriental China (Taiwan); Vietnam.

##### Foenatopus maculiferus sp. n.

urn:lsid:zoobank.org:act:77224DE9-A156-4C10-B417-F46E60AEC4D6

http://species-id.net/wiki/Foenatopus_maculiferus

Figs 158–167

###### Type material.

Holotype, ♀ (SCAU): CHINA: Hainan, Mt. Bawangling, 7.vii.2006, Jing-xian Liu, No. 200800170.

###### Diagnosis.

Head transverse in dorsal view and elliptical in lateral view (Figs 165, 166); vertex coarsely irregularly rugose (Fig. 165); frons largely yellowish, rugulose or weakly striate (Fig. 167); pronotum flat, largely rugulose, dark brown and with some orange brown parts (Figs 159, 160); scutellum medially distinctly convex and red brown; propodeum largely matt and superficially granulate, without foveolae, only posterior third foveolate-rugose (Fig. 161); pterostigma short, comparatively wide and apically obtuse (Fig. 158); both large teeth on hind femur whitish (Fig. 162); third metasomal tergite with two large ivory patches (Fig. 163); ovipositor sheath 0.7 times as long as body and with brownish subapical band 0.6 times as long as blackish apical part (Fig. 164).

###### Description.

Holotype, female, length of body 6.0 mm, of fore wing 3.5 mm, and of ovipositor sheath 4.0 mm.

*Head.* Flagellum with 23 flagellomeres; length of first flagellomere much shorter than scape and pedicel combined, 3.8 times its maximum width, and 0.8 times as second flagellomere; frons finely, transversely sculptured (Fig. 167); coronal area rugose and with acute coronal teeth; vertex with 3 transverse, curved carinae, anterior one strong and arcuate, two posterior one coarse, followed by coarsely irregularly striate-rugose, slightly convex area (Fig. 165); temple largely smooth and shiny, narrowed ventrally and behind eye (Fig. 166), head transverse in dorsal view.

*Mesosoma.* Neck (Figs 159, 160) elongate and moderately robust, anteriorly distinctly emarginate; neck and middle pronotum at same level dorsally, both coriaceous and sculptured, somewhat microreticulate; pronotal fold absent; posterior pronotum weakly striate, laterally slightly convex; pronotum laterally striate (Fig. 160); propleuron narrow and setose; prosternum largely smooth, anteriorly transversely striate; mesoscutum medially with a distinct transverse sinuate carina, area in front of it microreticulate, area behind foveolate-rugose; scutellum and axillae longitudinally striate; scutellum centrally distinctly convex, apical margin with some punctures (Fig. 161); mesopleuron anteriorly setose, dorsally smooth and ventrally largely striate; propodeum (Fig. 161) anterior 0.6 striate and largely microreticulate, laterally with several shallow foveolae laterally, propodeum posteriorly foveolate-rugose, some foveolae coalescent, inside surface coarsely coriaceous; metapleuron slightly convex and similarly sculptured as propodeum.

*Wings.* Fore wing (Fig. 158): hyaline; vein 2-CU1 0.1 times as long as vein cu-a; pterostigma wide and short, obtuse and rounded apically, 2.6 times as long as vein r and 5.2 times as its maximum width; vein r ends 0.3 times length of pterostigma behind level of apex of pterostigma; vein SR1 2.6 times as long as vein r; vein SR1 and vein r obtusely angled, vein SR1 elongate towards vein margin and ending near before reaching vein margin.

*Legs.* Hind coxa transversely striate, striations more regular posteriorly, outer side medially slightly depressed and flattened; hind femur (Fig. 162) finely transversely striate, dorsally sparsely punctuate, with two large acute ventral teeth and some denticles in between, each denticle bearing one short seta; hind tibia coriaceous and microreticulate, 1.3 times as long as hind femur, coarsely micro-areolate, basal narrowed part 1.1 times as long as widened part, inner side of widened part basally steeply depressed and followed by convex area, hind tibia apically densely setose; hind basitarsus robust, its ventral length 3.6 times its maximum width, ventrally densely setose.

*Metasoma.* First tergite transversely coarsely striate, basally more rugose, subapex much wider than basal part; first tergite 7.6 times as long as its maximum width, 2.3 times second tergite and 0.8 times as long as remainder of metasoma; second tergite subconical, basal part rugose, remainder together with rest of tergites largely smooth; pygidial area setose, laterally shallowly impressed, medially distinctly convex and lamelliform apically, pygidial impression widely reversed V-shaped (Fig. 163); length of ovipositor sheath 0.7 times as long as body length, length of subapical brownish band nearly 0.6 times length of blackish apex (Fig. 164).

*Colour.* Body mainly black or dark brown; malar space ivory; frons largely vivid yellow; vertex, pronotum, third tergite with yellow spots or patches; coronal teeth, basal 0.7 of scutellum, posterior 0.3 of propodeum and metapleuron, large part of first tergite and hind femur (except ventral teeth) red brown or reddish; middle and hind basitarsi and large teeth of hind femur whitish; ovipositor sheath largely brownish and with ivory subapex.

*Male.* Unknown.

###### Distribution.

China (Hainan).

###### Etymology.

From “macula” (Latin for patch) and “ferus” (Latin for carrying) because of the two pale patches of the third metasomal tergite.

###### Notes.

This species is similar to *Foenatopus menglongensis*, but *Foenatopus maculiferus* has the two pale patches of the third metasomal tergite (absent in *Foenatopus menglongensis*), the scutellum convex medially (flat in *Foenatopus menglongensis*), the propodeum rather matt (shiny in *Foenatopus menglongensis*) and the hind femur comparatively slender (comparatively swollen in *Foenatopus menglongensis*).

##### Foenatopus menglongensis

(Chao, 1964)

http://species-id.net/wiki/Foenatopus_menglongensis

Figs 168–173

Diastephanus menglongensis Chao 1964: 383, 385–386, 389–390, Figs I (6), III (1, 7–8), IV (2, 4, 9), V (3–4, 9).
Foenatopus menglongensis Belokobylskij 1995: 22; Aguiar 2004: 31.

###### Type material.

Holotype, ♀ (CAZB): “CHINA: Yunnan, Xishuangbanna, Menglong, 650 m. C.A.S.”, “13.vi.1958, Shu-yong Wang”, “HOLOTYPE”, “*Diastephanus menglongensis* Chao, Holotype”.

###### Diagnosis.

Head transverse in dorsal view and elliptical in lateral view (Fig. 172); vertex transversely rugose, posteriorly more coarsely (cf. Fig. 165 of *Foenatopus maculiferus*); frons densely and coarsely rugose; posterior half of pronotum dorsally finely superficially reticulate (cf. Fig. 161 of *Foenatopus maculiferus*); propodeum sparsely and superficially foveolate (Fig. 173); pterostigma short and apically obtuse (Fig. 168); vein 2-CU1 of fore wing nearly absent; hind femur swollen (Fig. 171) and with two large teeth ventrally and central large teeth whitish; third tergite entirely dark brown or blackish; ovipositor sheath largely dark brown and apically blackish (Chao, 1964) (ovipositor sheath lost in holotype in Beijing).

###### Description.

Holotype, female, length of body 7.4 mm, of fore wing 4.6 mm, and of ovipositor 6.8 mm.

*Head.* Frons finely transversely striate (cf. Fig. 167 of *Foenatopus maculiferus*); three anterior coronal teeth acute, both posterior ones wide and arcuate; vertex with 3 carinae between posterior ocelli, followed by coarsely transversely striate-rugose area (cf. Fig. 165 of *Foenatopus maculiferus*); head transverse in dorsal view; temple smooth and shiny (Fig. 172).

*Mesosoma.* Neck (Figs 169, 170) elongate and moderately robust, transversely striate, not differentiated from middle part; pronotum rather robust, transversely strigate; posterior part of distinctly convex laterally; propodeum sparsely and shallowly foveolate, posterior part somewhat rugose (Fig. 173).

*Wings.* Fore wing (Fig. 168): wing membrane hyaline; vein 2-CU1 nearly absent; pterostigma comparatively short and rounded apically, about 1.9 times as long as vein r and about 7 times its maximum width; vein r ends about 0.2 times length of pterostigma behind level of apex of pterostigma; vein SR1 nearly parallel to costal margin, vein SR1 and vein r obtusely angled.

*Legs.* Hind coxa rather slender, annularly transversely striate; hind femur (Fig. 171) finely transversely striate, ventrally with 2 large whitish teeth, basal obtuse tubercle present (and dark brown, same colour as hind femur); hind tibia coriaceous and apically densely setose.

*Metasoma.* First tergite 8.5 times as long as its maximum width, 2.2 times as second tergite and 0.9 times as long as remainder of tergites.

*Colour.* Largely dark brown or blackish. Head reddish brown, pronotum with brown tint, ventral large teeth of hind femur whitish; ovipositor brown (sheath missing).

###### Distribution.

China (Yunnan).

##### Foenatopus quadridens

(Elliott, 1920)

http://species-id.net/wiki/Foenatopus_quadridens

Figs 174–182

Diastephanus quadridens Elliott, 1920: 81, 1922: 801, 818; Dutt, 1926:12.
Foenatopus quadridens : Aguiar, 2004: 34.

###### Type material.

Holotype, ♀ (BMNH): “Type”, “B.M. Type Hym., 3.a.4”, “*Diastephanus quadridens* E.A. Elliott, 17.xii.1919”, “[Laos], Lunag Prabang, 5.x. 1917, R.V. de Salvaza”, “Indo China, R.V. de Salvaza, 1919–25”.

###### Diagnosis.

Head more or less globose in dorsal view; vertex transversely striate (Fig. 180); frons of female comparatively coarsely sculptured, without distinct pale lateral streaks (Fig. 182); pronotum robust in dorsal view and its posterior half distinctly striate or carinate (Figs 175, 176); pterostigma long and narrow, more or less subparallel-sided, apically acute (Fig. 174); vein 2-CU1 of fore wing comparatively short and about 0.3 times as long as vein cu-a (Fig. 174); hind femur with 4 medium-sized to large teeth (Fig. 177); ovipositor sheath completely black (Fig. 178), 1.15 times as long as body.

###### Distribution.

Laos.

###### Note:

This species is not known from China at present. It is included in the key because it may occur in South China.

##### Foenatopus ruficollis

(Enderlein, 1913)

http://species-id.net/wiki/Foenatopus_ruficollis

Figs 183 [Fig F24] 210

Diastephanus ruficollis Enderlein 1913: 205; Elliott 1922: 801–802, 819; Chao 1964: 383, 388; Huflejt 1996: 97.
Foenatopus ruficollis Belokobylskij 1995: 21; Aguiar 2004: 35.
Diastephanus trilobatus Elliott 1920: 82; Elliott 1922: 801, 816. syn. n.*Foenatopus trilobatus* (Elliott): Aguiar 2004: 38.

###### Type material.

Lectotype of *Foenatopus ruficollis*, ♀ (SDEI): “[CHINA], Formosa, Kankau (Koshun) H. Sauter, vi. 1912”, “*Diastephanus ruficollis* TypeEnderl. ♀ Dr. Enderlein det. 1913”, “Lectotypus *Diastephanus ruficollis* Enderlein des. Belokobylskij 92”, “Coll. DEI Eberswalde”, “SDEI 007”, “OSUC 0021923”. Paralectotypes, 2 ♂ (SDEI): “[China], Kankau (Koshun) Formosa H. Sauter, v. 1912”, “*Diastephanus ruficollis* TypeEnderl. ♂ Dr. Enderlein det. 1913”, “Paralectotypus *Diastephanus ruficollis* End. des. Belokobylskij 92”, “Coll. DEI Eberswalde”, “OSUC 0021924”; id., “OSUC 0021925”.

Holotype of *Foenatopus trilobatus*,♀ (BMNH): “Type”, “B.M. Type Hym., 3.a.35”, “*Diastephanus trilobatus* E.A. Elliott, 17.xii.1919”, “[Vietnam], Tonkin: Hoabinh, Aug.1918, R.V. de Salvaza”, “Indo China, R.V. de Salvaza, 1919–25”.

###### Other material.

4 ♀ (SCAU): 1 ♀, CHINA: Guangdong, Arboretum of Nanling, 21.vii.2008, Chun-dan Hong, No. 200800182; 1 ♀, CHINA: Guangdong, Mt. Chebaling, 10.vii.2003, Jing-xian Liu, No. 200800165; 1 ♀, CHINA: Guangdong, Mt. Nanling, 6.x.2004, Zai-fu Xu, No. 200800166; 1 ♀, CHINA: Hainan, Mt. Jianfengling, 5–7.vi.2007, Li-qiong Weng, No. 200800177; 1 ♀ (ZJUH): CHINA: Guangxi, Longzhou, Nonggang, 18.v.1982, Jun-hua He, No. 821489; 1 ♀ (RMNH): CHINA: Guangdong, Mt. Nanling, 1–6.x.2004, Zai-fu Xu, No. 200800167.

###### Diagnosis.

Headtransverse in dorsal view and (Figs 189, 198, 208) globose in lateral view (Figs 190, 199, 209); vertex coarsely transversely carinate-rugose (Figs 189, 198, 208); face coarsely transversely striate, yellow patch on frons variable in size (Figs 191, 200, 210); pronotum transversely distinctly or weakly striate (Figs 184, 185, 193, 194, 202, 203); propodeum densely and distinctly to shallowly foveolate (Figs 186, 204); pterostigma moderately short and comparatively wide (Figs 183, 192, 201); vein 2-CU1 of fore wing nearly absent (Figs 183, 192, 201); hind femur with 3 large teeth ventrally (Figs 187, 195, 206); ovipositor sheath nearly completely brownish or black (Figs 188, 196, 207), without subapical band and about as long as body.

###### Description.

Redescribed after a female from Guangdong (Nanling), length of body 10.3 mm, of fore wing 5.6 mm, and of ovipositor 10.5 mm.

*Head.* Flagellum with 28 flagellomeres; length of first flagellomere 3.0 times its maximum width, and 0.6 times as second flagellomere; frons transversely striate, sculpture coarser near the central frontal coronal tooth (Fig. 210); three anterior coronal teeth acute, both posterior ones widely separated and arcuate; between posterior ocelli with three strong, transverse carinae, third one interrupted medially; vertex irregularly coarsely transversely carinate (Fig. 208); temples largely smooth but with some sculpture originating from vertex; temple indistinctly protruding (Fig. 209), head transverse in dorsal view (Fig. 208).

*Mesosoma.* Neck (Figs 202, 203) robust, dorsally with several irregular rugae, anteriorly distinctly emarginate, postero-dorsally at same level as middle pronotum; middle and posterior parts of pronotum dorsally transversely striate and coriaceous, laterally obliquely strigate, lateral convex posterior pronotum rugose; prosternum largely glabrous and coriaceous; mesoscutum anterior half transversely strigate, remainder strongly carinate-rugose; median sulcus and notauli distinct on anterior half; axillae strigate, more finely so laterally, separated basally by a large fovea and form scutellum by reverse V-shaped impressions; scutellum largely coarsely strigate and somewhat rugose laterally (Fig. 204); mesopleuron sparsely shallowly foveolate-rugose, anteriorly covered with whitish pubescence; metapleuron strongly reticulate-foveolate, ventrally with transverse carinae; propodeum strongly reticulate-foveolate, foveolae rather large, some foveolae posteriorly coalescent, and coarse inside (Fig. 204).

*Wings.* Fore wing (Fig. 201): wing hyaline, vein 2-Cu1 nearly absent; pterostigma widened and comparatively short, obtuse apically, 2.3 times as long as vein r and 7.4 times as its maximum width; vein r ends 0.2 times length of pterostigma behind level of apex of pterostigma; vein 2-SR 2.1 times as long as vein r; vein SR1 and vein r obtuse-angled, vein SR1 curved towards costal margin and disappearing 0.5 times its length before reaching wing margin.

*Legs.* Hind coxa subcylindrical, anteriorly rugose and posteriorly transversely spaced striate; hind femur finely transversely strigate, with three large ventral teeth and some denticles between posterior ones (Fig. 206); hind tibia 1.4 times as long as hind femur, basal narrow part of hind tibia coriaceous and oblique strigate, 1.4 times as long as widened part, outer side of widened part coriaceous and obliquely strigate, inner side of widened part basally depressed, followed by coriaceous, microareolate convex area, apically densely setose (Fig. 206); hind basitarsus parallel-sided, rather slender, its ventral length 5.3 times as long as its width, ventrally densely setose.

*Metasoma.* First tergite subcylindrical, coarsely transversely striate, 11.3 times as long as its maximum width, 2.2 times as second tergite and 0.7 times as remainder of tergites; second tergite basal 0.3 rugose, medial part coriaceous and microareolate, apical 0.1 aciculate; the rest of tergites largely aciculate; pygidial area with short hair as surroundings, slightly impressed laterally and centrally convex, pygidial impression reversed V-shaped; ovipositor sheath completely dark brown (Fig. 207), as long as body length.

*Colour.*Almost entirely brownish or dark brown; frons near antennae ivory; malar space ivory; temple with light brownish patch along half of eye; hind basitarsus whitish; ovipositor sheath totally brownish.

*Male.* Unknown.

*Variation.* Female: length of body 8.5–14.5 mm, of fore wing 5.6–5.9 mm, and of ovipositor sheath 10.5–14.8 mm; pterostigma 2.1–2.3 times longer than vein r; shape and size of ivory or brownish part on frons variable.

###### Distribution.

Oriental China (Guangdong, Guangxi, Taiwan, Hainan); Vietnam.

**Note.** The lectotype of *Foenatopus ruficollis* differs mainly by the more yellowish brown body and the less developed sculpture on body.

##### Foenatopus yangi sp. n.

urn:lsid:zoobank.org:act:F82DF5A8-11F3-495F-95A1-9D95E917D6F1

http://species-id.net/wiki/Foenatopus_yangi

Figs 211–220

###### Type material.

Holotype, ♀ (CAFB): CHINA: Guangdong, Deqing, on trunk of *Cinnamomum loureirii* tree with Xyloryctidae larvae, 27.iv.1997, Yi-zhen Li.

###### Diagnosis.

Body largely dull; vertex transversely striate (Fig. 218); frons with 3 yellowish streaks (Fig. 220); yellowish patch on temple weakly defined (Fig. 219); pygidial impression indistinct (Fig. 216); pronotum anteriorly with some fine rugulae, laterally coriaceous or finely reticulate (Figs 212, 213); scutellum matt and medially coriaceous (Fig. 214); pterostigma moderately elongate; vein 2-CU1 of fore wing absent (Fig. 211); both large teeth on hind femur mostly yellowish (Fig. 215); ovipositor sheath 0.9 as long as body length; subapical ivory band of ovipositor sheath 2.5 times as long as its blackish apex (Fig. 217).

###### Description.

Holotype, female, length of body 13.4 mm, of fore wing 6.7 mm, and of ovipositor sheath 11.6 mm.

*Head.* Flagellum with 29 flagellomeres; frons coarsely transversely striate (Fig. 220); coronal area rugose and coronal teeth acute; vertex slightly convex and finely transversely striate, striations posteriorly reaching occipital carina (Fig. 218); temples smooth and matt, narrowed and rounded behind eyes (Fig. 219).

*Mesosoma.* Neck (Fig. 212) elongate and rather robust, at about same level as middle pronotum, anteriorly distinctly emarginate, dorsally with weak transverse carinae, medio-longitudinally slightly impressed; middle pronotum transversely striate; posterior pronotum distinctly elevated and rugose dorsally, convex laterally (Fig. 213); mesoscutum anterior half densely striate and posteriorly coarsely foveolate; axillae coarsely rugose; scutellum largely smooth and matt, sparsely with a few punctures (Fig. 214); mesopleuron anterior 0.3 rugose and covered with setosity, remainder largely aciculate; convex part of metapleuron rather robust, largely strongly reticulate-foveolate; both anterior and ventral foveae large; propodeum largely spaced foveolate, foveolae medium-sized, circular and well defined, with microsculptured interspaces (Fig. 214).

*Wings.* Fore wing (Fig. 211): wing membrane hyaline; vein 2-CU1 weakly developed and 0.1 times as long as vein cu-a; pterostigma moderately elongate and somewhat rounded apically, 2.9 times as long as vein r and 13.7 times as its maximum width; vein r ends 0.2 times length of pterostigma behind level of apex of pterostigma; vein SR1 nearly parallel to costal margin, vein SR1 and vein r obtusely angled.

*Legs.* Hind coxa largely transversely striate, anterior 0.3 rugose; hind femur densely transversely strigate, ventrally with two large acute ivory teeth, basally with 2 obtuse tubercles (Fig. 215); hind tibia coriaceous, 1.2 times as long as hind femur, basal narrow part of hind tibia 1.1 times as long as widened part; inner side of widened part basally depressed, medially convex and granulate, apically densely setose (Fig. 216); length of basitarsus 5.0 times as its width and ventrally densely bristly setose.

*Metasoma.* First tergite finely transversely striate, 14.2 times as long as its maximum width, 2.2 times second tergite and 0.9 times remainder of metasoma; pygidial area indistinctly differentiated, apically truncate, laterally slightly impressed, medially weakly convex and granulate; subapical ivory band of ovipositor sheath 2.5 times as long as apical blackish part (Fig. 217); ovipositor sheath 0.9 times as long as body length.

*Colour.* Brownish to blackish; frons with 3 longitudinal yellowish streaks, one centrally and the other two along the inner orbits; temple ventrally brownish along eye; wing membrane hyaline, wing veins light brownish; pterostigma ivory; pronotum, hind femur and hind tibia, and metasoma largely dark brown; fore and middle legs largely brownish and with some whitish parts; large ventral teeth of hind femur ivory; subapical ivory band of ovipositor sheath 2.5 times as long as apical blackish part.

*Male.* Unknown.

###### Distribution.

Oriental China (Guangdong).

###### Etymology.

This species is named after Prof. Zhong-qi Yang (CAFB) who generously loaned the specimen for the study.

#### Genus Megischus

Brullé, 1846

http://species-id.net/wiki/Megischus

Figs 221 [Fig F28] [Fig F29] [Fig F30] 251

Megischus Brullé 1846: 537. Type species (designated by Viereck 1914): *Megischus annulator*Brullé 1846 [= *Megischus furcatus* (Lepeletier & Serville, 1835)].

##### Diagnosis.

Temple without pale yellowish streak along eye, at most with a ventral patch (Figs 226, 245); pronotum robust and without transverse protuberance; vein 1-SR of fore wing differentiated with first discal cell present because of presence of vein 1-SR+M; vein 1-M of fore wing straight or nearly so; first subdiscal cell of fore wing comparatively narrow basally (Figs 228, 239, 240); hind wing without trace of vein cu-a; hind femur largely smooth and with some punctures, ventrally with 2 large teeth (Figs 230, 238, 247, 251); inner side of hind tibia usually with wide submedial depression (Fig. 248); outer side of hind tibia without oblique striae or rugulosity and ventrally evenly rounded (Fig. 249); hind tarsus of female with three tarsomeres (Fig. 238); ovipositor sheath with ivory subapical band (Fig. 250).

##### Distribution.

Cosmopolitan.

##### Notes.

Before this study, 83 species in the genus *Megischus* were known worldwide, of which 3 species were known from China. In this paper, these three species of the genus are listed from China. A key to species of the genus *Megischus* from China is as follows:

##### Key to species of the genus *Megischus* from China

(after Hong et al. 2010)

**Table:** 

1	Temple narrowly rounded medially behind eye in dorsal view (Fig. 223); neck postero-dorsally at about same level as middle pronotum (Fig. 234); vein cu-a of fore wing strongly reclivous (Fig. 239); hind basitarsus about 4 times as long as wide (Fig. 236); [without distinct pronotal fold and without a cavity (Fig. 234); vein 1-M of fore wing about 5 times as long as vein 1-SR and 1.2 times vein m-cu (Fig. 239); widened part of hind tibia of female nearly straight or weakly concave ventrally (Fig. 238); ivory part of ovipositor sheath about twice as long as dark apical part]	*Megischus chaoi* van Achterberg & Yang, 2004
–	Temple medially roundly convex behind eye in dorsal view (Figs 226, 244); neck at lower level than middle pronotum postero-dorsally (Figs 222, 242); vein cu-a of fore wing weakly reclivous or subvertical (Figs 228, 240); hind basitarsus 3.0–3.5 times as long as wide (Fig. 231)	2
2	Temple slightly convex behind eye (Fig. 244); pronotal fold distinct and with a cavity below it (Figs 241, 242); vein 1-M of fore wing 4.2–5.5 times as long as vein 1-SR and 1.1–1.3 times vein m-cu (Fig. 240); first tergite largely transversely striate or striate-rugose; head largely blackish or dark brown; widened part of hind tibia of female distinctly concave ventrally (Figs 248, 249), but straight in male (Fig. 251); [whitish or ivory part of ovipositor sheath 0.7–2.0 times as long as dark apical part]	*Megischus ptosimae* Chao, 1964
–	Temple distinctly convex behind eye (Fig. 226); pronotal fold absent (Figs 221, 222); vein 1-M of fore wing about 2.2 times as long as vein 1-SR and 0.9 times vein m-cu (Fig. 228); first tergite largely smooth and shiny dorsally (Fig. 229); head largely orange brown (Figs 225, 226, 227); widened part of hind tibia of male nearly straight ventrally (Fig. 230)	*Megischus aplicatus* Hong, van Achterberg & Xu (male)

##### Megischus aplicatus

Hong, van Achterberg & Xu, 2010

http://species-id.net/wiki/Megischus_aplicatus

Figs 221–232

Megischus aplicatus Honget al. 2010: 61.

###### Type material.

Holotype, ♂ (ZJUH): CHINA: Hubei, Shennongjia, viii.1982, Shang-bo Shi, No. 870112.

###### Diagnosis.

Head largely orange brown; temple medially distinctly convex behind eye in dorsal view (Fig. 225); neck at lower level than middle pronotum postero-dorsally (Figs 221, 222); pronotal fold absent (Fig. 221); vein 1-M of fore wing about 2.2 times as long as vein 1-SR and 0.9 times vein m-cu (Fig. 228); vein cu-a of fore wing weakly reclivous or subvertical (Fig. 228); widened part of hind tibia of male nearly straight ventrally (Fig. 230); hind basitarsus 3.5 times as long as wide (Fig. 231); first tergite largely smooth and shiny dorsally (Fig. 229).

###### Description.

See Hong et al. (2010).

###### Distribution.

China (Hubei).

###### Notes.

The name of this species is derived from the Latin “a-” and “plicatus” which means without fold, because this species has no pronotal fold on the pronotum.

##### Megischus chaoi

van Achterberg & Yang, 2004

http://species-id.net/wiki/Megischus_chaoi

Figs 233–239

Megischus ruficeps Saussure: Chao 1964: 378–379, 387–388, Figs II (1–2), IV (13); Belokobylskij 1995: 22.
Megischus chaoi van Achterberg: van Achterberg and Yang 2004: 109, 111–112; Hong et al. 2010: 61.

###### Diagnosis.

Temple medially roundly narrowed behind eye in dorsal view; neck postero-dorsally at about same level as middle pronotum, without distinct pronotal fold and without a cavity (Figs 234, 235); vein 1-M of fore wing about 5 times as long as vein 1-SR and 1.2 times vein m-cu; vein cu-a of fore wing strongly reclivous (Fig. 239); widened part of hind tibia of female nearly straight or weakly concave ventrally (Fig. 238); hind basitarsus about 4 times as long as wide (Fig. 236); ivory part of ovipositor sheath about twice as long as dark apical part.

###### Type material.

Holotype, ♀ (CPPF): “[CHINA], Fukien [=Fujian], Foochow [=Fuzhou], 10.vi.1954, H. F. Chao coll.”; “*Megischus ruficeps* Hsiu-Fu Chao det.” (van Achterberg and Yang 2004).

###### Description.

See van Achterberg and Yang (2004).

###### Distribution.

China (Fujian).

###### Notes.

This species is named in honour of the late Prof. Dr Hsiu-Fu Chao (Fuzhou) for his important contributions to our knowledge of the entomofauna of China (van Achterberg and Yang 2004).

##### Megischus ptosimae

Chao, 1964

http://species-id.net/wiki/Megischus_ptosimae

Figs 240 251

Megischus ptosimae Chao 1964: 378–379, 387–388, Figs I (1), II (3–4), IV (14), V (6, 10); van Achterberg and Yang 2004: 112–115; Hong et al. 2010: 61.

###### Material.

6 ♀+ 1 ♂: 2 ♀ (SCAU), CHINA: Guangdong, Chebaling, 26.vii.2008, Zai-fu Xu, No. 200800183; Chun-dan Hong, No. 200800184; 1 ♀ (ZJUH), CHINA: Sichuan, Nanchong, 24.vi.1940, No. 65021.10; 1 ♀ (ZJUH), CHINA: Zhejiang, Quxian, 24.v.1959, Zheng-nan Zhou, No. 5931.1; 1 ♂ (ZJUH), CHINA: Zhejiang, Hangzhou, 7. vii. 1980, Ben-yue Zhang, No. 810242. 1 ♀ (CAFB): “China: Shaanxi, Yangling, 30.viii.1994, ovipositing on larvae of Buprestidae on *Prunus* sp., Zhong-qi Yang”, “det C. van Achterberg, 2004”. 1 ♀ (RMNH): “China: Fujian, Fuzhou, Westlake, 8.v.1963, Zhen-cai Zhang, RMNH 03”, “det C. van Achterberg, 2003”.

###### Diagnosis.

Head largely blackish or dark brown; temple medially slightly convex behind eye in dorsal view (Fig. 244); neck at lower level than middle pronotum postero-dorsally (Fig. 242); pronotal fold distinct and with a cavity below it (Figs 241, 242); vein 1-M of fore wing 4.2–5.5 times as long as vein 1-SR and 1.1–1.3 times vein m-cu; vein cu-a of fore wing weakly reclivous or subvertical (Fig. 240); widened part of hind tibia of female distinctly concave ventrally (Figs 248, 249), but straight in male (Fig. 251); hind basitarsus 3.0–3.5 times as long as wide; first tergite largely transversely striate or striate-rugose; whitish or ivory part of ovipositor sheath 0.7–2.0 times as long as dark apical part (Fig. 250).

###### Description.

Redescribed after a female from Guangdong (Chebaling), length of body 13.9 mm, of fore wing 7.5 mm, and of ovipositor sheath 12.8 mm.

*Head.* Flagellum with 32 flagellomeres; first flagellomere slender, 4.0 times its maximum width, and 0.9 times as second flagellomere; frons (Fig. 246) coarsely reticulate-rugose and covered with few scattered short setae; coronal area somewhat reticulate and with some short carinae, three anterior coronal teeth large, both posterior ones small and as sinuate transverse and wide lamellae; vertex (Fig. 244) with three coarse carinae, two anterior ones strong and arcuate, the last one much shorter, followed by broadly reticulate-rugose and sparsely pubescent area, sculpture postero-dorsally gradually weaker near occipital carina; temple (Fig. 245) smooth and shiny, except for small punctures with associated setae on ventral half of temple and narrowly along eye orbit, temple slightly bulging behind eye in dorsal view.

*Mesosoma.* Neck (Figs 241, 242) moderately slender, anteriorly moderately emarginate, medio-dorsally largely smooth, laterally with three pairs of strong, oblique carinae, anterior two pairs narrowly interrupted dorsally, the posterior pair curved backwards and apically widely separated; neck postero-dorsally at much lower level than middle pronotum, resulting in a distinct, deep cavity below pronotal fold; pronotal fold stout and strongly developed; middle pronotum dorsally at about same level with posterior pronotum and strongly striate-rugose; posterior pronotum largely foveolate and with smooth interspaces, foveolae with associated long setae and some foveolae dorsally coalescent; lateral ventral groove narrowly impressed and smooth, area below it obliquely rugose; propleuron coriaceous and setose; prosternum densely foveolate, foveolae circular and setose; mesoscutum largely densely and strongly foveolate, some foveolae coalescent, generating areolation; notauli and median groove distinct, formed by closely aligned foveolae; axillae foveolate, foveolae deep and separated by about their diameter, axillae separated basally by a large fovea; scutellum (Fig. 243) irregularly distributed with circular foveolae and with smooth interspaces, foveolae laterally denser than dorsally; mesopleuron largely coarsely and densely punctate-rugose, each foveola bearing long and thin seta; convex part of metapleuron densely foveolate and with long whitish setosity, antero-ventrally crenulate and with both anterior and ventral depressions rather deep; propodeum (Fig. 243) dorsally almost glabrous, completely with shallow, circular foveolae, most foveolae separated by about 0.1 of their diameter, some of them coalescent.

*Wings.* Fore wing (Fig. 240): wing subhyaline, and surface evenly bristly; vein M+CU1 with four short, erect, equidistant setae; basally vein 1–1A with about 10 erect, approximately equidistant setae grouped at base; vein 1-M 4.8 times as long as vein 1-SR and 1.1 times vein m-cu; vein 2-SR 1.4 times vein r; vein r ends 0.4 length of pterostigma behind level of apex of pterostigma; vein 1-SR 0.5 times as long as parastigmal vein; vein cu-a postfurcal and subvertical; vein 3-CU1 entirely nebulous and curved apically; first subdiscal cell slightly open posteriorly.

*Legs.* Hind coxa rather robust, subelliptical, with long whitish setosity strongly inclined towards apex, coarsely spaced punctate-rugose, but posteriorly transversely striate; hind femur (Fig. 247) with scattered punctures and largely smooth and shiny interspaces, each puncture bearing one long whitish seta, hind femur ventrally with two large teeth and several minute teeth in between and behind apical one; hind tibia (Figs 248, 249) 1.4 times longer than hind femur, largely sparsely punctate and with long setae, basal narrow part of hind tibia 0.3 times as wide as widest part; outer side of widened part basally widely and rather steeply depressed and ventrally strongly concave, apical part rather robust; inner side of widened part basally slightly depressed and smooth, followed by coarsely granulate area, apically densely setose; hind tarsus bristly setose ventrally, hind basitarsus robust and somewhat widened apically.

*Metasoma.* First tergite transversely striate-rugose, 6.2 times as long as its maximum width, 1.7 times as second tergite and 0.6 times as remainder of metasoma; basal 0.2 of second tergite weakly rugose, remainder largely coriaceous and somewhat microareolate; remainder of tergites transversely densely and finely aciculate and sparsely short setose, setae on last two tergites denser; pygidial area with moderately long setae, laterally distinctly impressed and centrally convex, medially granulate; pygidial impression somewhat reversed U-shaped; length of ovipositor sheath 0.9 times as long as body length, length of subapical whitish band nearly 1.6 times length of dark apical part.

*Colour.* Largely black or dark brown; most of head, mesosoma and hind coxa black or blackish; malar space and basitarsi ivory; temple and basal 0.3 of hind tibia orange or reddish brown; wing membrane light brownish; antenna, veins, pterostigma, legs (except basal 0.3 of hind tibia and basitarsus), and most of metasoma brown or dark brown; mandible except its apex black; clypeus, fore and middle legs, lateral part of metasoma (except first tergite) yellowish brown; ovipositor sheath largely black and with whitish subapical band.

*Male.* Very similar to female, but smaller, length of body 12.7 mm, and of fore wing 6.3 mm; widened part of hind tibia ventrally not concave but more or less straight (Fig. 251); hind tarsus with five tarsomeres.

*Variation.* Female: length of body 9–20.7 mm, of fore wing 6.8–12.5 mm, and of ovipositor sheath 9–21.0 mm; flagellum with 28–36 flagellomeres; vein 1-M 4.2–5.5 times as long as vein 1-SR and 1.1–1.3 times vein m-cu; first tergite 6.1–8.4 times as long as its maximum width, 1.7–2.4 times second tergite and 0.6–0.8 times remainder of metasoma; length of ovipositor sheath 0.9–1.0 times as long as body length, length of subapical whitish or ivory band 0.7–2.0 times length of dark apex. Male: length of body 6.5–17 mm.

###### Biology.

A parasitoid of *Ptosima chinensis* Mars. (Buprestidae) larvae in peachtrees, and of Buprestidae in other *Prunus* species (Chao 1964).

###### Distribution.

China (Shaanxi, Zhejiang, Sichuan, Fujian, Guangdong).

###### Note.

The name of this species is derived from the generic name of its host, *Ptosima chinensis* (Chao 1964).

#### Genus Parastephanellus

Enderlein, 1906

http://species-id.net/wiki/Parastephanellus

Figs 252 [Fig F33] [Fig F34] [Fig F35] [Fig F36] [Fig F37] [Fig F38] [Fig F39] [Fig F40] [Fig F41] [Fig F42] [Fig F43] [Fig F44] [Fig F45] 350

Parastephanus Enderlein 1905: 474 (not Haeckel 1881). Type species (by original designation): *Stephanus pygmaeus* Enderlein, 1901.
Parastephanellus Enderlein 1906: 301. Type species (by original designation): *Stephanus pygmaeus* Enderlein 1901.

##### Diagnosis.

Temple with pale yellowish streak along eye (Figs 257, 270, 304, 330); neck short and comparatively robust, without pronotal fold or distinct transverse carinae (Figs 253, 266, 279, 288, 300, 313, 326, 339); metapleuron robust; vein 2-CU1 of fore wing completely developed; veins 2-SR and 2-SR+M of fore wing present, sometimes only pigmented; vein 1-SR of fore wing straight (Figs 252, 265, 278, 287, 299, 312, 325, 338); outer side of hind tibia with distinct oblique striae or carinae ventrally, rarely without striae or ventral carina (Figs 263, 276, 282, 310, 323, 336, 349); ovipositor sheath without ivory subapical band (Figs 264, 276, 311, 324, 337, 350).

##### Distribution.

Australian, Palaearctic and Oriental.

##### Notes.

Before this study, 56 species in the genus *Parastephanellus* were known worldwide, of which 2 species were known from China. In this paper, 3 species of this genus are new to science, one species is synonymized (*Parastephanellus austrochinensis* Belokobylskij, 1995 for *Parastephanellus brevistigma* Enderlein, 1913) and one species, *Parastephanellus matsumotoi*, is a new record to China; altogether 5 species are known from China. A key to species of *Parastephanellus* from China and adjacent regions follows:

##### Key to species of the genus *Parastephanellus* from China and adjacent regions

**Table:** 

1	Pygidial process of female rounded apically (Fig. 347); propodeum densely foveolate and interspaces small (Fig. 341) and yellowish streak reaching occipital carina (Fig. 342); apical large tooth of hind femur comparatively wide and more or less obtuse (Fig. 349); [pterostigma about 5 times as long as wide]	*Parastephanellus zhejiangensis* sp. n.
–	Pygidial process of female horn-shaped apically (Figs 261, 274, 308, 321, 334), but unknown of *Parastephanellus brevistigma*; propodeum usually sparsely foveolate and interspaces mostly wider than diameter of punctures or subequal, and if densely foveolate then yellowish streak remain far removed from occipital carina (Figs 255, 268, 281, 290, 302, 315, 328); apical large tooth of hind femur comparatively narrow and more or less acute (Figs 262, 276, 294, 310, 323, 335)	2
2	Pterostigma distinctly widened and comparatively short, 4–5 times as long as wide (Figs 278, 287); temple yellowish and without distinctly contrasting ivory streak (Figs 285, 292; male, but more or less developed in female); frons of female brownish-yellow (Figs 286, 293); pronotum comparatively short and lateral length 0.8–0.9 times its maximum width (Figs 279, 288); [pronotum in dorsal view superficially sculptured and shiny; base of second tergite brownish-yellow; first tergite about as long as remainder of metasoma in male, 0.8 times in female; frons transversely rugose]	*Parastephanellus brevistigma* Enderlein, 1913
–	Pterostigma hardly widened and longer, 5–6 times as long as wide (Figs 252, 265, 299, 312, 325); temple orange or dark brown or blackish and with a yellowish or ivory streak distinctly contrasting with surroundings (Figs 257, 304, 317, 330); frons of female dark brown or orange brown (Figs 258, 271, 305, 318, 331); pronotum somewhat longer and its lateral length equal to its maximum width (Figs 253, 279, 300, 313, 326)	3
3	Middle basitarsus of female about 6 times as long as its medial with; first discal cell of fore wing comparatively narrow and its length about 3.1 times as long as vein 1-M (Fig. 265); first metasomal tergite of female comparatively robust; [hind coxa of female comparatively short and wide in lateral view (Figs 272, 273)]	*Parastephanellus brevicoxalis* sp. n.
–	Middle basitarsus of female 8–12 times as long as its medial with; first discal cell of fore wing comparatively wide and its length 2.2–2.7 times as long as vein 1-M (Figs 252, 299, 312, 325); first tergite of female comparatively slender	4
4	Hind femur shiny and largely smooth (Fig. 262); distance from dorsally widened pale streak on temple to occipital carina in dorsal view less than width of streak (Fig. 256); temples strongly angulate in dorsal view (Fig. 256); pronotum chestnut-brown (Figs 253, 254); frons orange-brown (Fig. 258)	*Parastephanellus angulatus* sp. n.
–	Hind femur matt or with satin sheen, largely coriaceous (Figs 309, 322, 335); distance from dorsally widened pale streak on temple to occipital carina in dorsal view more than width of streak (Figs 304, 316, 330); temples moderately angulate to rather rounded in dorsal view (Figs 303, 316, 329); pronotum dark brown or blackish (Figs 300, 313, 326); frons largely dark brown	*Parastephanellus matsumotoi* van Achterberg, 2006

##### Parastephanellus angulatus sp. n.

urn:lsid:zoobank.org:act:28086D10-6F18-48CA-B955-163A6E9AB1A4

http://species-id.net/wiki/Parastephanellus_angulatus

Figs 252 264

###### Type material.

Holotype,♀ (SCAU): CHINA: Hainan, Mt. Jianfengling, 5–7.vi.2007, Li-qiong Weng, No. 200800178.

###### Diagnosis.

Frons densely foveolate-rugose and with obvious medial groove (Fig. 258); temples dark brown and distinctly angulate in dorsal view (Fig. 256); yellowish streak on temple in dorsal view distinctly contrasting with surroundings and reaching vertex but not reaching occipital carina (Figs 256, 257); pronotum transversely rugose in dorsal view (Fig. 253); propodeum sparsely foveolate and with coriaceous interspaces (Fig. 255); pterostigma long and about 6 times as long as wide (Fig. 252); hind femur distinctly swollen, obtuse subbasal tooth comparatively large and apical large tooth comparatively narrow and acute (Fig. 262); hind tibia ventrally with regular weak carinae and basal narrow part of outer side mainly finely aciculate (Figs 262, 263); pygidial process of female medium-sized (Fig. 261).

###### Description.

Holotype, female, length of body 13.1 mm, of fore wing 8.4 mm, and of ovipositor sheath 19.3 mm.

*Head.* Flagellum with 32 flagellomeres; length of first flagellomere 4.9 times its maximum width, and 0.6 times second flagellomere; frons coarsely reticulate-rugose, frontal carina present (Fig. 258); three anterior coronal teeth acute, lobe-shaped, both posterior ones as sinuate transverse and wide lamellae; coronal area largely smooth, followed by three coarse and curved transverse carinae, anterior two carinae rather strong and posterior carina weaker; sculpture of vertex varies from undulate-rugose anteriorly to transversely striate posteriorly, narrowly reaching occipital carina (Fig. 256); temple largely smooth and shiny, except with a few punctures bearing setae ventrally (Fig. 257); temple strongly angularly protruding in dorsal view (Fig. 256).

*Mesosoma.* Neck short and rather stout, anteriorly distinctly emarginate, laterally with pairs of transverse striae, medio-dorsally smooth and posteriorly at much lower level than middle pronotum (Figs 253, 254); pronotal fold absent medially and distinct laterally; remainder of pronotum largely transversely striate and with sparse setae dorsally and posteriorly narrowly smooth and shiny, laterally somewhat striate; middle pronotum not distinctly differentiated from posterior pronotum, lateral oblique groove of pronotum smooth and rather impressed, ventral area below it somewhat sculptured (Fig. 254); propleuron coriaceous and densely setose; anterior third of mesoscutum transversely striate, posteriorly strongly foveolate-rugose; notauli and median groove complete and distinct, formed by closely aligned foveolae; axillae largely coarsely rugose, forming some irregular foveolae; scutellum irregularly aciculate medially and with rather large circular foveolae laterally (Fig. 255); mesopleuron largely rugose and with long, whitish setae, anteriorly more densely pubescent than posteriorly; convex part of metapleuron strongly reticulate-rugose and with rather long whitish setae, ventral part with spaced carinae and smooth interspaces; propodeum foveolate, foveolae circular, medium-sized and with aciculate interspaces, posterior foveolae close to each other and somewhat reticulate (Fig. 255).

*Wings.* Fore wing (Fig. 252): vein 1-M 2.2 times as long as vein 1-SR and 1.3 times vein m-cu; vein cu-a slightly antefurcal and distinctly curved; vein 2-SR 1.6 times as long as vein r; vein r ends 0.4 times length of pterostigma behind level of apex of pterostigma; vein r and vein 1-M distinctly curved; vein 1-SR approximately as long as parastigmal vein; basal third of vein 3-CU1 tubular, remainder largely nebulous, apically distinctly curved.

*Legs.* Hind coxa rather robust, largely spaced annularly striate dorsally, basal part rugose, outer side distinctly compressed medially and coarsely rugose (Figs 259, 260); hind femur strongly swollen, finely transversely striate, evenly distributed with small punctures, each puncture bearing a short whitish seta, hind femur with 3 large ventral teeth, basal one obtuse and another two teeth acute, with small tubercles in interspaces (Fig. 262); hind tibia 1.4 times as long as hind femur, basal narrow part about 1.2 times as long as widened part; outer side of hind tibia distinctly obliquely carinate and ventral carina rather stout, narrow part of inner side coarsely obliquely striate, widened part of inner side basally distinctly steeply depressed, medially distinctly convex and granulate, apically densely bristly setose (Fig. 262); basitarsus rather robust, ventral length about 6.0 times as long as its apical width.

*Metasoma.* First tergite cylindrical, about 8.3 times as long as its maximum width, 2.2 times second tergite and 0.9 times remainder of tergites, basal 0.1 rugose and apical 0.05 smooth, remainder largely finely transversely striate; basal third of second tergite rugose, remainder polished smooth; remainder of tergites densely transversely aciculate; pygidial area with 2 distinct projections, pygidial impression setose and somewhat reverse U-shaped (Fig. 261); ovipositor sheath about 1.5 times as long as body.

*Colour.* Body colour varies from reddish brown to dark brown; scape, pedicel and first-third flagellomeres brown, remainder of antenna darker; mandibles except apex and clypeus yellow; temple along eye with ivory wide streak; frons, coronal area, basal third of second tergite orange; mesosoma (except neck and lateral part of mesoscutum dark brown) and basal 0.2 of first tergite largely red brown; fore wing membrane largely pale brownish, pterostigma dark brown; apical parts of both second and third tergites with golden yellow patch; ovipositor sheath completely blackish.

###### Distribution.

China (Hainan).

###### Etymology.

The name is derived from “angulatus” (Latin for angled) because of the angled temples.

##### Parastephanellus brevicoxalis sp. n.

urn:lsid:zoobank.org:act:453F407D-2852-4A59-9131-43EE66E19F6E

http://species-id.net/wiki/Parastephanellus_brevicoxalis

Figs 265 277

###### Type material.

Holotype, ♀ (ZJUH): CHINA: Zhejiang, Wuyanling, 29. vii. 2005, Peng Xu, No. 200605074.

###### Diagnosis.

Frons densely foveolate-rugose (Fig. 271); temple dark brown and with ivory streak along eyes distinctly contrasting with surroundings (Fig. 270); pronotum robust and weakly rugose (Figs 266, 267); propodeum coarsely and irregularly foveolate (Fig. 268); pterostigma comparatively long and about 5.5 times as long as wide (Fig. 265); first discal cell of fore wing comparatively narrow and its length about 3.1 times as long as vein 1-M (Fig. 265); hind coxa comparatively short and wide in lateral view (Fig. 273); basal narrow part of outer side of hind tibia distinctly carinate; apical large tooth of hind femur comparatively narrow and acute (Figs 275, 276); middle basitarsus of female about 6 times as long as its medial with; first tergite of female comparatively robust; ovipositor sheath about 1.4 times as long as body; pygidial process of female medium-sized (Fig. 274).

###### Description.

Holotype, female, length of body 16.2 mm, of fore wing 9.9 mm, and of ovipositor sheath 22.9 mm.

*Head.* Flagellum with 33 flagellomeres; frons coarsely reticulate-rugose (Fig. 271); three anterior coronal teeth acute, both posterior ones wide and arcuate, sculpture on coronal area from rugose anteriorly to longitudinally short carinate; behind level of coronal area with five strong, curved carinae, followed by transversely rugose area, rugae coarse anteriorly, finer laterally near eye and posteriorly, striae posteriorly weaker and narrowly reaching to occipital carina (Fig. 269); temple smooth and shiny, relatively broad, gena round (Fig. 270).

*Mesosoma.* Neck (Figs 266, 267) short and robust, anteriorly distinctly emarginate, medio-posteriorly smooth, and with pairs of oblique lateral carinae, neck at much lower level than remainder of pronotum; pronotal fold and concavity absent; middle pronotum steeply elevated and subvertical to neck, largely transversely striate; posterior pronotum not differentiated from middle part, weakly striate dorsally and sparsely with punctures laterally and more or less smooth apically (Fig. 266), pronotal lobe with oblique carinae; lateral oblique groove of pronotum narrow and indistinct, ventral area below it distinctly obliquely striate (Fig. 267); propleuron relatively wide, coriaceous and microreticulate; prosternum densely transversely striate and pubescent; mesoscutum foveolate, anterior 0.2 and area between foveolae striate, latero-posteriorly somewhat rugose; notauli and median groove distinct and formed by some foveolae or crenulae; axilla irregularly densely striate, rugose-foveolate near scutellum; scutellum (Fig. 268) laterally densely foveolate and medially rugulose; mesopleuron rather robust, dorsally flat and largely smooth, convex ventral part shallowly rugose and pubescent, anteriorly pubescence denser and rugae coarser than posteriorly; mesosternum largely striate and anterior part pubescent; convex part of metapleuron coarsely reticulate-rugose and sparsely setose, ventral part below it finely striate; propodeum irregularly foveolate, foveolae changing from circular to suboval, area in between and inside foveolae striate, foveolae laterally and apically somewhat coalescent and reticulate (Fig. 268).

*Wings.* Fore wing (Fig. 265): vein 1-M 1.25 times as long as vein 1-SR and 0.9 times vein m-cu; vein cu-a slightly postfurcal and subvertical; vein 2-SR 1.25 times as long as vein r; vein r ends 0.3 times length of pterostigma behind level of apex of pterostigma; vein r and vein 1-M distinctly curved; vein 1-SR 1.4 times as long as parastigmal vein; vein 3-CU1 basal 0.2 tubular, remainder largely nebulous, apically distinctly curved.

*Legs.* Hind coxa (Figs 272, 273) robust, antero-dorsally rugose, anterior 0.6 of outer side distinctly compressed and sculpture changing from rugose to microreticulate, posterior part of hind coxa coarsely transversely striate; hind femur (Fig. 275) strongly swollen, densely finely aciculate, ventrally with 2 large teeth and some denticles in between, basal one third part having 2 obtuse teeth much smaller; hind tibia (Figs 275, 276) about 1.2 times as long as hind femur, basal narrow part about 1.4 times as long as widened part, outer side of hind tibia distinctly obliquely carinate, narrow part of inner side coriaceous, widened part of inner side distinctly depressed basally and densely bristly setose apically; basitarsus rather robust, ventral length about 3.8 times as long as its apical width.

*Metasoma.* First tergite 4.6 times as long as its maximum width, 1.7 times second tergite and 0.6 times remainder of tergites, densely coarsely and rather regularly transversely striate, basal 0.1 rugose and with 2 distinct, short longitudinal carinae, apically narrowly smooth; basal 0.2 of second tergite with several short longitudinal carinae, remainder of tergite smooth; remainder of tergites densely finely microaciculate; pygidial area with two distinct projections, pygidial impression setose and somewhat reverse V-shaped (Fig. 274); ovipositor sheath (Fig. 276) about 1.4 times as long as body.

*Colour.* Largely black; head tricoloured: coronal teeth, vertex medio-longitudinally and narrow area of vertex behind eyes dark brown; frons and most of vertex reddish; gena narrowly along eye margin yellowish; propleuron largely yellowish; middle basitarsus with yellowish tint; fore leg, hind trochanter, hind tibia and basal 0.2 of second tergite dark reddish brown; wing membrane subhyaline; pterostigma and wing venation dark brown.

*Male.* Unknown.

###### Distribution.

China (Zhejiang).

###### Etymology.

The name is derived from “brevis” (Latin for short) and “coxa” (Latin for hip) because of the short hind coxa.

##### Parastephanellus brevistigma

Enderlein, 1913

http://species-id.net/wiki/Parastephanellus_brevistigma

Figs 278 [Fig F37] 298

Parastephanellus brevistigma Enderlein 1913: 203; Brues 1918: 100; Elliott 1922: 747, 756; Chao 1964: 380, 387; Belokobylskij 1995: 22; Aguiar 2004: 65.
Parastephanellus politus Chao 1964 (notElliott 1928): 380, 387. syn. n.
Parastephanellus austrochinensis Belokobylskij 1995: 22; Aguiar 2004: 65. syn. n.

###### Type material.

Holotype of *Parastephanellus brevistigma*,♂ (SDEI): “ Formosa, Kankau (Koshun), H. Sauter, v.1912”, “22.iv.”, “*Parastephanellus brevistigma* Type Enderl. ♂ Dr. Enderlein det. 1913”, “Holotypus”, “Coll. DEI Eberswalde”, “OSUC 0021613”.

###### Other material.

7 ♂ (ZJUH): 1 ♂, CHINA: Guangxi, Longzhou Nonggang, 1982.v.21, Jun-hua He, No. 821669; 6♂, CHINA: Guangxi, Longzhou, 1982.v.22, No. 822167, No. 822168, No. 822169, No. 822147, No. 822148, No. 822149.

###### Diagnosis.

Head in dorsal view orange brown and sparsely striate (Figs 284, 291); frons and temple yellowish; frons transversely rugose (Figs 286, 293); temple smooth and shiny (Figs 285, 292); pronotum comparatively short and its lateral length 0.8–0.9 times maximum width, in dorsal view superficially sculptured and shiny (Figs 279, 280, 288, 289); propodeum shallowly and sparsely foveolate and coriaceous interspaces mostly wider than diameter of punctures (Figs 281, 290); pterostigma distinctly widened and comparatively short, 4–5 times as long as wide (Figs 278, 287); outer side of hind tibia finely carinate (Figs 294, 295).

###### Description.

Holotype, male, length of fore wing 4.1 mm, and of incomplete body 2.8 mm (metasoma lost).

*Head.* Flagellum with 23 flagellomeres; coronal teeth large and distinctly carinate; frons coarsely striate (Fig. 286); vertex with several spaced striae, area near lateral ocelli rugulose (Fig. 284); temple smooth and shiny (Fig. 285), evenly convex behind eyes.

*Mesosoma.* Pronotum (Figs 279, 280) largely smooth and weakly sculptured; neck short and laterally with several pairs of carinae; posterior pronotum rugose, somewhat coriaceous; scutellum medially rugulose and laterally with several shallow foveolae (Fig. 281); propodeum (Fig. 281) and metapleuron coarsely shallowly foveolate.

*Wings.* Fore wing (Fig. 278): vein 1-M 1.5 times as long as vein 1-SR and 1.1 times vein m-cu; vein cu-a slightly antifurcal and subvertical; vein 2-SR 1.7 times as long as vein r; pterostigma distinctly widened and comparatively short, 4.5 times as long as wide; vein r ends 0.4 times length of pterostigma behind level of apex of pterostigma; vein 1-SR 1.2 times as long as parastigmal vein; basal 0.2 of vein 3-CU1 tubular, remainder largely nebulous, apically distinctly curved.

*Legs.* Holotype has only hind coxa left on the legs, legs described after a male from Guangxi (Longzhou). Hind coxa (Figs 282, 283, 297) largely striate, medial part of outer side compressed and more or less rugulose; hind femur (Figs 294, 295) strongly swollen, finely aciculate and with 2 large ventral teeth; hind tibia 1.2 times as long as hind femur, basal narrow part 1.7 times as long as widened part; outer side of hind tibia coriaceous and ventrally with some spaced oblique carinae; widened part of inner side distinctly depressed basally and densely setose apically; ventral length of hind basitarsus about 3.5 times as long as its apical width.

*Metasoma.* Metasoma of holotype lost, described after a male from Guangxi (Longzhou). First tergite coarsely transversely striate, 4.8 times as long as its width, 1.9 times as second tergite and 0.9 as rest of tergite; second tergite basally with weak rugose, rest of metasoma largely smooth, somewhat microreticulate; pygidial process distinct and tubular apically (Fig. 298).

*Colour.* Body varies from yellowish brown to dark brown; frons and temple yellowish (with a paler ivory streak along eye) (Figs 285, 286, 292, 293); vertex and pronotum orange brown; scutellum and propodeum dark brownish.

###### Distribution.

China (Guangxi, Taiwan).

##### Parastephanellus matsumotoi

van Achterberg, 2006

http://species-id.net/wiki/Parastephanellus_matsumotoi

Figs 299 [Fig F40] [Fig F41] [Fig F42] [Fig F43] 337

Parastephanellus matsumotoi van Achterberg 2006: 219–221.

###### Type material.

Paratypes, 2 ♀ (RMNH): “JPN [= Japan]/Kyushu [Kagoshima] Makizono t. Takachiho, 29.vii.2005, 31°52'33"N/130°53'29"E, R. Matsumoto”, “♀ *Parastephanellus matsumotoi* sp. nov., C. van Achterberg, 2006, PARATYPE”; id., but 31°53'25"N, 130°49'05"E.

###### Other material.

5 ♀+7 ♂ (ZJUH): 1 ♀, CHINA: Shaanxi, Qinling, Mt. Tiantai, 1999.ix.3, Yun Ma, No. 991151; 1♀, CHINA: Henan, Songxian, Mt. Baiyun, 1996.vii.19, Ping Cai, No. 972997; 3♀, CHINA: Henan, Luoshan, Lingshan, 2000.v.22, Ping Cai, No. 200101896; id., but No. 200101873, No. 200101882; 6 ♂, id., but No. 200101876, No. 200101884, No. 200101893, No. 200101906, No. 200101908, No. 200101911; 1 ♂, CHINA: Zhejiang Suichang, 1982.vi.28–vii.1, Han-lin Chen, No. 924696; 1♀ (HNHM), “Formosa, Sauter”, “Polisha, x.1909”.

###### Diagnosis.

Frons largely dark brown and reticulate-rugose (Figs 305, 318, 331); temples moderately angulate to rather rounded in dorsal view (Figs 303, 316, 329); distance from dorsally widened pale streak on temple to occipital carina in dorsal view more than width of streak (Figs 303, 316, 329); pronotum dark brown or blackish, transversely striate-rugose (Figs 300, 301, 313, 314, 326, 327); hind femur matt or with satin sheen, longer and largely coriaceous (Figs 309, 322, 335).

###### Description.

Redescribed from a female from Henan, length of body 13.4 mm, of fore wing 7.1 mm, and of ovipositor sheath 19.2 mm.

*Head.* Flagellum with 31 flagellomeres; frons coarsely reticulate-rugose (Fig. 331); three anterior coronal teeth large and acute, both posterior ones wider and arcuate; vertex flattened, largely rugose or rugulose, posteriorly transversely striate (Fig. 329); temple smooth and shiny (Fig. 330), rounded convex in dorsal view.

*Mesosoma.* Neck short and transversely carinate, anteriorly distinctly emarginated, posteriorly with transverse groove in dorsal view; pronotum largely striate or rugulose, posteriorly narrowly smooth (Figs 326, 327); propleuron largely coriaceous; mesopleuron coriaceous and with some shallow foveolae, anteriorly densely setose; scutellum aciculate medially and with round punctures laterally (Fig. 328); propodeum spaced foveolate and with coriaceous interspaces (Fig. 328); metapleuron coarsely reticulate-foveolate, convex medially.

*Wings.* Fore wing (Fig. 325): vein 1-M 1.4 times as long as vein 1-SR and 1.1 times vein m-cu; vein cu-a slightly antefurcal and subvertical; vein 2-SR 1.3 times as long as vein r; vein r ends 0.4 times length of pterostigma behind level of apex of pterostigma; vein 1-M and vein r distinctly curved; vein 1-SR 1.4 times as long as parastigmal vein; vein 3-CU1 largely nebulous, only basal 0.2 tubular.

*Legs.* Hind coxa robust, largely coarsely rugose; hind femur rugulose, ventrally with 2 large teeth and one smaller basal tooth (Fig. 335); hind tibia (Figs 335, 336) largely coriaceous and ventrally distinctly oblique carinate, 1.3 times as long as hind femur, basal narrow part of hind tibia about 1.2 times as long as widened part; ventral length of hind basitarsus 5.2 times as long as its width.

*Metasoma.* First tergite densely transversely striate, 7.5 times as long as its maximum width, 2.3 times second tergite and 0.9 times remainder of metasoma; second tergite basally rugose; remainder of metasoma largely smooth and shiny; pygidial area differentiated and with pair of truncate horns apically, pygidial impression distinct and reversed V-shaped (Fig. 334); ovipositor broken; length of ovipositor sheath about 1.4 times as long as body.

*Colour.* Largely blackish or dark brown; temple with yellowish streak along eye.

*Variation.* Female: length of body 7.5–16.4 mm, of fore wing 5.2–9 mm, and of ovipositor sheath 16.9–21.2 mm; frons completely dark brown or black or with orange or reddish patch (Figs 305, 318, 331); temple colour varied from reddish to blackish and with yellowish or ivory streak along eye (Figs 304, 317, 330). Male: similar to female, length of body 7.5–13 mm, and of fore wing 5–9 mm.

###### Distribution.

China (Shaanxi, Henan, Zhejiang); Japan (Kyushu).

###### Note.

This species is new record from China.

##### Parastephanellus zhejiangensis sp. n.

urn:lsid:zoobank.org:act:EACC7E60-6ECE-4E7B-89AD-150DF5901347

http://species-id.net/wiki/Parastephanellus_zhejiangensis

Figs 338 350

###### Type material.

Holotype, ♀ (ZJUH): CHINA: Zhejiang, Kaihua, Mt. Gutian, 2005.vii.1–3, Xue-xin Chen, No. 200604294.

###### Diagnosis.

Temple completely yellowish, yellowish streak stretching to vertex and reaching occipital carina (Figs 352, 353); pronotum rugose-striate in dorsal view (Fig. 339); propodeum densely foveolate and interspaces small (Fig. 341); vein 1-M of fore wing 2.2 times as long as vein 1-SR and 1.3 times as vein m-cu (Fig. 338); hind femur less swollen; apical large tooth of hind femur comparatively wide and obtuse (Fig. 348); hind tibia yellowish brown and ventrally with a few weak carinae, basal narrow part of outer side with striae (Figs 348, 349); ovipositor sheath about as long as body; pygidial process of female rounded apically (Fig. 347).

###### Description.

Holotype, female, length of body 9.8 mm, of fore wing 5.8 mm, and of ovipositor sheath 10.2 mm.

*Head.* Antenna with flagellomeres partly missing; frons transversely striate-rugose (Fig. 344); three anterior coronal teeth acute, both posterior ones wide and arcuate; sculpture on vertex varied from rugose to striate-rugose (Fig. 342); temple smooth and shiny (Fig. 343), distinctly roundedly convex behind eyes.

*Mesosoma.* Pronotum (Figs 339, 340) robust, dorsally largely coarsely carinate or striate and laterally rugulose; neck short and transversely impressed posteriorly; pronotal fold and concavity absent; middle pronotum gradually elevated connected with posterior pronotum; mesoscutum anteriorly microreticulate and posteriorly foveolate; notauli and median groove distinct and formed by foveolae; scutellum (Fig. 341) coarsely coriaceous and laterally with several foveolae; mesopleuron robust, irregularly with dense and shallow foveolae; convex part of metapleuron coarsely reticulate-rugose; propodeum densely and coarsely foveolate (Fig. 341).

*Wings.* Fore wing (Fig. 338): vein 1-M 2.2 times as long as vein 1-SR and 1.3 times vein m-cu; vein cu-a slightly antifurcal and subvertical; vein 2-SR 2.6 times as long as vein r; vein r ends 0.3 times length of pterostigma behind level of apex of pterostigma; vein r and vein 1-M distinctly curved; vein 1-SR slightly shorter than parastigmal vein; vein 3-CU1 basal 0.2 tubular, remainder largely nebulous, apically distinctly curved.

*Legs.* Hind coxa rugose, but rugae on posterior third more regular and somewhat transverse (Figs 345, 346); hind femur densely finely aciculate, ventrally with two large obtuse teeth and some denticles in between (Figs 348, 349); hind tibia about 1.2 times as long as hind femur, basal narrow part 1.3 times as long as widened part, narrow part of inner side coriaceous (Fig. 349), widened part of inner side distinctly depressed basally and densely bristly setose apically; basitarsus rather robust, ventral length about 4.2 times as long as its apical width.

*Metasoma.* First tergite densely transversely striate, 4.2 times as long as its maximum width, 2.2 times second tergite and 0.9 times remainder of tergites; basal 0.2 of second tergite with weak rugae, remainder of tergite smooth or indistinctly microreticulate; pygidial impression indistinct (Fig. 347); ovipositor sheath (Fig. 350) about same size as body.

*Colour.* Largely blackish; frons largely orange-brown; temple and hind tibia completely yellowish; second tergite basally reddish.

*Male.* Unknown.

###### Distribution.

China (Zhejiang).

###### Etymology.

The name is derived from “Zhejiang” because the type locality is situated in Zhejiang Province (China).

#### Genus Schlettererius

Ashmead, 1900

http://species-id.net/wiki/Schlettererius

Figs 351 [Fig F48] [Fig F49] 375

Schlettererius Ashmead 1900: 150. Type species (by monotypy and original designation): *Stephanus cinctipes* Cresson, 1880.

##### Diagnosis.

Pronotum more or less rectangularly connected with rest of pronotum (Figs 352, 366); vein 1-M of fore wing distinctly curved (Figs 351, 365); vein cu-a of hind wing present as pigmented vein (Fig. 365); hind coxa with small subapical dorsal tooth (Figs 358, 372); hind tibia not distinctly narrowed and compressed basally (Figs 361, 374); hind tarsus of female with five tarsomeres (Fig. 360); sternite of first metasomal tergite differentiated from its tergite, first tergite 2.4–4.6 times as long as its apical width, not cylindrical, about as long as second tergite (Figs 362, 363, 373, 375); second tergite sessile and smooth basally (Figs 362, 363, 375); posterior part of eighth metasomal tergite of female with pygidial process (Figs 362, 363); ovipositor sheath with ivory subapical band (Fig. 364).

##### Distribution.

Nearctic, East Palaearctic and Australian (only Tasmania: introduced for biological control of introduced Siricidae).

##### Notes.

Two species of *Schlettererius* are known worldwide, one from China and one from North America. A key to the two species follows:

##### Key to world species of the genus Schlettererius Ashmead

**Table:** 

1	Posterior pronotum gradually elevated (Fig. 367); first-third metasomal tergites black or dark brown (Figs 373, 375); ovipositor sheath of female with subapical whitish band 3.7 times as long as apical blackish part	*Schlettererius determinatoris* Madl, 1991
–	Posterior pronotum steeply elevated (Fig. 353); first metasomal tergite posteriorly, second and third tergites yellowish brown (Figs 362, 363); ovipositor sheath of female with subapical whitish band 1.8 times as long as apical blackish part (Fig. 364)	*Schlettererius cinctipes* Cresson, 1880

##### Schlettererius determinatorius

Madl, 1991

http://species-id.net/wiki/Schlettererius_determinatorius

Figs 365 375

Schlettererius determinatoris Madl 1991: 119–120; Belokobylskij 1995: 18; van Achterberg 2002: 198; Aguiar 2004: 75.

###### Type material.

Holotype, ♀ (HNHM), “KOREA, Prov. North Pyongan, Mount Myohyang-san”, “17.07.1982, No.815, Leg. Dr. L. Forro & Dr. L. Ronkay”, “Holotypus ♀ *Schlettererius determinatorius* n. sp. MADL, 1990”, “OSUC 0021616”.

###### Other material.

1 ♂ (ZJUH): CHINA: Shaanxi, Liuba, Mt. Zibai, 1632 m, 4.viii.2004, Hong-ying Zhang, No. 20047080.

###### Diagnosis.

Posterior half of pronotum comparatively low and dorso-posteriorly finely transversely rugose (Figs 366, 367); first subdiscal cell of fore wing comparatively robust and 2.0–2.5 times longer than wide (Fig. 365); first-third metasomal tergites black or dark brown (Fig. 375); first tergite irregularly coarsely transversely rugose (Figs 373, 375).

###### Description.

Redescribed after the male from Shaanxi (Liuba), length of body 9.8 mm, and of fore wing 6.5 mm.

*Head.* Flagellum with 27 flagellomeres; frons (Fig. 371) coarsely transversely rugose; three anterior coronal teeth large and acute, both posterior ones arcuate and lamelliform, with two small lobe-shaped carinae on each side in front of both posterior ocelli; behind level of coronal area having four curved, progressively smaller carinae followed by rugose area, rugae finer medio-dorsally and more or less reticulate laterally, posteriorly narrowly reaching occipital carina (Fig. 369); temple smooth and shiny except for some very small punctures ventrally, somewhat rounded in dorsal view (Fig. 370).

*Mesosoma.* Neck (Figs 366, 367) short and robust, irregularly carinate; middle pronotum largely smooth and with a distinct, somewhat sinuate carina posteriorly; posterior pronotum medio-dorsally smooth and laterally coarsely rugose; lateral oblique groove of pronotum rather narrow and smooth, ventral area below it obliquely striate; propleuron coriaceous; mesonotum (Fig. 366) irregularly foveolate and area between smooth; notauli and median groove indistinct; scutellum (Fig. 368) largely smooth medially, foveolate laterally and marginally; axillae rugose-foveolate; mesopleuron rather robust and distinctly convex, convex part rugose-reticulate and covered with whitish setosity, flat dorsal part sparsely carinate; mesosternum anteriorly rugose and posteriorly sparsely punctate; medially convex part of metapleuron reticulate-rugose and with short whitish setosity, antero-ventrally weakly crenulate, with dorsal anterior depression rather deep and ventral one less impressed; propodeum (Fig. 368) densely and irregularly rugose.

*Wings.* Fore wing (Fig. 365): vein 1-M distinctly curved, 2.4 times as long as vein 1-SR; vein r ends before level of apex of pterostigma; first subdiscal cell robust, twice as long as its maximum width. Hind wing (Fig. 365): vein cu-a largely pigmented.

*Legs.* Hind coxa (Fig. 372) robust, largely coarsely striate-rugose, with a small obtuse subapical dorsal tooth (but absent in other coxa); hind femur (Fig. 374) slender, granulate and covered with whitish, sparse setae, apically more or less strigate, ventrally with 3 acute teeth (the anterior one much smaller) and some denticles in between; hind tibia (Fig. 374) 1.1 times as long as hind femur, basal narrow part of hind tibia 0.5 times long as widened part, widened part ventrally distinctly obliquely carinate; length of hind basitarsus 3.7 times as long as its width.

*Metasoma.* First tergite (Figs 373, 375) robust, subcylindrical, 4.6 times as long as its maximum width, coarsely rugose, posteriorly more or less striate, laterally with whitish setosity; second tergite about as long as first tergite, smooth and finely sparsely punctate; remainder of tergites smooth and shiny; pygidial process distinct and tubular apically (Fig. 375).

*Colour.* Body colour varies from light brown to blackish; malar space yellowish; antenna, fore and middle legs and wing membrane light brown; frons, vertex, pronotum and first tergite dark brown; mesosoma, hind coxae blackish; temples, metasoma (except first tergite) and hind legs (except hind coxae) brown.

*Female.* See Madl (1991) for detailed description.

###### Distribution.

Palaearctic China (Shaanxi); North Korea.

###### Note.

For redescription in Chinese, see Hong and Xu (2011).

#### Genus Stephanus

Jurine (in Panzer), 1801

http://species-id.net/wiki/Stephanus

Figs 376 [Fig F52] [Fig F53] 396

Stephanus Jurine (in Panzer) 1801: 76, Fig. 13. Type species (by monotypy): *Stephanus coronatus* Jurine (in Panzer), 1801 (= *Ichneumon serrator* Fabricius, 1798).

##### Diagnosis.

Temple without yellowish streak along eye, at most with yellow patch (Figs 381, 392); first subdiscal cell of fore wing comparatively narrow basally, and vein 1-SR differentiated with first discal cell present because of presence of vein 1-SR+M (Figs 376, 387); hind wing with vein M+Cu, but without trace of vein cu-a (Fig. 387); hind femur (Figs 383, 394) distinctly slender and elongate, coarsely striate, ventrally with 2–3 large teeth, rarely with 4 teeth; inner side of hind tibia only with a short narrow oblique groove below a small convexity; hind tarsus of female with five tarsomeres; ovipositor sheath completely blackish (Fig. 386).

##### Distribution.

Palaearctic and Oriental.

##### Notes.

Before this study, 5 species in the genus *Stephanus* were known worldwide, of which 2 species were known from China. In this paper, these two species of the genus are listed from China. A Key to species of *Stephanus* from China follows:

##### Key to species of the genus Stephanus from China

**Table:** 

1	First metasomal tergite comparatively slender, about 9 times as long as its maximum width (Figs 384, 385); hind femur with two large ventral teeth (Fig. 383); small part of vein M+CU of hind wing pigmented	*Schlettererius bidentatus* van Achterberg & Yang, 2004
–	First metasomal tergite often comparatively robust, 3–5.7 times as long as its maximum width (Figs 395, 396); hind femur with three large ventral teeth (Fig. 394), rarely with 4 teeth; large part of vein M+CU of hind wing pigmented (Fig. 387)	*Schlettererius tridentatus* van Achterberg & Yang, 2004

##### Stephanus bidentatus

van Achterberg & Yang, 2004

http://species-id.net/wiki/Stephanus_bidentatus

Figs 376 386

Stephanus bidentatus van Achterberg and Yang 2004: 104–106.

###### Type material.

Holotype, ♀ (CAFB), “CHINA: Henan, Longyuwan, Lianchuan, 700m, on trunk of *Quercus* tree with Cerambycidae larvae, 13.vii.1996, Zhong-qi Yang”.

###### Other material.

1 ♀ (SCAU): CHINA: Henan, Neixiang County, Baotianman, 13–15.vii.1998, Yun Ma, No. 987231.

###### Diagnosis.

Pronotum without distinct pronotal fold medially (Figs 377, 378); small part of vein M+CU of hind wing pigmented; scutellum densely rugose (Fig. 379); hind femur with 2 large ventral teeth (Fig. 383); first metasomal tergite more slender, about 9 times as long as its maximum width (Figs 384, 385).

###### Description.

Redescribed after a female from Henan (Baotianman), length of body 17.7 mm, of fore wing 11.9 mm, and of ovipositor sheath 28 mm.

*Head.* Flagellum with 25 flagellomeres; first flagellomere 3.9 times as long as wide, and 0.7 times as long as second flagellomere; frons (Fig. 382) coarsely reticulate and densely setose; three anterior lobe-shaped coronal teeth of head large, hardly larger than both posterior ones; vertex with four curved, progressively smaller carinae behind level of both posterior coronal lobes, remainder of vertex rather coarsely reticulate-rugose, sculpture becoming finer posteriorly and narrowly reaching occipital carina (Fig. 380); temples smooth except for some small punctures bearing setosity ventrally (Fig. 381), shiny and rather angulate in dorsal view.

*Mesosoma.* Neck (Fig. 377) comparatively short and robust, anteriorly shallowly emarginate, medio-dorsally smooth and laterally with several coarse and irregular carinae which curved backwards, postero-dorsally at lower level than middle pronotum, with a distinct cavity below pronotal fold; pronotal fold distinctly developed laterally and absent medially; middle pronotum robust, coarsely and irregular rugose, not distinctly differentiated from posterior pronotum (Figs 377, 378); lateral oblique groove of pronotum distinct and rather wide, impression largely carinate and ventral area below it coarsely rugose (Fig. 378); postero-laterally narrowly short setose; posterior part of pronotum dorsally coarsely transversely rugose and somewhat smooth and shiny posteriorly; mesoscutum laterally coarsely rugose-reticulate, medially densely foveolate-rugose, notauli and median groove rather distinct; scutellum completely coarsely reticulate-rugose; axillae densely foveolate (Fig. 379); propleuron coarsely punctate; convex part of mesopleuron reticulate-foveolate and covered with whitish and rather sparse setosity; dorsal part densely rugose and setose; mesosternum with spaced punctures; medially metapleuron strongly convex, coarsely foveolate-reticulate and with short whitish setosity, antero-ventrally weakly crenulate and with dorsal anterior depression rather deep and ventral one less impressed; propodeum (Fig. 379) densely and irregularly rugose-foveolate.

*Wings.* Fore wing (Fig. 376): vein 1-M 3.6 times as long as vein 1-SR and nearly straight. Hind wing: vein M+CU only partly pigmented and after middle of wing.

*Legs.* Hind coxa moderately slender, subparallel-sized, largely coarsely rugose, but posterior third striate; hind femur (Fig. 383) slender, largely finely transversely striate, with two acute, large teeth and some denticles in between, laterally with spaced, small punctures and each bearing a whitish seta; basal narrow part of hind tibia 1.3 times long as widened part, parallel-sided and with ventral carina; outer side of widened part of hind tibia coriaceous, with small sparse punctures bearing whitish setae (Fig. 383); inner side flattened, sparsely granulate, apically with densely bristly setose area; hind basitarsus parallel-sided, basally hardly curved, ventral length 8.3 times its width.

*Metasoma.* First tergite (Figs 384, 385) subcylindrical, 8.9 times as long as its apical width, basally coarsely reticulate-rugose, remainder irregularly and densely rugose (Fig. 384); second tergite basally rugose, remainder mainly smooth; pygidial area not lamelliform posteriorly, pygidial impression reverse V-shaped; length of ovipositor sheath 1.5 times as long as length of body.

*Colour.* Blackish or dark brown; face brownish; malar space yellowish, distinctly contrasting to dark colour of temple and vertex; fore wing membrane largely pale brownish; tibiae, tarsi brownish, and hind trochantellus pale brownish; ovipositor sheath completely blackish (Fig. 386).

###### Biology.

A parasitoid of Cerambycidae larvae in *Quercus* sp., and probably in other deciduous trees (van Achterberg and Yang 2004).

###### Distribution.

Palaearctic China (Henan).

###### Notes.

The name refers to the two ventral teeth of the hind femur. This species is unique in the Stephanidae by the combination of the hind tarsus with five tarsomeres in female and the hind femur with two large ventral teeth (van Achterberg and Yang 2004).

##### Stephanus tridentatus

van Achterberg & Yang, 2004

http://species-id.net/wiki/Stephanus_tridentatus

Figs 387 396

Stephanus tridentatus van Achterberg and Yang 2004: 106–109.

###### Type material.

Holotype, ♀ (CAFB), “CHINA: Henan, Longyuwan, Luanchuan, 700 m, ovipositing on Cerambycidae larvae in Qishu tree, 10.vii.1996, Zhong-qi Yang”. Paratypes (18 ♀ + 2 ♂: CAFB; 1 ♀: RMNH): 1 ♀, “CHINA: Henan Longyuwan, Luanchuan, ovipositing on Buprestidae larvae in *Ulmus* tree, 10.vii.1996, Zhong-qi Yang”; 5 ♀ + 2 ♂, “CHINA: Shaanxi, Louguantai, 9.ix.1991, ovipositing on Cerambycidae larvae in *Quercus* tree, Zhong-qi Yang”; 1 ♀ (RMNH), id.; 3 ♀, id., but 10.ix.1991; 3 ♀, id., but 11.ix.1991; 3 ♀, id., but 12.ix.1991; 1 ♀, id., but 13.ix.1991; 2 ♀, id., but 15.ix.1991.

###### Other material.

2 ♀ + 1 ♂ (ZJUH): 2 ♀, CHINA: Shaanxi, Mt. Xinjia, 28–30. vi.1992, No. 200011622; No. 200011617; 1 ♂, CHINA: Henan, Jiyuan, 7.vi.2000, Ping Cai, No. 200102040.

###### Diagnosis.

Pronotum (Fig. 388) with a coarse to fine pronotal fold medially; scutellum (Fig. 390) coarsely foveolate and somewhat smooth medially; large part of vein M+CU of hind wing pigmented (Fig. 387); hind femur (Fig. 394) with 3 large ventral teeth, rarely with 4 teeth (van Achterberg and Yang 2004); first metasomal tergite (Figs 395, 396) often comparatively robust and 3–5.7 times as long as its maximum width.

###### Description.

Redescribed after a female from Shaanxi (Mt. Xinjia), length of body 13.8 mm, of fore wing 10.8 mm, and of ovipositor sheath 18.0 mm.

*Head.* Flagellum with 30 flagellomeres; first flagellomere moderately slender, 3.4 times as long as wide, and 0.8 times as long as second flagellomere; frons (Fig. 393) coarsely reticulate-rugose; three anterior lobe-shaped coronal teeth of head large; both posterior ones connected and ear-like, behind them with two distinct lobes laterally and four curved, progressively smaller carinae; medio-dorsally remainder of vertex rather finely transversely striate, laterally and including area behind posterior ocelli reticulate, sculpture becoming finer posteriorly and narrowly reaching occipital carina (Fig. 391); temples smooth except for some punctures ventrally, shiny and moderately angulate in dorsal view (Fig. 392).

*Mesosoma.* Neck (Figs 388, 389) comparatively short and robust, anteriorly moderately concave, neck postero-dorsally at lower level than middle pronotum, with three pairs of distinct carinae laterally and smooth medially in front of pronotal fold, with distinct cavity under pronotal fold; pronotal fold distinctly developed and sinuate in dorsal view (Fig. 388); middle pronotum robust, with a very short median carina directly behind pronotal fold, with some irregular and rather coarse transverse carinae (Fig. 388); lateral oblique groove of pronotum distinct and rather wide, impression somewhat carinate and with wide smooth interspaces and ventral area below it coarsely rugose; postero-laterally narrowly short setose; posterior pronotum dorsally coarsely carinate-rugose; mesoscutum laterally densely coarsely rugose, medially foveolate and with rather distinct notauli and a median groove; scutellum (Fig. 390) coarsely foveolate; axillae with some spaced foveolae; propleuron coarsely punctate; convex part of mesopleuron reticulate-foveolate and covered with whitish and rather sparse setosity; dorsal part densely rugose and setose; mesosternum with coarse punctures; medially metapleuron strongly convex, coarsely foveolate-reticulate and with short whitish setosity, antero-ventrally weakly crenulate and with dorsal anterior depression and ventral depression rather deep; propodeum (Fig. 390) densely and irregularly rugose-foveolate.

*Wings.* Fore wing (Fig. 387): vein 1-M 3.3 times as long as vein 1-SR and weakly curved. Hind wing (Fig. 387): vein M+CU largely pigmented.

*Legs.* Hind coxa rather robust, subelliptical, anteriorly coarsely rugose, posteriorly striate; hind femur (Fig. 394) slender, largely finely transversely striate, with three acute, large teeth and some denticles in between, laterally sparsely setose; basal narrow part of hind tibia about 1.1 times as long as widened part, parallel-sided and with ventral carina; outer side of widened part of hind tibia coriaceous and with sparse small punctures bearing whitish setae (Fig. 394); inner side flattened, sparsely granulate, apically with densely bristly setose area; hind basitarsus parallel-sided, basally hardly curved, ventral length 6.0 times its width.

*Metasoma.* First tergite (Figs 395, 396) rather robust, subcylindrical, 5.7 times as long as its maximum width, largely irregularly and coarsely transversely rugose, basally much coarser and slightly reticulate; second tergite basally rugose, remainder smooth; pygidial area distinctly differentiated, narrowly lamelliform posteriorly, pygidial impression deep and reverse Y-shaped; length of ovipositor sheath 1.3 times as long as body.

*Colour.* Blackish or dark brown; face brownish; malar space yellowish, distinctly contrasting to temple and vertex; fore wing membrane largely pale brownish; tibiae, tarsi brownish, and hind trochantellus pale brown; ovipositor sheath completely blackish.

*Male.* Very similar to female, but smaller: length of body 11.5 mm and of fore wing 7.7 mm.

*Variation.* Female: length of body 13.8–16.9 mm, and of fore wing 10.1–12.8 mm; flagellum with 25–31 flagellomeres; middle teeth of hind femur maybe double, resulting in 4 ventral teeth in one leg and the normal 3 in the other leg (van Achterberg and Yang 2004); first tergite 4.4–5.7 times as long as its maximum width; length of ovipositor sheath 1.6–1.8 times as long as fore wing and 1.2–1.3 times as long as length of body. Male: length of fore wing 7.7–8.7 mm; flagellum with 25–27 flagellomeres.

###### Biology.

A parasitoid of Buprestidae and Cerambycidae larvae in deciduous trees (van Achterberg and Yang 2004).

###### Distribution.

Palaearctic China (Shaanxi, Henan).

###### Note.

The name refers to the three ventral teeth of the hind femur (van Achterberg and Yang 2004).

#### Genus Pseudomegischus

van Achterberg, 2002

http://species-id.net/wiki/Pseudomegischus

Pseudomegischus Achterberg 2002: 169. Type species: *Stephanus sulcifrons* Schletterer, by original designation.

##### Diagnosis.

Temple with pale yellowish streak along eye; pronotum with weak or strong transverse protuberance; hind femur comparatively robust and less elongate, largely smooth and with some punctures, ventrally with 2 large teeth; hind tibia with a ventral carina and/or with oblique striae ventro-posteriorly, inner side of hind tibia usually with wide submedial depression, occupying entire width of tibia or depression absent; hind tarsus of female with three tarsomeres; ovipositor sheath without ivory subapical band.

##### Distribution:

Philippines, Indonesia, insular Malaysia, Arabian peninsula, Africa (van Achterberg 2002).

##### Note:

This species is included in the generic key because it may occur in China. At present 4 species were known worldwide (van Achterberg 2002; Aguiar 2004), but no species are known from China.

## Supplementary Material

XML Treatment for Stephanidae

## Figures and Tables

**Plate 1.**
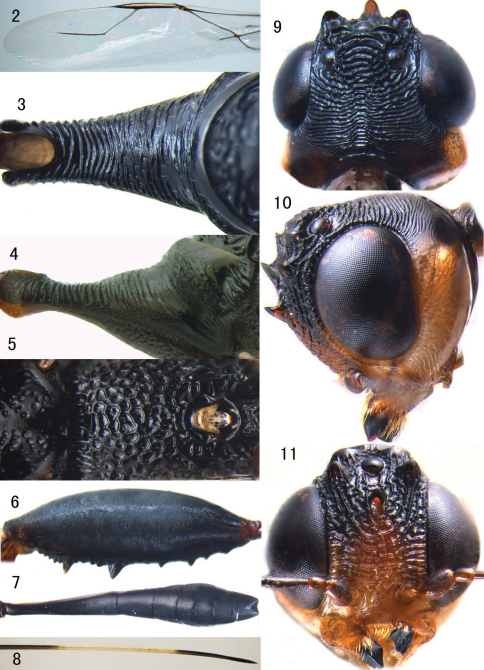
**Figures 2–11.** *Foenatopus acutistigmatus* Chao, 1964, female, Mt. Nanling, Guangdong. **2** fore wing **3** pronotum dorsal **4** pronotum lateral **5** scutellum and propodeum dorsal **6** hind femur **7** second-eighth tergites dorsal **8** ovipositor sheath **9** head dorsal **10** head lateral **11** head anterior.

**Plate 2. F3:**
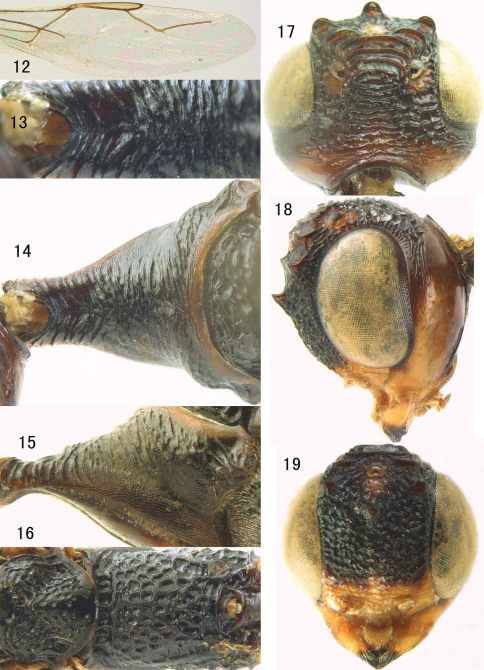
**Figures 12–19.** *Foenatopus annulitarsus* Enderlein, 1913, lectotype, female. **12** fore wing **13** neck dorsal **14** pronotum dorsal **15** pronotum lateral **16** scutellum and propodeum dorsal **17** head dorsal **18** head lateral **19** head anterior.

**Plate 3. F4:**
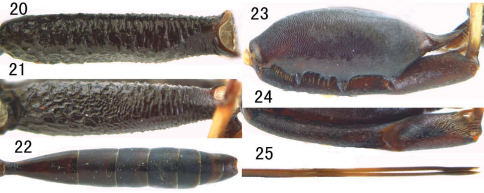
**Figures 20–25.** *Foenatopus annulitarsus* Enderlein, 1913, lectotype, female. **20** hind coxa dorsal **21** hind coxa lateral **22** second-eighth tergites dorsal **23** hind femur and outer side of hind tibia **24** inner side of hind tibia **25** ovipositor sheath.

**Plate 4. F5:**
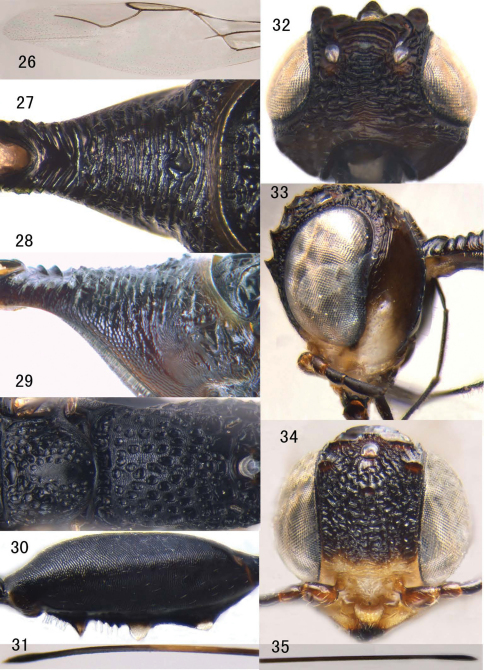
**Figures 26–35.** *Foenatopus annulitarsus* Enderlein, 1913, female, Mt. Diaoluoshan, Hainan. **26** fore wing **27** pronotum dorsal **28** pronotum lateral **29** scutellum and propodeum dorsal **30** hind femur **31** ovipositor sheath **32** head dorsal **33** head lateral **34** head anterior **35** ovipositor sheath.

**Plate 5. F6:**
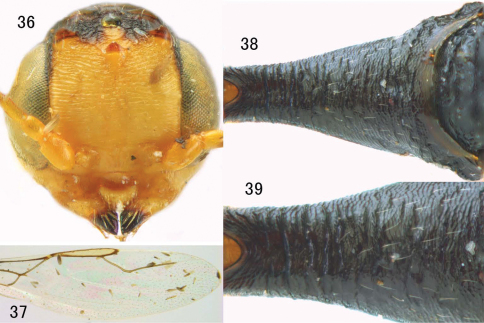
**Figures 36–39.** *Foenatopus annulitarsus* Enderlein, 1913, male, Longzhou, Guangxi. **36** head anterior **37** fore wing **38** pronotum dorsal **39** neck dorsal.

**Plate 6. F7:**
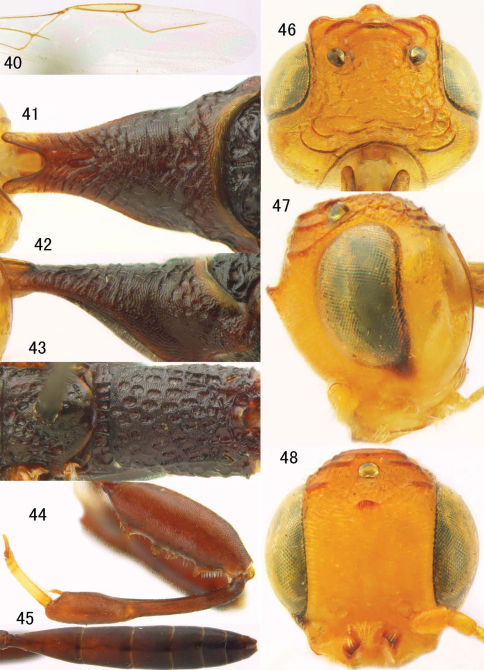
**Figures 40–48.** *Foenatopus aratifrons* Enderlein, 1913, holotype, male. **40** fore wing **41** pronotum dorsal **42** pronotum lateral **43** scutellum and propodeum dorsal **44** hind femur and outer side of hind tibia **45** second-eighth tergites dorsal **46** head dorsal **47** head lateral **48** head anterior.

**Plate 7. F8:**
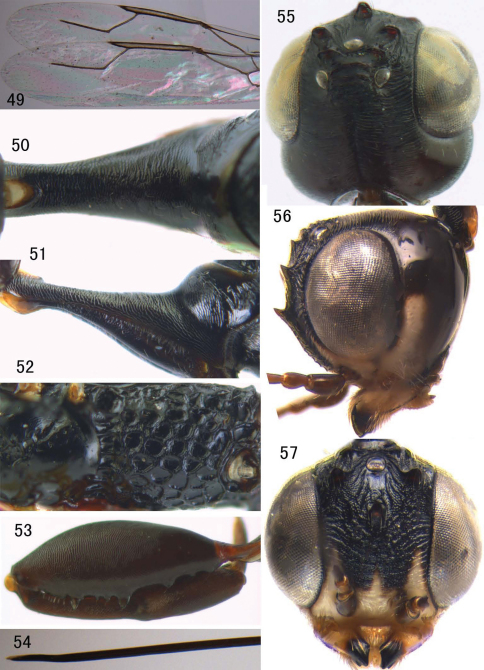
**Figures 49–57.** *Foenatopus brevimaculatus* sp. n. holotype, female. **49** fore wing **50** pronotum dorsal **51** pronotum lateral **52** scutellum and propodeum dorsal **53** hind femur and outer side of hind tibia **54** ovipositor sheath **55** head dorsal **56** head lateral **57** head anterior.

**Plate 8. F9:**
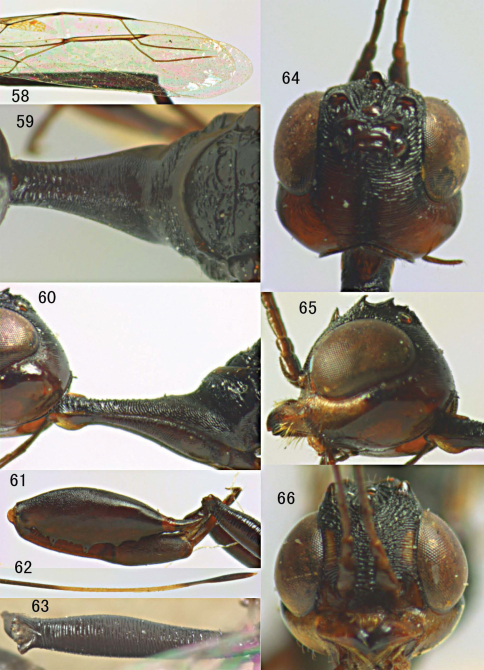
**Figures 58–66.** *Foenatopus chinensis* (Elliott, 1919), holotype, female. **58** fore wing **59** pronotum dorsal **60** pronotum lateral **61** hind femur and outer side of hind tibia **62** ovipositor sheath **63** hind coxa dorsal **64** head dorsal **65** head lateral **66** head anterior.

**Plate 9. F10:**
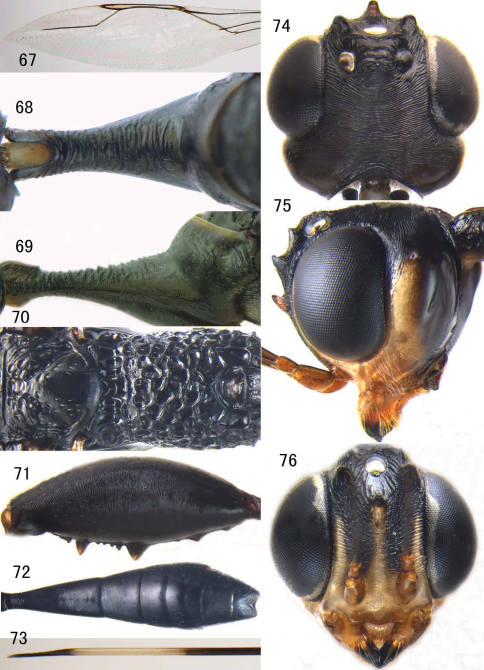
**Figures 67–76.** *Foenatopus chinensis* (Elliott, 1919), female, Guangzhou, Guangdong. **67 **fore wing **68** pronotum dorsal **69** pronotum lateral **70** scutellum and propodeum dorsal **71** hind femur **72 **second-eighth tergites dorsal **73** ovipositor sheath **74** head dorsal **75** head lateral **76** head anterior.

**Plate 10. F11:**
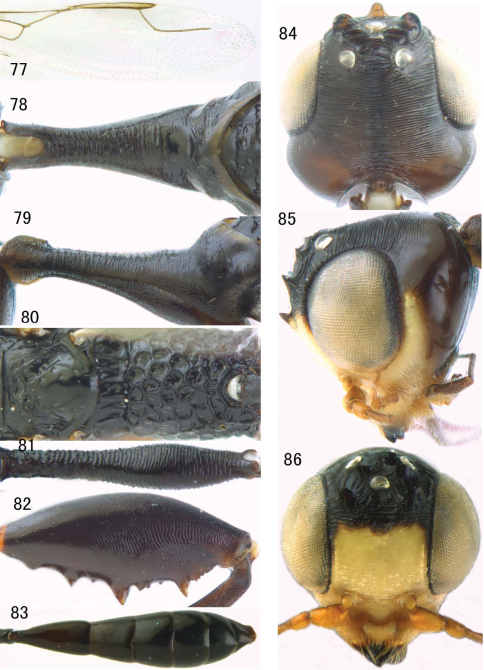
**Figures 77–86.** *Foenatopus chinensis* (Elliott, 1919), male, Zhaoqing, Guangdong. **77** fore wing **78** pronotum dorsal **79** pronotum lateral **80** scutellum and propodeum dorsal **81** hind coxa **82 **hind femur **83** second-eighth tergites dorsal **84** head dorsal **85** head lateral **86** head anterior.

**Plate 11. F12:**
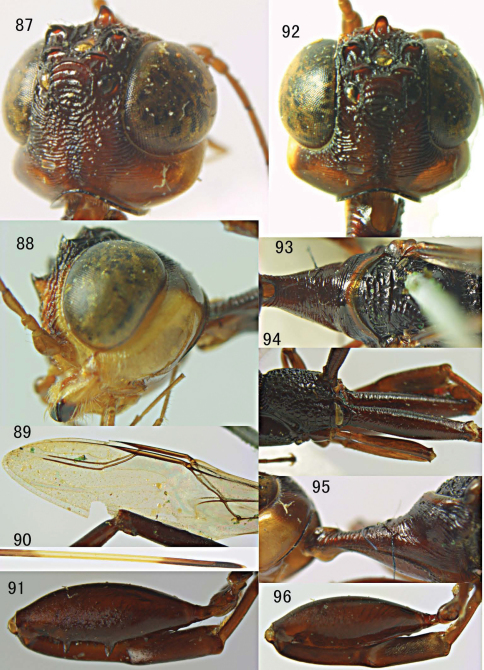
**Figures 87–96.** *Foenatopus cinctus* (Matsumura, 1912), lectotype, female. **87** head dorsal **88** head lateral **89** fore wing **90** ovipositor sheath **91** hind femur and outer side of hind tibia **92** head dorsal **93** pronotum dorsal **94** hind coxa **95** pronotum lateral **96** hind femur and inner side of hind tibia.

**Plate 12. F13:**
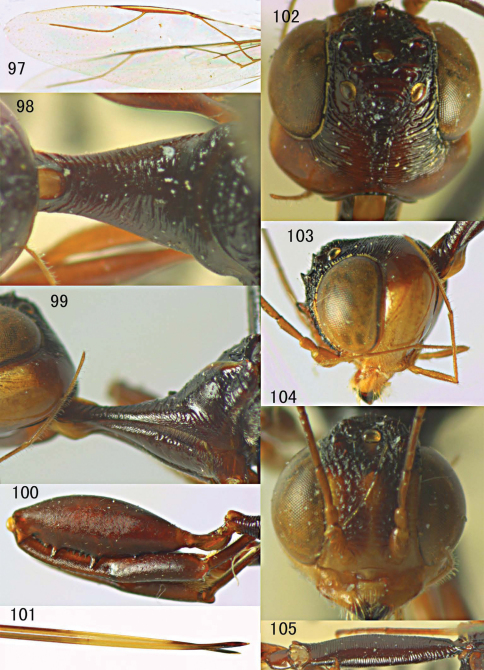
**Figures 97–105.** *Foenatopus formosanus* Enderlein, 1913, lectotype, female. **97** fore wing **98  **pronotum dorsal **99** pronotum lateral **100** hind femur and outer side of hind tibia **101** ovipositor sheath **102** head dorsal **103** head lateral **104** head anterior **105** hind coxa dorsal.

**Plate 13. F14:**
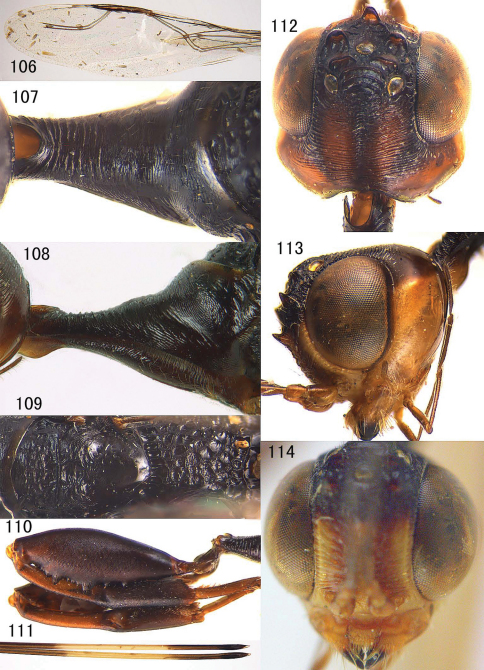
**Figures 106–114.** *Foenatopus cinctus* (Matsumura, 1912), female, Longzhou, Guangxi. **106 **fore wing **107** pronotum dorsal **108** pronotum lateral **109** scutellum and propodeum dorsal **110 **hind femur and hind tibia **111** ovipositor sheath **112** head dorsal **113** head lateral **114** head anterior.

**Plate 14. F15:**
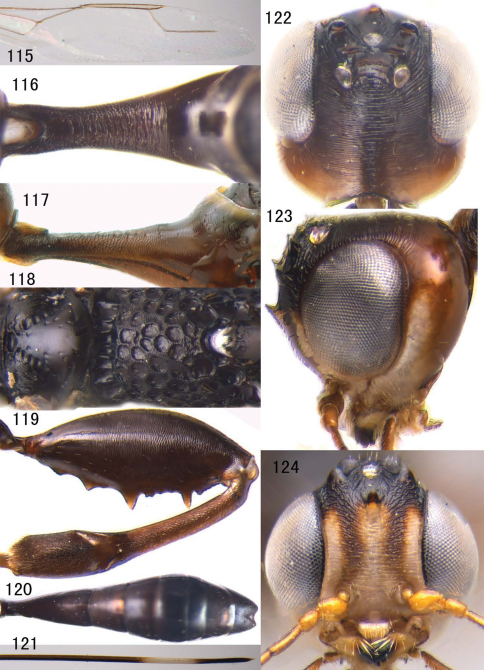
**Figures 115–124.** *Foenatopus flavidentatus* (Enderlein, 1913), female, Guangzhou, Guangdong. **115** fore wing **116** pronotum dorsal **117** pronotum lateral **118** scutellum and propodeum dorsal **119** hind femur and inner side of hind tibia **120** second-eighth tergites dorsal **121** ovipositor sheath **122 **head dorsal **123** head lateral **124** head anterior.

**Plate 15. F16:**
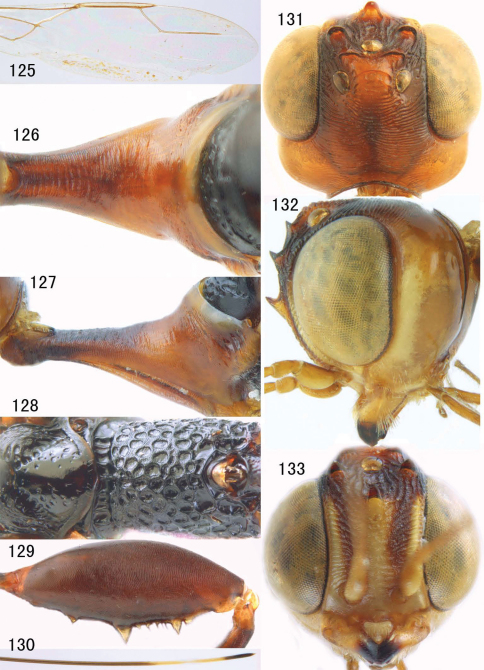
**Figures 125–133.** *Foenatopus flavidentatus* (Enderlein, 1913), lectotype, female. **125** fore wing **126** pronotum dorsal **127** pronotum lateral **128** scutellum and propodeum dorsal **129** hind femur **130** ovipositor sheath **131** head dorsal **132** head lateral **133** head anterior.

**Plate 16. F17:**
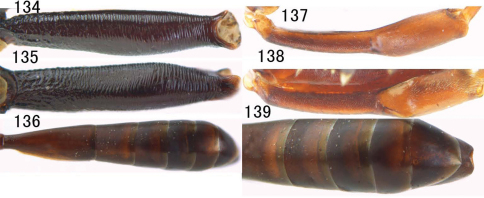
**Figures 134–139.** *Foenatopus flavidentatus* (Enderlein, 1913), lectotype, female. **134** hind coxa dorsal **135** hind coxa lateral **136** second-eighth tergites dorsal **137, 138** inner side of hind tibia **139** pygidial area.

**Plate 17. F18:**
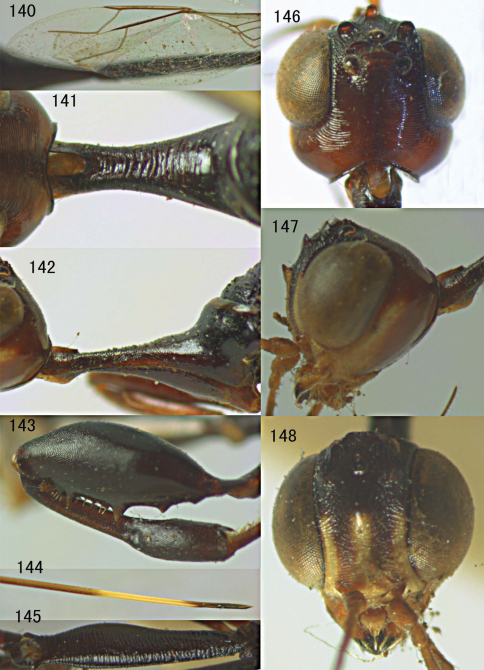
**Figures 140–148.** *Foenatopus simillimus* (Elliott, 1920), holotype, female. **140** fore wing **141** pronotum dorsal **142** pronotum lateral **143** hind femur and outer side of hind tibia **144** ovipositor sheath **145** hind coxa dorsal **146** head dorsal **147** head lateral **148** head anterior.

**Plate 18. F19:**
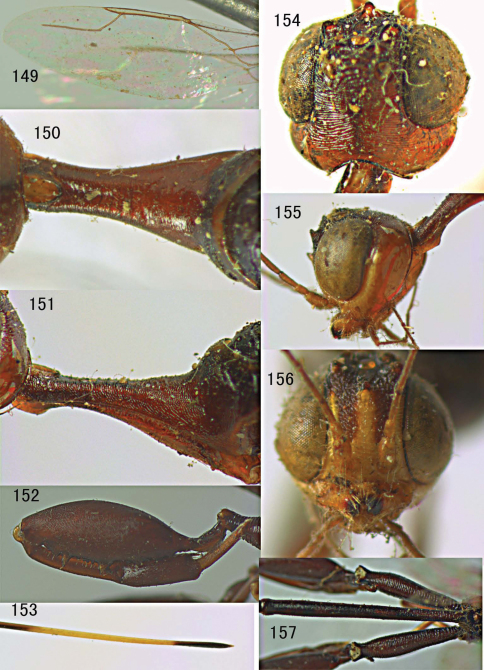
**Figures 149–157.** *Foenatopus trilineatus* (Elliott, 1920), lectotype, female. **149** fore wing **150** pronotum dorsal **151** pronotum lateral **152** hind femur and outer side of hind tibia **153** ovipositor sheath **154** head dorsal **155** head lateral **156** head anterior **157** first tergite dorsal.

**Plate 19. F20:**
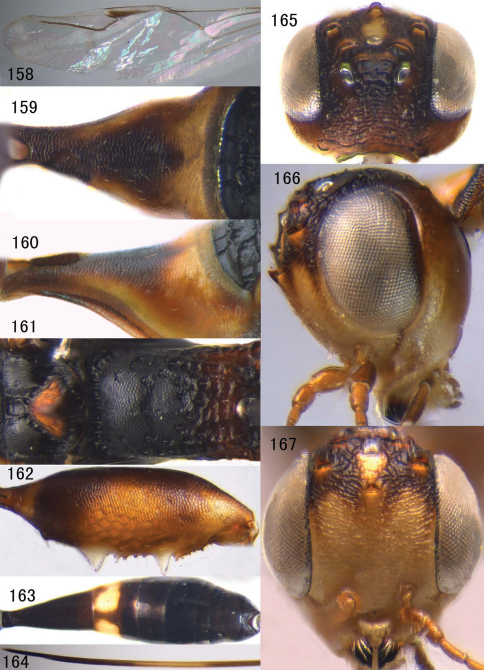
**Figures 158–167.** *Foenatopus maculiferus* sp. n., holotype, female. **158** fore wing **159** pronotum dorsal **160** pronotum lateral **161** scutellum and propodeum dorsal **162** hind femur **163** second-eighth tergites dorsal **164** ovipositor sheath **165** head dorsal **166** head lateral **167** head anterior.

**Plate 20. F21:**
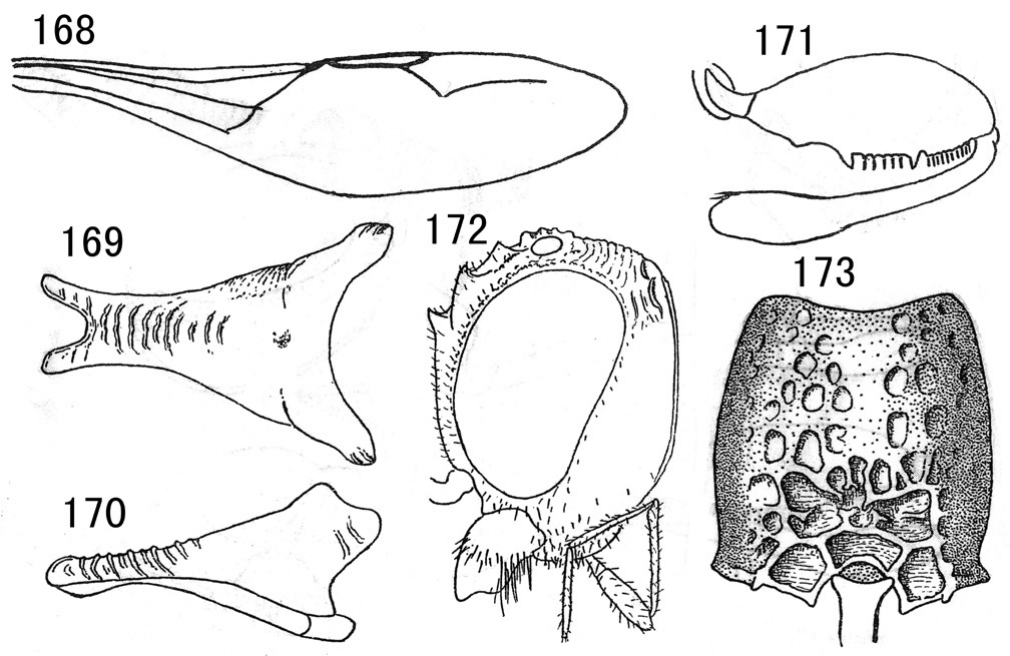
**Figures 168–173.** *Foenatopus menglongensis* (Chao, 1964), holotype, female. **168** fore wing **169** pronotum dorsal **170** pronotum lateral **171** hind femur and outer side of hind tibia **172** head lateal **173** propodeum dorsal. After Chao (1964).

**Plate 21. F22:**
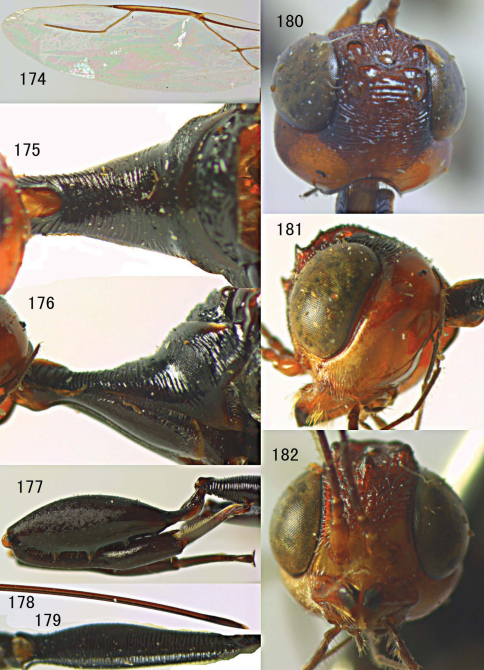
**Figures 174–182.** *Foenatopus quadridens* (Elliott, 1920), holotype, female. **174** fore wing **175** pronotum dorsal **176** pronotum lateral **177** hind femur and outer side of hind tibia **178** ovipositor sheath **179** hind coxa dorsal **180** head dorsal **181** head lateral **182** head anterior.

**Plate 22. F23:**
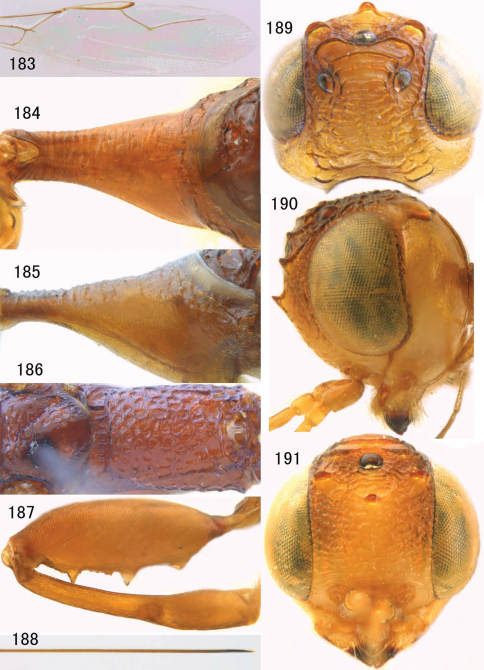
**Figures 183–191.** *Foenatopus ruficollis* (Enderlein, 1913), lectotype, female. **183** fore wing **184** pronotum dorsal **185** pronotum lateral **186** scutellum and propodeum dorsal **187** hind femur and outer side of hind tibia **188** ovipositor sheath **189** head dorsal **190** head lateral **191** head anterior.

**Plate 23. F24:**
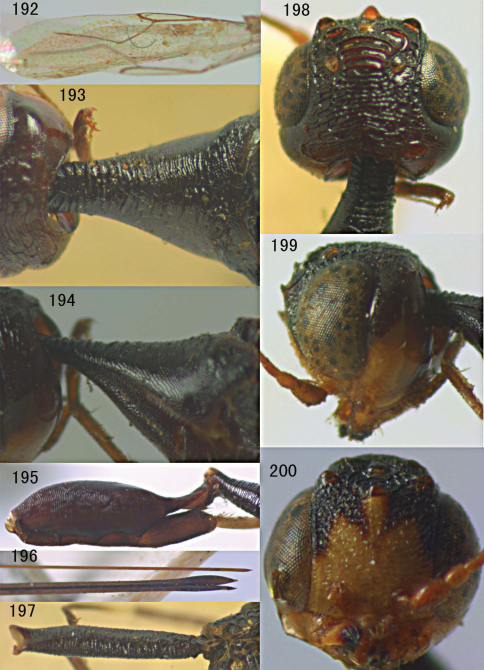
**Figures 192–200.** *Foenatopus trilobatus* Elliott, 1920, holotype, female. **192** fore wing **193 **pronotum dorsal **194** pronotum lateral **195** hind femur and outer side of hind tibia **196** ovipositor sheath **197** hind coxa dorsal **198** head dorsal **199** head lateral **200** head anterior.

**Plate 24. F25:**
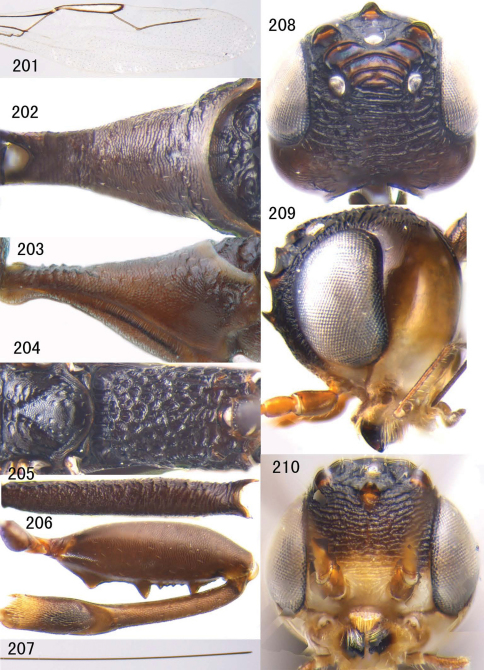
**Figures 201–210.** *Foenatopus trilobatus* Elliott, 1920, female, Arboretum of Nanling, Guangdong. **201** fore wing **202** pronotum dorsal **203** pronotum lateral **204** scutellum and propodeum dorsal **205** hind coxa dorsal **206** hind femur and inner side of hind tibia **207** ovipositor sheath **208** head dorsal **209** head lateral **210** head anterior.

**Plate 25.**
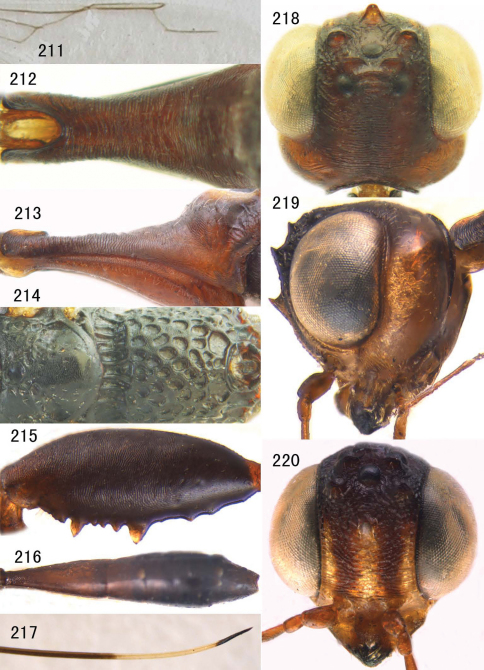
**Figures 211–220.** *Foenatopus yangi* sp. n., holotype, female. **211** fore wing **212** pronotum dorsal **213** pronotum lateral **214** scutellum and propodeum dorsal **215** hind femur **216** second-eighth tergites dorsal **217** ovipositor sheath **218** head dorsal **219** head lateral **220** head anterior.

**Plate 26a.**
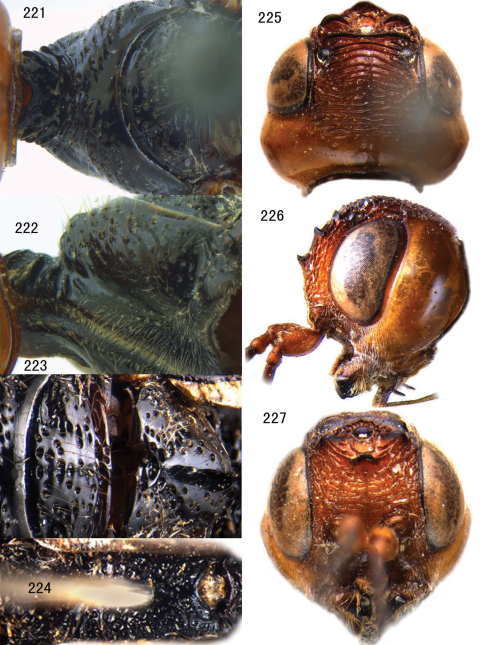
**Figures 221–227.** *Megischus aplicatus* Hong, van Achterberg & Xu, 2010, holotype, male. **221** pronotum dorsal **222** pronotum lateral **223** mesoscutum and scutellum dorsal **224** propodeum dorsal **225** head dorsal **226** head lateral **227** head anterior. After Hong, van Achterberg & Xu (2010).

**Plate 26b. F28:**
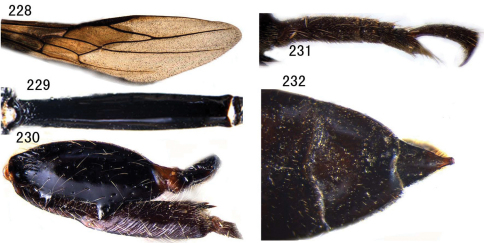
**Figures 228–232.** *Megischus aplicatus* Hong, van Achterberg *&* Xu, 2010, holotype, male. **228** fore wing **229** first tergite **230** hind femur and outer side of hind tibia **231** hind tarsus **232** pygidial process. After Hong, van Achterberg & Xu (2010).

**Plate 27. F29:**
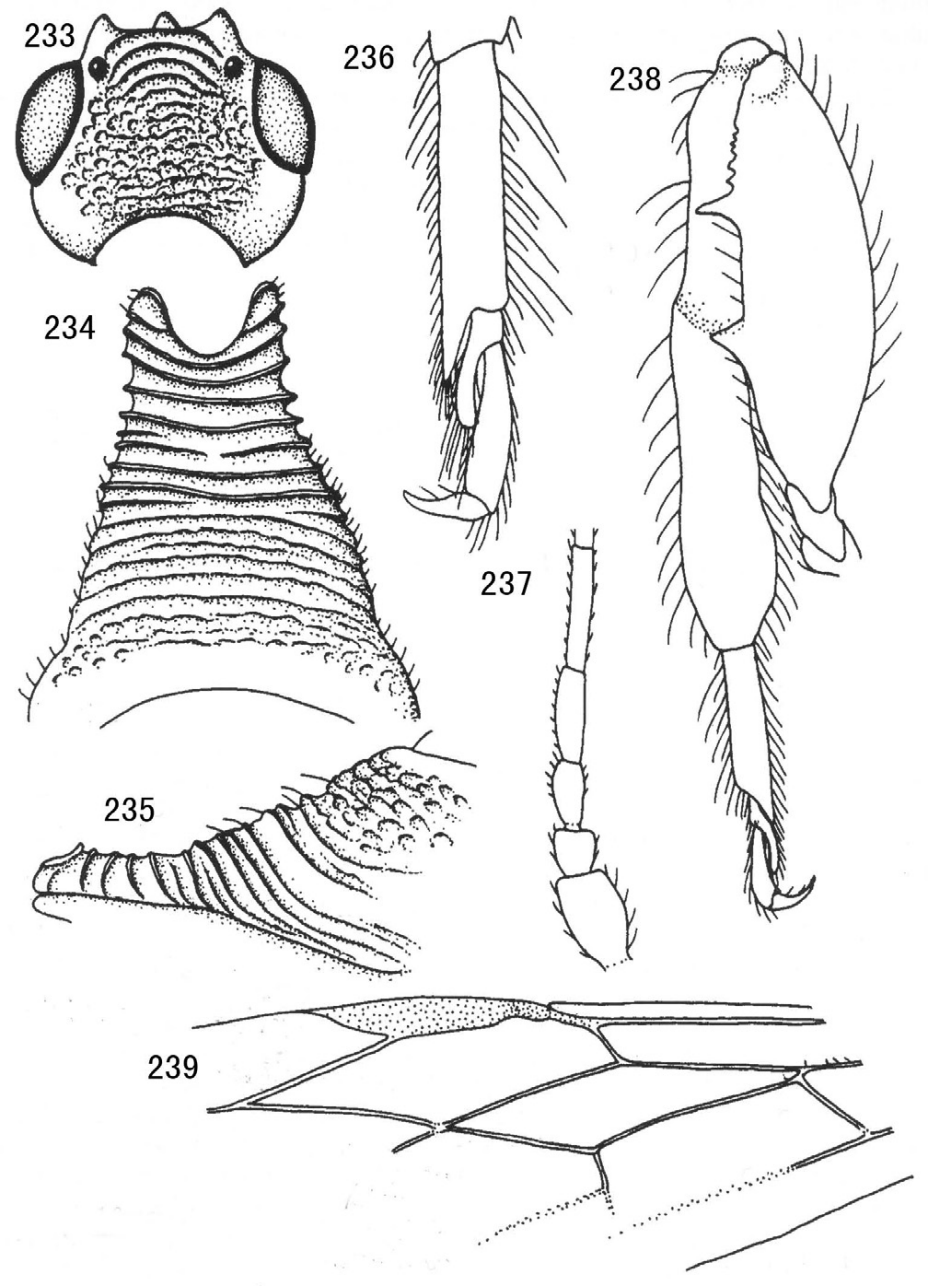
**Figures 233–239.** *Megischus chaoi* van Achterberg & Yang, 2004, holotype, female. **233 **head dorsal **234** pronotum dorsa**l 235** pronotum lateral **236** hind tarsus lateral **237** base of antenna **238** hind leg lateral **239** fore wing. After van Achterberg & Yang (2004).

**Plate 28. F30:**
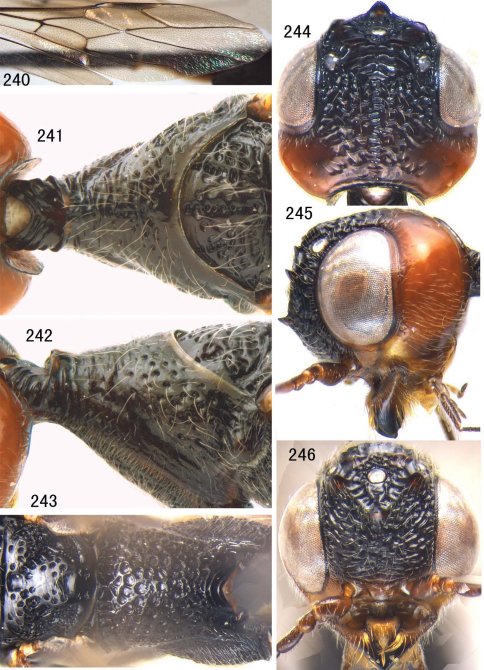
**Figures 240–246** *Megischus ptosimae* Chao, 1964, female, Chebaling, Guangdong. **240** fore wing **241** pronotum dorsal **242** pronotum lateral **243** scutellum and propodeum dorsal **244** head dorsal **245** head lateral **246** head anterior.

**Plate 29.**
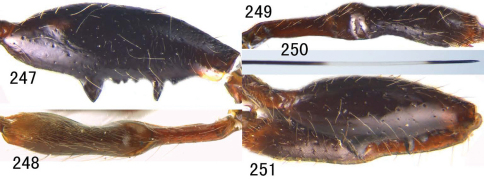
**Figures 247–251.** *Megischus ptosimae* Chao, 1964, female, Chebaling, Guangdong, but **251 **male, Hangzhou, Zhejiang. **247** hind femur **248** inner side of hind tibia **249** outer side of hind tibia **250** ovipositor sheath **251** hind femur and hind tibia (male).

**Plate 30.**
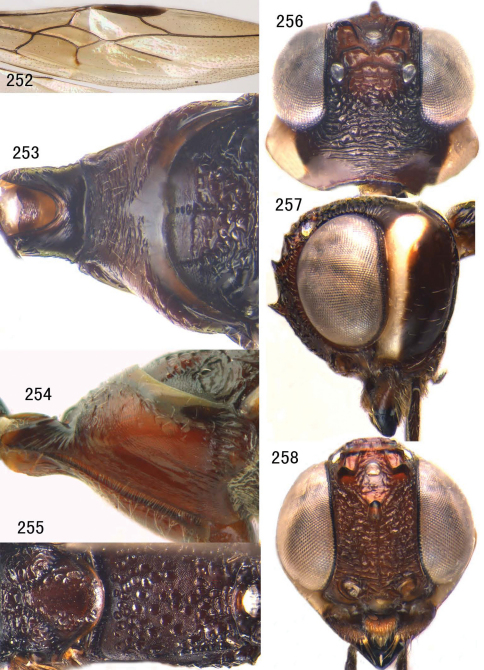
**Figures 252–258.** *Parastephanellus angulatus* sp. n., holotype, female. **252** fore wing **253 **pronotum dorsal **254** pronotum lateral **255** scutellum and propodeum dorsal **256** head dorsal **257 **head lateral **258** head anterior.

**Plate 31. F33:**
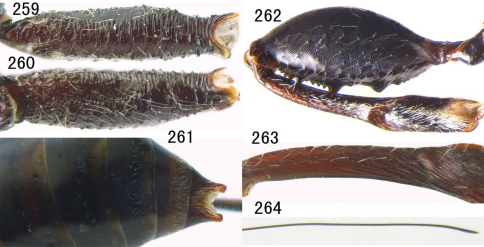
**Figures 259–264.** *Parastephanellus angulatus* sp. n., holotype, female. **259** hind coxa dorsal **260** hind coxa lateral **261** pygidial process **262** hind femur and inner side of hind tibia **263** detail of basal part of hind tibia **264** ovipositor sheath.

**Plate 32. F34:**
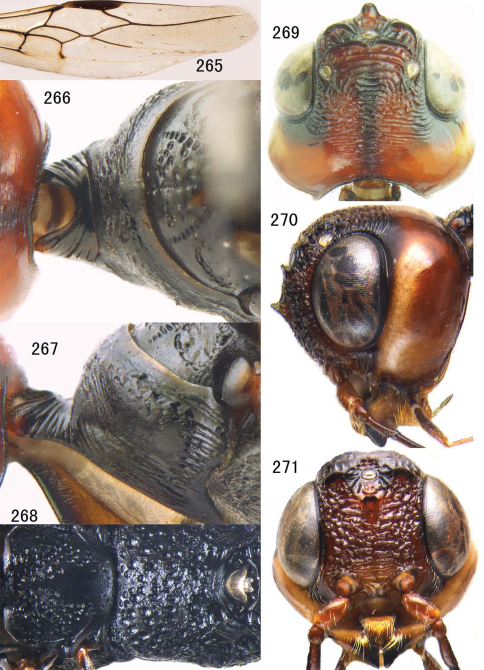
**Figures 265–271.** *Parastephanellus brevicoxalis* sp. n., holotype, female. **265** fore wing **266 **pronotum dorsal **267** pronotum lateral **268** scutellum and propodeum dorsal **269** head dorsal **270 **head lateral **271** head anterior.

**Plate 33. F35:**
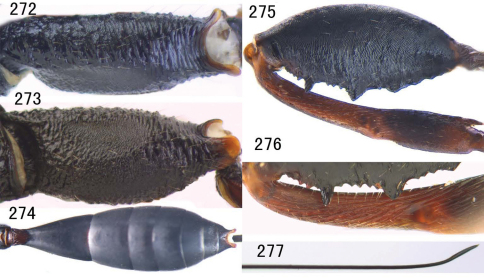
**Figures 272–277.** *Parastephanellus brevicoxalis* sp. n., holotype, female. **272** hind coxa dorsal **273** hind coxa lateral **274** second-eighth tergites dorsal **275** hind femur and outer side of hind tibia **276 **detail of basal part of hind tibia **277** ovipositor sheath.

**Plate 34. F36:**
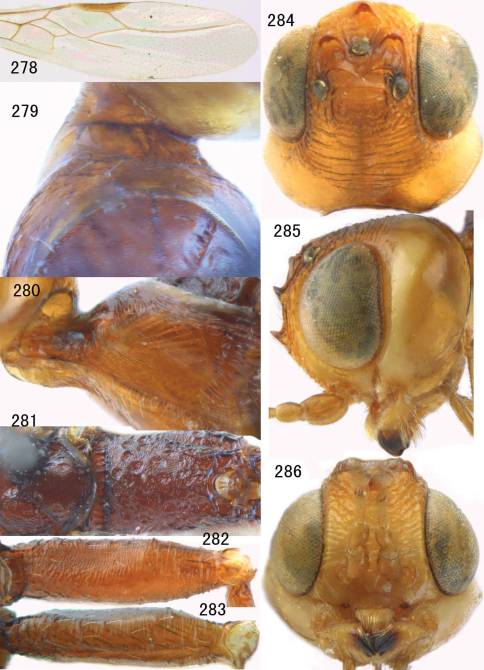
**Figures 278–286.** *Parastephanellus brevistigma* Enderlein, 1913, holotype, male. **278** fore wing **279** pronotum dorsal **280** pronotum lateral **281** scutellum and propodeum dorsal **282** hind coxa lateral **283** hind coxa dorsal **284** head dorsal **285** head lateral **286** head anterior.

**Plate 35. F37:**
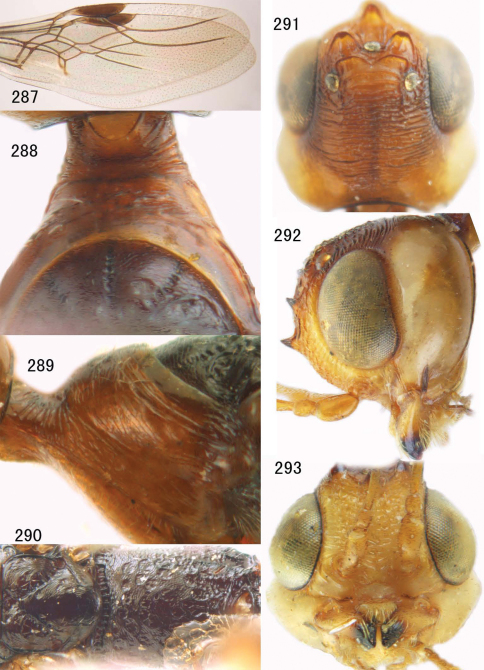
**Figures 287–293** *Parastephanellus brevistigma* Enderlein, 1913, male, Longzhou, Guangxi. **287** fore wing **288** pronotum dorsal **289** pronotum lateral **290** scutellum and propodeum dorsal **291 **head dorsal **292** head lateral **293** head anterior.

**Plate 36. F38:**
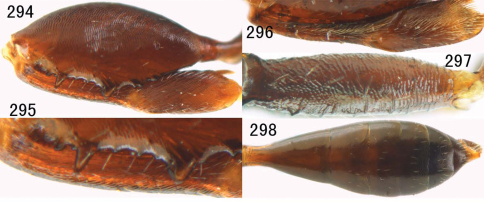
**Figures 294–298.** *Parastephanellus brevistigma* Enderlein, 1913, male, Longzhou, Guangxi. **294** hind femur and outer side of hind tibia **295** detail of basal part of hind tibia **296** inner side of hind tibia **297** hind coxa lateral **298** second-eighth tergites dorsal.

**Plate 37. F39:**
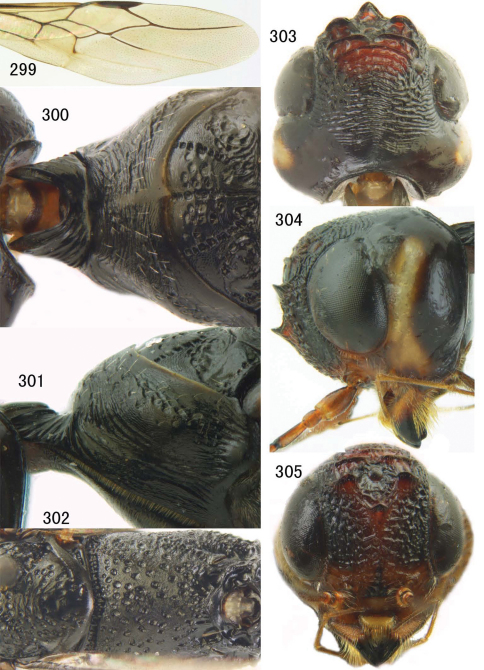
**Figures 299–305.** *Parastephanellus matsumotoi* van Achterberg, 2006, paratype, female. **299 **fore wing **300** pronotum dorsal **301** pronotum lateral **302** scutellum and propodeum dorsal **303 **head dorsal **304** head lateral **305** head anterior.

**Plate 38. F40:**
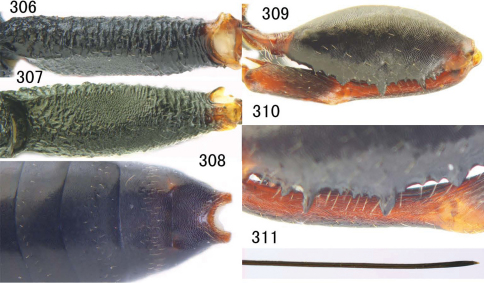
**Figures 306–311.** *Parastephanellus matsumotoi* van Achterberg, 2006, paratype, female. **306 **hind coxa dorsal **307** hind coxa lateral **308** pygidium process **309** hind femur and outer side of hind tibia **310** detail of basal part of hind tibia **311** ovipositor sheath.

**Plate 39. F41:**
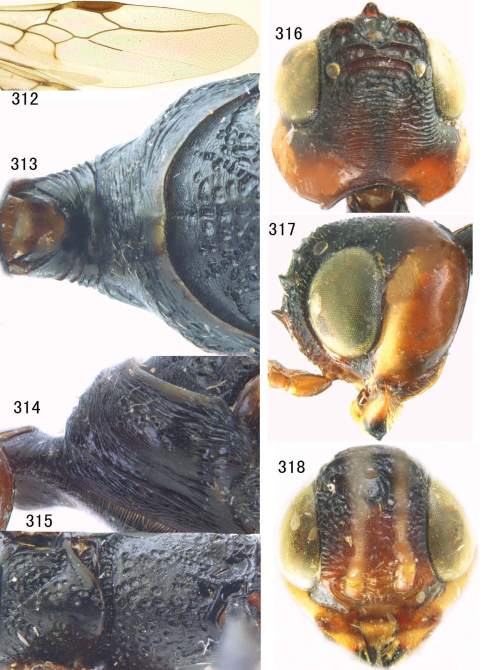
**Figures 312–318.** *Parastephanellus matsumotoi* van Achterberg, 2006, female, Luoshan, Henan. **312** fore wing **313** pronotum dorsal **314** pronotum lateral **315** scutellum and propodeum dorsal **316** head dorsal **317** head lateral **318** head anterior.

**Plate 40. F42:**
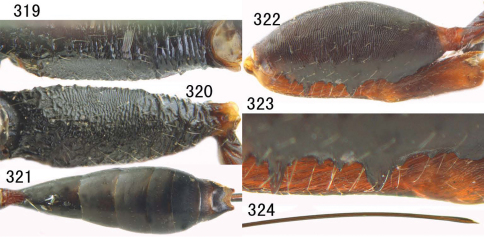
**Figures 319–324.** *Parastephanellus matsumotoi* van Achterberg, 2006, female, Luoshan, Henan. **319** hind coxa dorsal **320** hind coxa lateral **321** second-eighth tergites dorsal **322** hind femur and outer side of hind tibia **323** detail of basal part of hind tibia **324** ovipositor sheath.

**Plate 41. F43:**
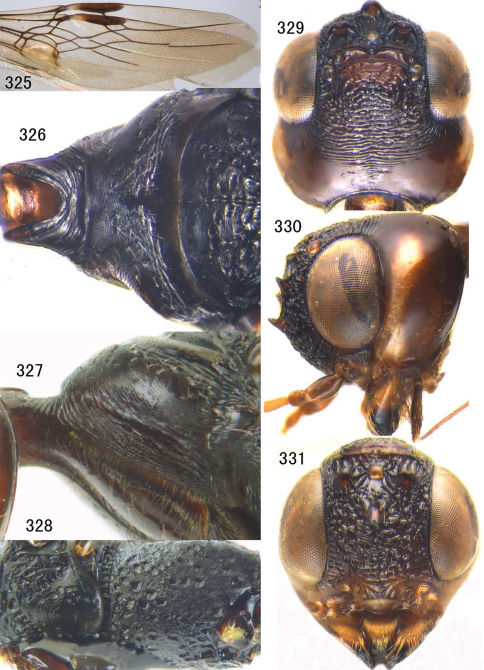
**Figures 325–331.** *Parastephanellus matsumotoi* van Achterberg, 2006, female, Mt. Baiyun, Henan. **325** fore wing **326** pronotum dorsal **327** pronotum lateral **328** scutellum and propodeum dorsal **329** head dorsal **330** head lateral **331** head anterior.

**Plate 42. F44:**
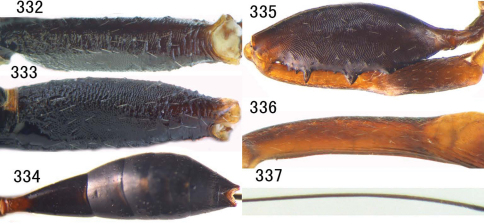
**Figures 332–337.** *Parastephanellus matsumotoi* van Achterberg, 2006, female, Mt. Baiyun, Henan. **332** hind coxa dorsal **333** hind coxa lateral **334** second-eighth tergites dorsal **335** hind femur and outer side of hind tibia **336** detail of basal part of hind tibia **337** ovipositor sheath.

**Plate 43. F45:**
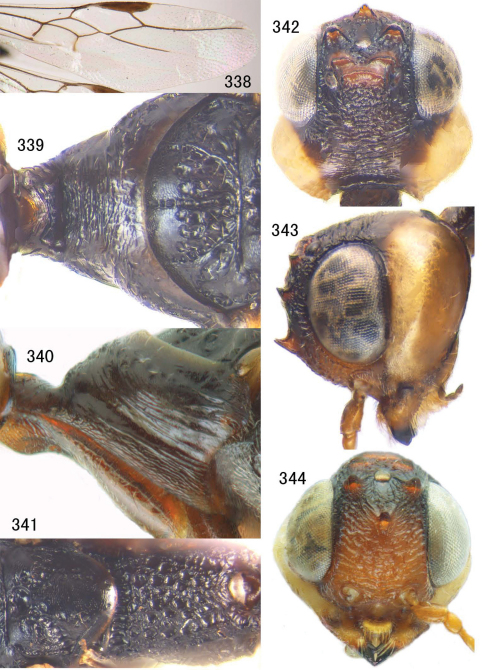
**Figures 338–344.** *Parastephanellus zhejiangensis* sp. n., holotype, female. **338** fore wing **339 **pronotum dorsal **340** pronotum lateral **341** scutellum and propodeum dorsal **342** head dorsal **343 **head lateral **344** head anterior.

**Plate 44.**
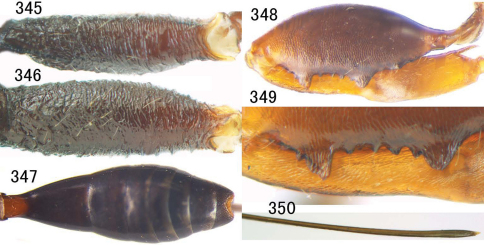
**Figures 345–350.** *Parastephanellus zhejiangensis* sp. n., holotype, female. **345** hind coxa dorsal **346** hind coxa lateral **347** second-eighth tergites dorsal **348** hind femur and outer side of hind tibia **349** detail of basal part of hind tibia **350** ovipositor sheath.

**Plate 45.**
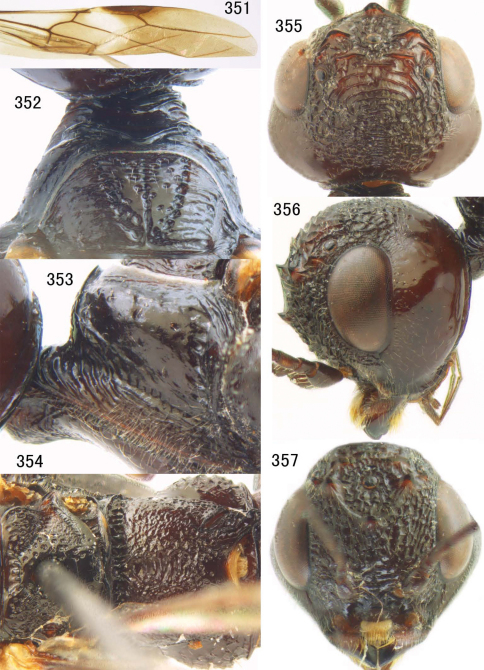
**Figures 351–357.** *Schlettererius cinctipes* Cresson, 1880, female. **351** fore wing **352** pronotum dorsal **353** pronotum lateral **354** scutellum and propodeum dorsal **355** head dorsal **356** head lateral **357** head anterior.

**Plate 46. F48:**
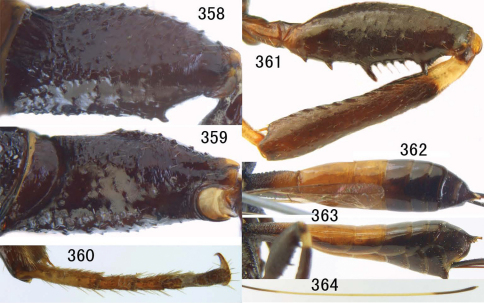
**Figures 358–364.** *Schlettererius cinctipes* Cresson, 1880, female. **358** hind coxa dorsal **359 **hind coxa lateral **360** hind tarsus **361** hind femur and outer side of hind tibia **362** metasoma dorsal **363** metasoma lateral **364** ovipositor sheath.

**Plate 47. F49:**
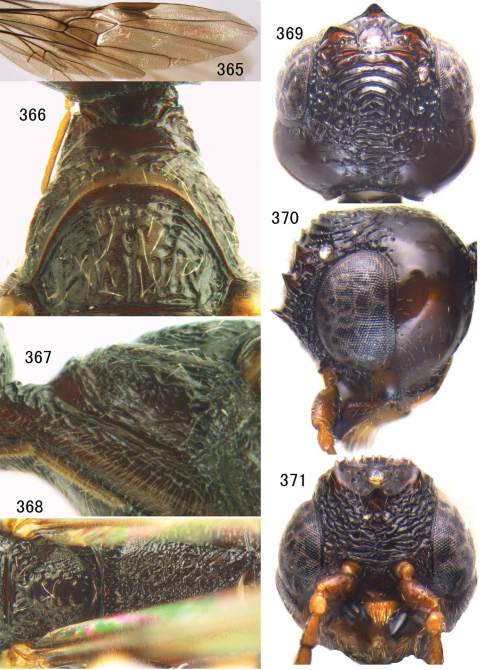
**Figures 365–371.** *Schlettererius determinatorius* Madl, 1991, male, Mt. Zibai, Shaanxi. **365 **fore wing **366** pronotum dorsal **367** pronotum lateral **368** scutellum and propodeum dorsal **369 **head dorsal **370** head lateral **371** head anterior. After Hong and Xu (2011).

**Plate 48.**
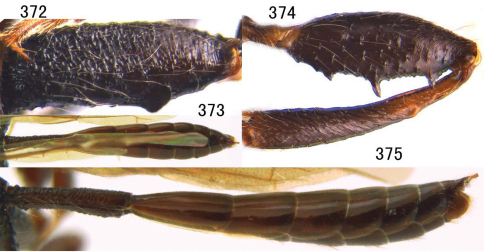
**Figures 372–375.** *Schlettererius determinatorius* Madl, 1991, male, Mt. Zibai, Shaanxi. **372 **hind coxa dorsal **373** metasoma dorsal **374** hind femur and outer side of hind tibia **375** metasoma lateral. After Hong and Xu (2011).

**Plate 49.**
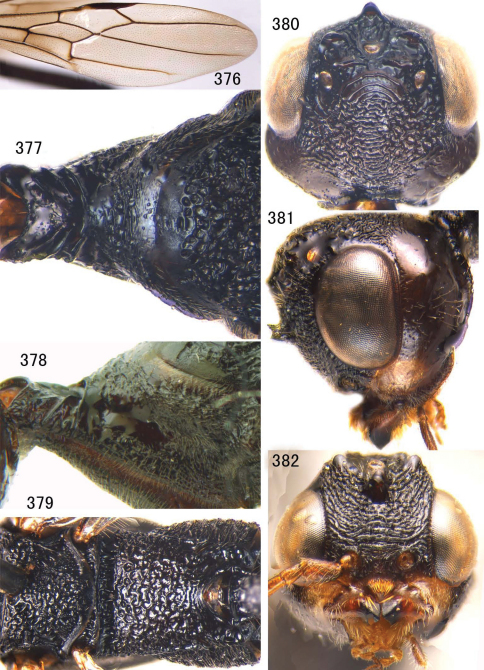
**Figures 376–382.** *Stephanus bidentatus* van Achterberg & Yang, female, Baotianman, Henan. **376** fore wing **377** pronotum dorsal **378** pronotum lateral **379** scutellum and propodeum dorsal **380 **head dorsal **381** head lateral **382** head anterior.

**Plate 50. F52:**
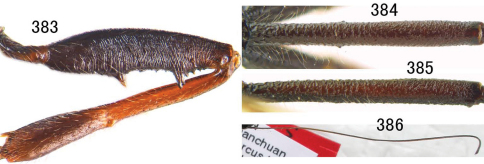
**Figures 383–386.** *Stephanus bidentatus* van Achterberg & Yang, female, Baotianman, Henan. **383** hind femur and inner side of hind tibia **384** first tergite dorsal **385** first tergite lateral **386** ovipositor sheath.

**Plate 51. F53:**
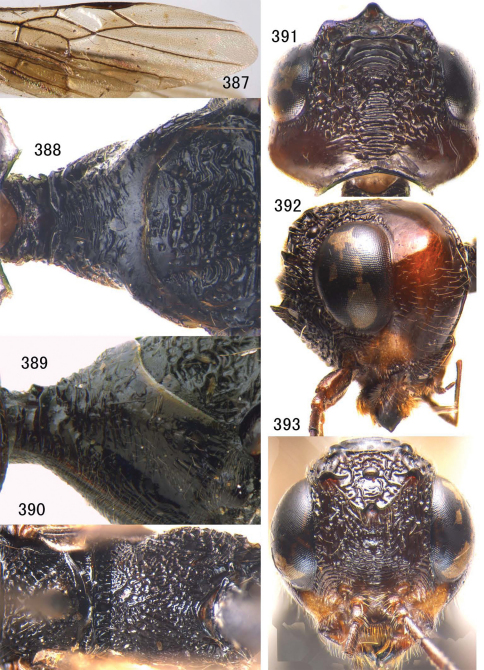
**Figures 387–393.** *Stephanus tridentatus* van Achterberg & Yang, female, Mt. Xinjia, Shaanxi. **387** fore wing **388** pronotum dorsal **389** pronotum lateral **390** scutellum and propodeum dorsal **391 **head dorsal **392** head lateral **393** head anterior.

**Plate 52.**
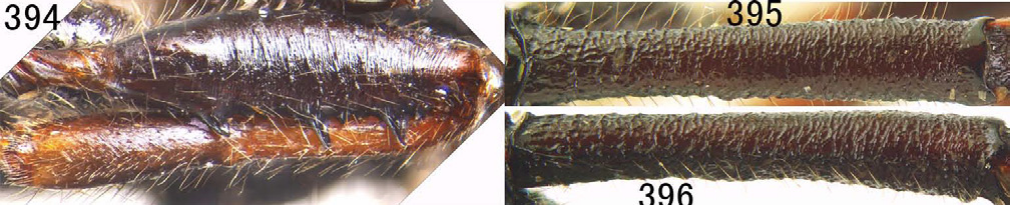
**Figures 394–396.** *Stephanus tridentatus* van Achterberg & Yang, female, Mt. Xinjia, Shaanxi. **394** hind femur and inner side of hind tibia **395** first tergite dorsal **396** first tergite lateral.
